# Influence of *Mycoplasma hyopneumoniae* natural infection on the respiratory microbiome diversity of finishing pigs

**DOI:** 10.1186/s13567-022-01038-9

**Published:** 2022-03-18

**Authors:** Karina Sonalio, Henrique M. S. Almeida, Marina L. Mechler-Dreibi, Gabriel Y. Storino, Freddy Haesebrouck, Dominiek Maes, Luís Guilherme de Oliveira

**Affiliations:** 1 School of Agricultural and Veterinarian Sciences, São Paulo State University (Unesp), Jaboticabal, Brazil; 2 Faculty of Veterinary Medicine, Ghent University, Merelbeke, Belgium

**Keywords:** *M. hyopneumoniae*, microbiota diversity, respiratory tract, lung lesion, swine

## Abstract

*Mycoplasma (M.) hyopneumoniae* interacts with the respiratory microbiota and facilitates colonization of other pathogens. The present study investigated the pulmonary and nasal microbiota of *M. hyopneumoniae*-infected and *M. hyopneumoniae*-free pigs. Sixty-six pigs from three commercial herds were selected at the end of the finishing phase: 44 originated from two *M. hyopneumoniae*-positive herds and 22 from a *M. hyopneumoniae*-negative farm. At the slaughterhouse, samples of nasal turbinate (NT) and bronchus-alveolar lavage fluid (BALF) were collected. DNA was extracted with a commercial kit and the infection status was confirmed by qPCR. All samples from the same herd were pooled, and next-generation sequencing based on the hypervariable region V3–V4 of the 16 s bacterial rDNA was performed. Data analysis included the taxonomic analysis, Alpha diversity indexes, and Principal coordinates analysis (Pcoa) using Jaccard, Bray–Curtis, Weighted Unifrac, and Unweighted Unifrac distances. All pigs from the infected herds tested PCR positive for *M. hyopneumoniae*, whereas all pigs from the negative farm were negative. There was a greater diversity of microorganisms in BALF when compared to NT samples in all the farms. BALF samples from infected animals showed higher abundance of *M. hyopneumoniae* than NT samples and a predominance of *Pasteurella multocida* among the main species identified, which was also abundant in the *M. hyopneumoniae*-free herd. PCoa diagrams indicated that for most of the samples, dissimilarity on bacterial composition was observed, regardless of infection status and sample type. Therefore, the lung microbiota was modulated by *M. hyopneumoniae* infection, which could play a role in the pathogenesis of *M. hyopneumoniae*-disease.

**Supplementary Information:**

The online version contains supplementary material available at 10.1186/s13567-022-01038-9.

## Introduction

Mycoplasmas are small bacteria without a cell wall that affect several animals, including pigs. Different species are known to infect swine, among them *Mycoplasma (M.) hyopneumoniae*, *Mycoplasma (M.) flocculare*, and *Mycoplasma (M.) hyorhinis*. *Mycoplasma hyopneumoniae* is considered the primary agent of Swine Enzootic Pneumonia (SEP), a disease that results in chronic pneumonia, non-productive dry cough, growth retardation, and a negative impact on productive indicators [1]. Besides, *M. hyopneumoniae* can also play an important role in the Porcine Respiratory Disease Complex (PRDC), a multifactorial disorder where several infectious agents can be involved, such as porcine reproductive and respiratory syndrome virus (PRRSv), porcine circovirus type 2 (PCV2), and *Pasteurella* (*P.*) *multocida* [1, 2].

*Mycoplasma hyopneumoniae* infections are commonly associated with a decrease in performance and an increase in costs related to control and treatment [3, 4]. The bacteria colonize the ciliated respiratory epithelium, promoting an exacerbated inflammatory response, which generates lesions that favor opportunistic pathogens, like *Actinobacillus (A.) pleuropneumoniae*, *Streptococcus (S.) suis*, *P. multocida*, and *Glaesserela (G.) parasuis* [1, 3].

Although considered commensal bacteria in the respiratory tract of swine, *M. hyorhinis* has shown to be associated with *M. hyopneumoniae* infection and PRDC [1, 5]. In addition, it is a well-known cause of polyserositis and polyarthritis in piglets, and less often is associated with pneumonia, otitis, conjunctivitis, and abortion [1]. Recently, *M. flocculare*, another commensal bacterium in swine, has shown a negative correlation with the extension of macroscopic lung consolidation lesions (MLCL) when associated with *M. hyopneumoniae* infection [5]. Since the detection of one decreased the probability of detecting the other, it is possible that *M. hyopneumoniae* infection changes the environment in the respiratory tract, thereby creating unfavorable conditions for *M. flocculare* [5, 6].

Studies have shown higher abundance of *M. hyopneumoniae*, along with other bacterial genera (*Ureaplasma*, *Glaesserella*, and *Phyllobacterium*) in the microbiome of macroscopically affected lungs, when compared to healthy lungs, where the main genera reported were *Methylotenera*, *Prevotella*, *Sphingobium*, and *Lactobacillus* [7]. Additionally, *M. hyopneumoniae* was positively associated with the extent of the lung lesions and reported as a taxonomic driver of functional shifts in the lungs with severe lung lesions [7]. Other authors have also reported an apparent predominance of *M. hyopneumoniae*, *M. flocculare*, and *M. hyorhinis* in the lungs of pigs affected by enzootic pneumonia [8, 9]. Despite little information on the respiratory microbiota of pigs, and how the different species of microorganisms interact with each other, it is known that the diversity of bacterial species has been associated with greater stimulation of the immune system and strengthening of the non-specific immune response, both locally and systemically [10]. Thus, knowing how these interactions occur is essential to better address the control and prevention of co-infections, either bacteria-bacteria or bacteria-viruses [11].

As reported by Siqueira et al. [8], *M. hyopneumoniae* was confirmed with a high prevalence when referring to community composition in lungs. Moreover, the authors suggest that the interactions among respiratory pathogens are numerous and complex, and therefore, can be additive or synergistic. In general, populations of microorganisms in organs of healthy individuals can help their functions and/or prevent colonization by pathogens. As an example, *Lactobacillus* spp. contribute to the formation of alveoli and mucus, and consequently they improve the functioning and protection of the respiratory tract [12].

Considering the abundance of *M. hyopneumoniae* and other bacterial species in the respiratory tract of pigs, the present study investigated possible differences between the pulmonary and nasal microbiota of finishing pigs naturally infected with *M. hyopneumoniae* and *M. hyopneumoniae*-free pigs in order to better understand the interactions and pathogenesis.

## Materials and methods

### Experimental design and sample collection

This study was submitted to the Ethics Committee in Animal Use of the Faculty of Agricultural and Veterinary Sciences of the São Paulo State University—Jaboticabal, and approved under the Protocol #2032/21.

Three commercial swine herds (H), located in the state of São Paulo—Brazil, were selected for the study: one herd was free of *M. hyopneumoniae* (H1) and the other two were infected with *M. hyopneumoniae* (H2 and H3), as reported by the herd veterinarian and the presence of MLCL, suggestive of enzootic pneumonia. Moreover, the *M. hyopneumoniae*-status of the three farms was confirmed by testing bronchus-alveolar lavage fluid (BALF) and nasal turbinate (NT) samples with qPCR [5, 6]. The free farm is a nucleus breeding herd of 650 sows, farrow-to-finish system, free of *A. pleuropneumoniae* and with vaccination against Influenza virus, Porcine Circovirus, Parvovirus, Leptospirosis, Erysipelas, and *G. parasuis*. The infected animals originated from two different commercial farms of 550 and 600 sows, farms H2 and H3, respectively. These two farms also used farrow-to-finish systems, with vaccination against *M. hyopneumoniae*, *P. multocida*, Influenza virus, Porcine Circovirus, Parvovirus, Leptospirosis, Erysipelas, and *G. parasuis.* The vaccination protocol is described in Additional file 1. Regarding antimicrobial use, although the animals from all farms were sporadically treated with antimicrobials in the feed until 75 days of age (amoxicillin, tiamulin, doxycycline and/or florfenicol), they did not receive any antimicrobial treatment for at least 2 months before slaughter. Natural ventilation and concrete floor with water blade were used for the finishing pigs in all farms. In addition, all animals were from the same breed.

From each farm, 22 pigs (140-day-old average) were randomly selected and identified by an ear tag in the morning. The pigs were sent to the slaughterhouse later on the same day. Animals from the same farm were kept in the same rest pen for at least 3 h before slaughter, and animals from H2 and H3 were not mixed with the *M. hyopneumoniae*-free pigs (H1). However, the rest pens were located in the same space, side by side, and separated by 1.5 m height concrete walls. Animals with *M. hyopneumoniae*-free status were the first to be slaughtered (H1), followed by the pigs from the *M. hyopneumoniae-*infected farms (H2 and H3).

At the slaughterhouse, the corresponding head and lungs from the selected carcasses were separated for evaluation and sampling. The nose of the pigs was sawn off in the slaughter-line using a saw (disinfected by water at 90 °C) to access the NT. Fragments of the NT were collected directly from the carcass using sterile forceps and scalp. Then, the fragments were inserted into cryotubes free of DNAse and RNAse (Corning, USA) and stored in a freezer at −80 °C.

BALF was collected using a plastic pipette and automatic pipettor. For insertion of the pipette, an incision was made in the trachea, about 5 cm before the bifurcation of the bronchi. Directly into the bronchi, 20 mL of PBS (1×, pH 7.4; Sigma-Aldrich, Germany) was dispensed, followed by a gentle massage through the lung parenchyma and aspiration with a pipette, recovering between 10 and 15 mL of lavage. The recovered content was transferred to 50 mL falcon tubes (Corning, USA) and later aliquoted into 2 mL cryotubes free of DNAse and RNAse (Corning, USA), which were then stored in a freezer at −80 °C until further processing.

### Macroscopic lung lesion scoring

At the time of slaughter, the lungs of the study pigs were removed from the carcass and the macroscopic lung lesions compatible with SEP were scored according to the methodology described by Straw et al. [13]. The apical, cardiac, accessory and diaphragmatic lobes were evaluated separately. The scores could range from 0 (no lesions) to 100% (entire lung affected).

### DNA extraction

BALF samples were thawed and centrifuged at 4 °C (Centrifuge 5804 R, Eppendorf, Germany) at 12 000 *g* for 20 min. After centrifugation, the supernatant was discarded and the pellet was used for DNA extraction. A total of 0.05 g of NT and the pellet from BALF samples were individually submitted to commercial DNA extraction using the DNeasy blood and Tissue kit (Qiagen, USA), following the manufacturer’s instructions. The extraction product was evaluated by spectrophotometry in Nanodrop^®^ 2000c (Thermo Fisher, USA) to measure concentration and purity.

### qPCR targeting the *M. hyopneumoniae p102*, *M. hyorhinis p37* and *M. flocculare fruA* gene fragments

Gene fragments of *M. hyopneumoniae (p102), M. hyorhinis (p37),* and *M. flocculare (fruA)* from DNA samples were quantified by qPCR as described elsewhere [6], with modifications [5]. The standard curve with serial dilutions of constant ratio equal to 10 (10^7^–10^1^) was based on a synthetic DNA (GBlock^®^, IDT, USA) containing fragments of 150 base pairs (bp) (*p102*), 101 bp (*p37*) and 119 bp (*fruA*), used as positive control [5]. All samples were tested in duplicate, and the results were only accepted for those with a standard deviation lower than or equal to 0.5 cycle [14]. Samples with a deviation greater than 0.5 were retested in triplicates.

### Pooling and amplification of bacterial 16 s rDNA

The extracted DNA samples from animals of the same herd (H1, H2, or H3) were grouped in equimolar amounts according to the concentration (ng∕µL) obtained by spectrophotometry. The pools from the control group were named H1NT and H1BALF, while the pools of samples from infected farms were named H2NT, H2BALF, and H3NT, H3BALF. After pooling and before submission to Illumina sequencing, the integrity and quality of the samples were evaluated using Bioanalizer^®^ (Agilent -CA, USA).

Pooled DNA samples were submitted to a PCR targeting the hypervariable region V3-V4 of the 16 s bacterial rDNA, using a previously described protocol [15], which flanks an amplicon of 465 bp. Then, Illumina sequences were added to the original primer sequence following the manufacturer’s instructions. The sequences were: F: 5′-TCGTCGGCAGCGTCAGATGTGTATAAGAGACAGCCTACGGGNGGCWGCAG-3′, and R: 5′-TCGTCGGCAGCGTCAGATGTGTATAAGAGACAGCCTACGGGNGGCWGCAG—3′. After amplification, integrity and production were evaluated by electrophoresis on a 2% agarose gel (Sigma-Aldrich, Germany) stained with SYBR gold (0.5 µg/mL) (Invitrogen^®^, USA).

### Library preparation and sequencing

Library validation was performed in a Bioanalyzer 2100 (Agilent Technologies, USA) and quantified using the Kappa Library Quant Kit for Illumina (Illumina, USA), following the manufacturer’s instructions. The library was then adjusted for equimolar concentration according to the manufacturer’s instructions [16]. Then, 5 µL of each diluted library were mixed in a pool and submitted to Next-Generation Sequencing (NGS).

Sequencing was performed using the MiSeq Reagent Kit v3 (600 cycles) (Illumina, USA) on an Illumina MiSeq platform (Illumina, USA), according to Caporaso et al. [17]. The generated sequencing data were demultiplexed using Illumina bcl2fastq software (v2.19.1.403), and sequencing quality assessment was performed using DADA2 software [18]. Briefly, adapter sequences and end low quality reads were trimmed, removing the first 15 nucleotides of reads (forward and reverse) and cutting read ends at position 270. Trimmed reads were then filtered out with more than two expected errors. Both pair of reads (forward and reverse) were removed when one was discarded. Then, DADA2 ran denoising by collapsing together all reads that encoded the same sequence, and merged forward and reverse reads using alignment. Pairs of reads that did not match were discarded. Lastly, DADA2 removed chimera sequences. The parameters used for DADA2 analysis were: –p-trim-left-f 15 –p-trim-left-r 15 –p-trunc-len-f 270 –p-trunc-len-r270.

### Data analysis

Correlations between MLCL and quantification of each *Mycoplasma* spp. were evaluated according to the normality of the data, where Spearman’s Rank correlation test was used for non-parametric data (*p* < 0.05). To measure the strength of dependence between two variables, the Kendall’ rank correlation test was used (*p* < 0.05). The R software version 3.5.1 (R Core Team, 2018) was used for data analysis.

The contigs were aligned and assembled in silico using the software Quantitative Insights Into Microbial Ecology—QIMEE version 2019.10 [19] and the SILVA database (release 132), reducing to the level of phyllo, family, and genera. For the analysis of bacterial diversity, a phylogenetic tree was constructed using the Mafft software for multi-sequence alignment and the FastTree software, both following the standard parameters. For alpha diversity’s analyses, the following metrics were evaluated: OTUs quantity, Faith, Shannon, Pielou, and Simpson indexes. Main coordinates analysis was performed to detect similarities between uninfected (H1) and infected herds (H2 and H3), based on the following metrics: Jaccard, Bray–Curtis, Weighted Unifrac, and Unweighted Unifrac. For each metric, differences between infection status, tissue, tissue and infection status were calculated using multivariate permutational analysis of variance (PERMANOVA) at the significance level of *p* < 0.05.

## Results

### *Mycoplasma* detection and quantification in NT and BALF samples

The efficiency of qPCR reactions varied from 92.3% to 101.4%, R^2^ ranged from 0.992 to 0.999; the slope varied from −3.387 to −3.522. Detailed parameters are shown in Additional file 2. All BALF samples from H1 were negative for *M. hyopneumoniae* on the qPCR while 21 samples (95.4%) from H2 and all samples (100%) from H3 were positive. For NT, all samples from H1 were *M. hyopneumoniae*-negative while 8 samples (36.4%) from H2 and 4 samples (18.2%) from H3 were *M. hyopneumoniae*-positive. One animal in H2 tested negative for *M. hyopneumoniae* in the BALF sample and tested positive in the NT sample, and therefore, is considered *M. hyopneumoniae*-positive. Among all NT samples, 81.8% (54/66) tested positive for *M. flocculare* and 59.1% (39/66) were positive for *M. hyorhinis*. Similarly, 80.3% (53/66) and 53% (35/66) of the BALF samples tested positive for *M. flocculare* and *M. hyorhinis*, respectively.

Median quantification values for *M. hyopneumoniae* in BALF and NT were 8.73 × 10^5^ copies/μL (2.98 × 10^2^ to 4.51 × 10^7^) and 4.20 × 10^2^ copies/μL (9.51 × 10^0^–4.20 × 10^3^), respectively. Detailed information regarding quantification data are summarized in Additional file 3. In total, 17 samples could not be quantified due to inconsistent quantification results (Cq values with a difference > 0.5), even when tested in triplicate. This fact is likely due to the Markov Chain Monte Carlo effect [14], which represents an inherent limitation of the qPCR technique, mainly in samples with a low number of DNA copies. Even though these samples could not be quantified, they were considered positive.

### Correlations with *Mycoplasmas* quantification and macroscopic lung lesion score

The mean macroscopic lung lesion scores obtained from the different herds were: H1 − 0.5225 (SD = 2.58); H2 − 6.6025 (SD = 14.34); and H3 − 2.7075 (SD = 6.25). Significant differences in the mean macroscopic lesion score were detected between *M. hyopneumoniae*-positive (H2 and H3) and *M. hyopneumoniae*-negative (H1) farms (*p* < 0.05). MLCL occurrence for H1, H2, and H3 was 68.18%, 90.9%, and 90.9%, respectively.

Spearman’s correlation showed a significant positive correlation (r = 0.62; *p* < 0.05) between the quantification of *M. hyopneumoniae* in the BALF by qPCR and the macroscopic lung lesions. No significant correlations were observed between macroscopic lung lesions and *M. hyorhinis* or *M. flocculare* quantification results in BALF samples (*p* > 0.05). There were no significant correlations between macroscopic lung lesions and *M. hyopneumoniae*, *M. hyorhinis*, or *M. flocculare* quantification results in NT samples (*p* > 0.05).

Kendall’s rank correlation test showed a negative correlation between the quantification of *M. hyopneumoniae* and *M. flocculare* in BALF (r = −0.43; *p* < 0.05), and no significant correlation between the quantification of *M. hyopneumoniae* and *M. hyorhinis* (*p* = 0.89). There was a significant positive correlation between the quantification of *M. hyopneumoniae* in BALF and NT samples (*p* < 0.05; rho = 0.4).

### NGS results and quality assessment

The NGS identified a total of 4364 features or operational taxonomic unit (OTUs) with sequence sizes ranging from 255 to 498 bp, and an average sequence size of 377.7 bp (± 68.8). The OTUs and readings obtained for each pool before rarefaction are shown in Table 1.

**Table 1:** **NGS data and quality control obtained from pooled NT and BALF samples of the three farms**.

Group	Sample type	Sample ID	OTUs	Number of reads
*M. hyopneumoniae*-free (H1)	NT	H1NT	790	590 252
	BALF	H1BALF	182	181 176
*M. hyopneumoniae*-positive (H2 and H3)	NT	H2NT	1384	1 242 750
		H3NT	1218	705 578
	BALF	H2BALF	1585	1 914 416
		H3BALF	481	1 229 248

### Diversity and species richness in NT and BALF samples

To estimate the species diversity in the samples, the sequences were rarefied to a depth of 69 100 reads. The samples showed varying numbers of reads, with H2NT and H2BALF showing the highest values, followed by H3 samples, both from infected herds (Table 1). The higher sequencing throughput in both H2 samples was reflected in higher OTU values. A high OTU value was also observed for the H3NT sample, however, the H3BALF sample presented a low OTU value, resulting in lower diversity values. The H1BALF sample had a lower number of OTUs, possibly due to the low sequencing throughput (181 176 reads). Detailed information is presented in Table 1.

Regarding the alpha diversity metrics calculated for each pool, the OTU values for NT samples were numerically higher (average: 987), indicating, in general, a greater diversity of genders when compared to BALF samples (average: 572.6). In addition, Shannon values for BALF samples from infected animals were numerically lower (H2: 2.77 and H3: 1.68), indicating low diversity. Considering the uniformity aspect by the Pielou and Simpson metrics, the samples H2BALF and H3BALF showed the lowest values, indicating less uniformity between genera in the sample (Table 2).

**Table 2:** **Alpha diversity metrics obtained from pooled NT and BALF samples of the three farms**.

Group	Sample ID	OTUs	Faith	Shannon	Pielou	Simpson
*M. hyopneumoniae-*free (H1)	H1NT	710	356.93	3.37	0.35	0.64
	H1BALF	182	63.08	3.79	0.50	0.87
*M. hyopneumoniae-*positive (H2 and H3)	H2NT	1120	533.08	4.18	0.41	0.79
	H3NT	1131	552.68	5.04	0.49	0.84
	H2BALF	1200	134.61	2.77	0.27	0.58
	H3BALF	336	122.79	1.68	0.20	0.48

### Principal coordinates analyses (PCoa) of NT and BALF

#### Bray–Curtis dissimilarity distance

**Figure 1:**
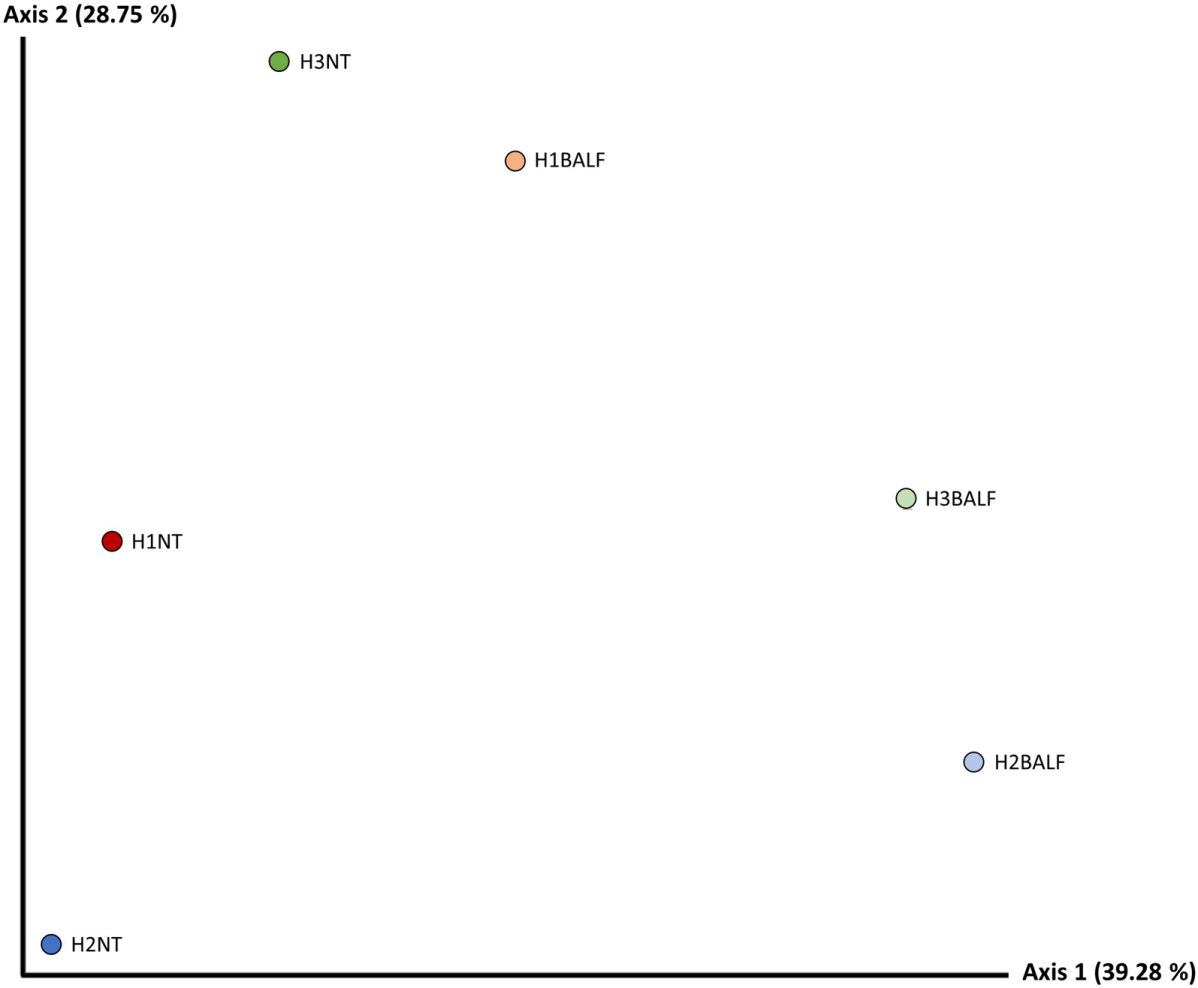
**Bray-Curtis dissimilarity distance Pcoa diagram based on NT and BALF pooled samples of the *M. hyopneumoniae*-infected (H2 and H3) and *M. hyopneumoniae*-free (H1) herds.**

The PCoa constructed based on the Bray–Curtis distance showed that there was no separation between samples from different herds in any of the axes. Considering the PCoa analysis for tissue, it was observed that samples from different tissues appear separated in axis 1, with no cluster formation, but certain proximity between H2BALF and H3BALF, and greater dissimilarity in relation to the other samples (Figure 1).

#### Jaccard distance

The PCoa based on Jaccard’s distance did not show proximity between any samples. Visually, a greater dissimilarity was observed for the H2BALF sample in relation to the others (data not shown).

#### Unweighted Unifrac distance

**Figure 2:**
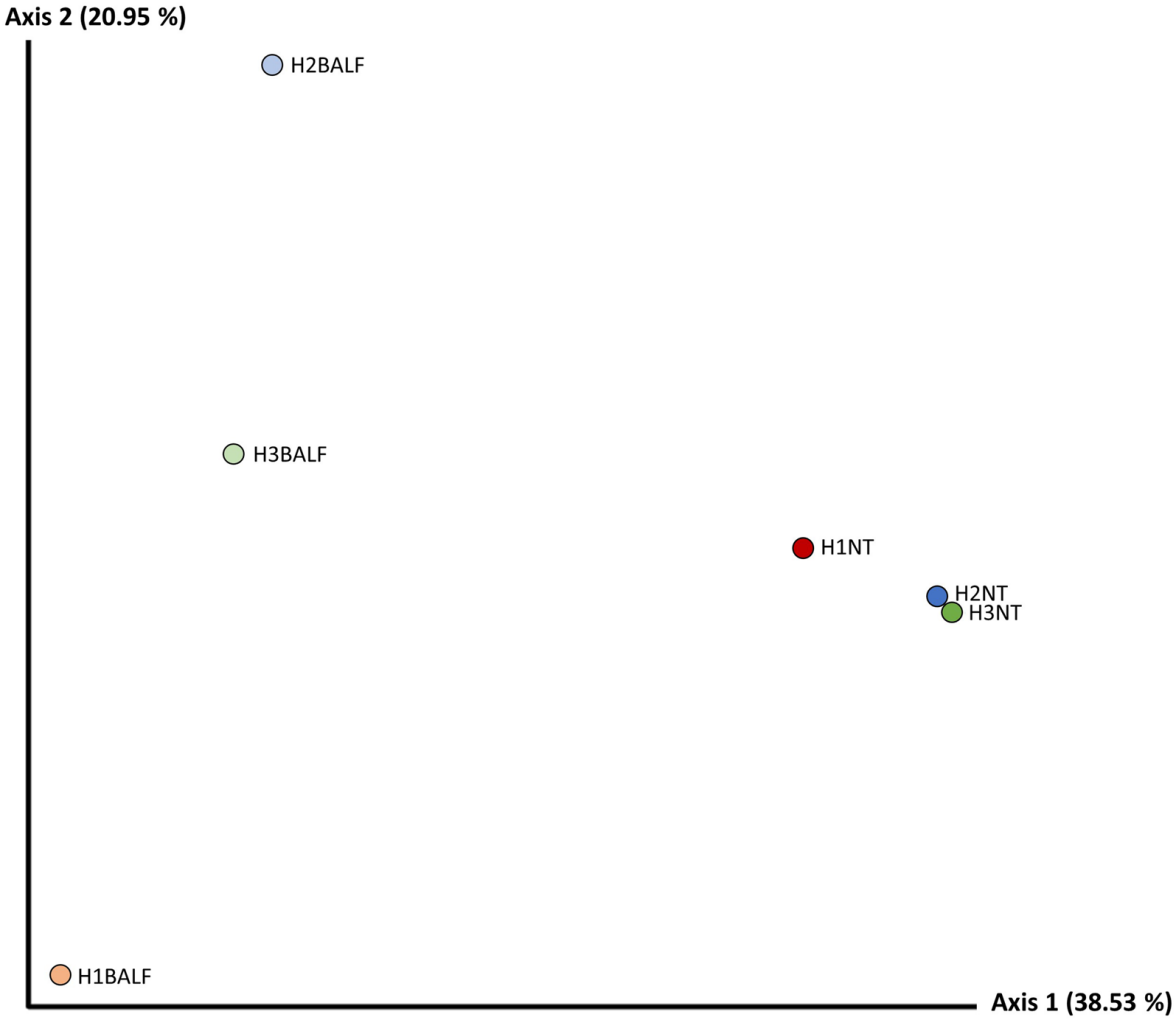
**Unweighted Unifrac distance Pcoa diagram based on NT and BALF pooled samples of the* M. hyopneumoniae*-infected (H2 and H3) and *M. hyopneumoniae*-free (H1) herds.**

The PCoa based on the unweighted Unifrac distance showed separation between BALF and NT samples, with greater dissimilarity for NT samples on axis 1. The H2NT and H3NT samples were close to each other, indicating qualitative similarity between the bacterial types present in both samples. Regarding BALF samples, no proximity was observed between any of the points regardless of the herd, with the greatest distance observed between H2BALF and H1BALF (Figure 2).

#### Weighted Unifrac distance

**Figure 3:**
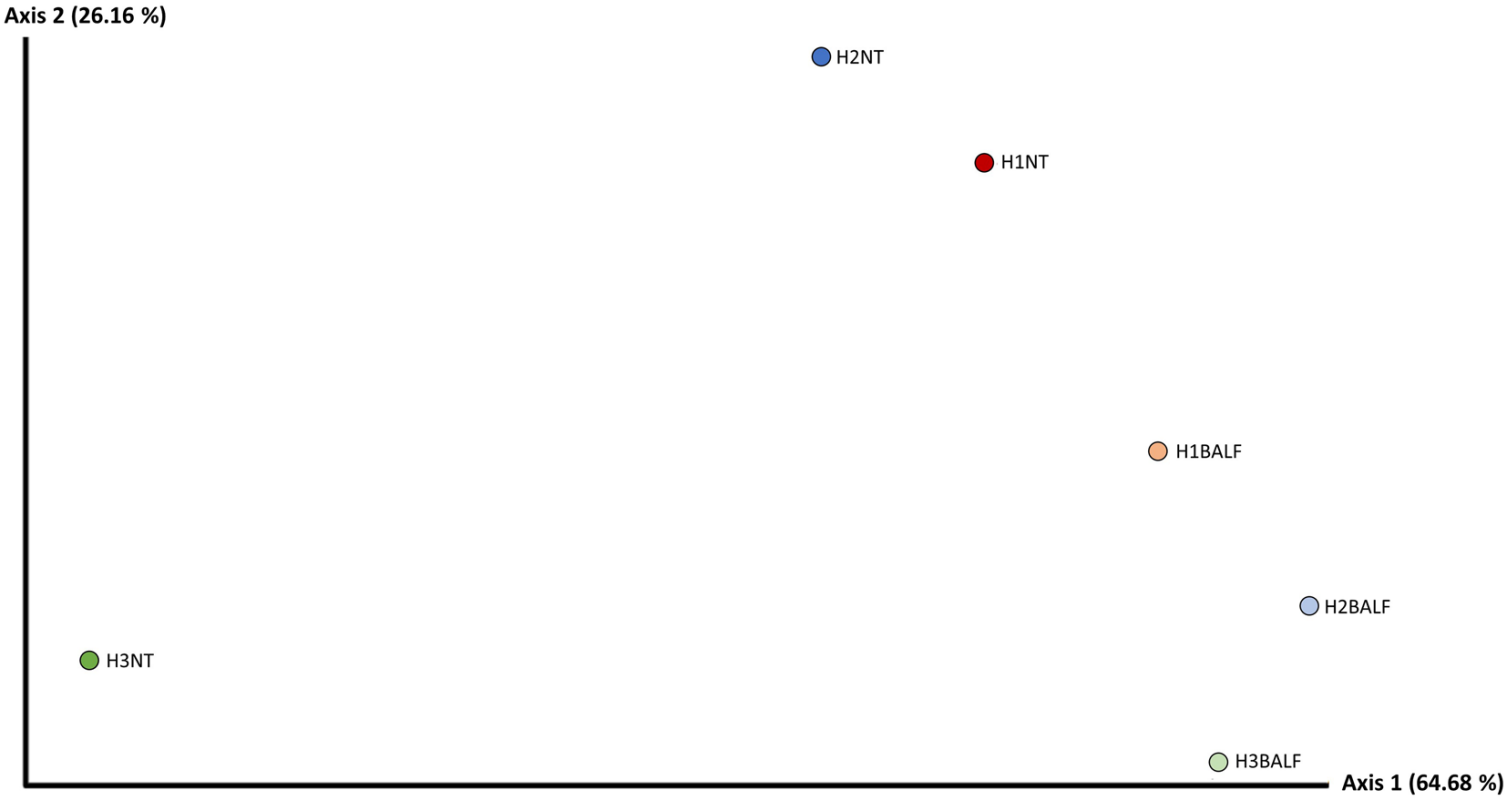
**Weighted Unifrac distance Pcoa diagram based on NT and BALF pooled samples of the *M. hyopneumoniae*-infected (H2 and H3) and** ***M. hyopneumoniae*-free (H1) herds.**

In the weighted Unifrac distance PCoa, axis 1 (64.68%) explains a high percentage of variation in relation to the other axes. The samples of H2BALF, H3BALF, and H1BALF were close to each other and relatively far from H1NT and H2NT samples, and even further away from H3NT, possibly due to the low abundance in the sample. NT and BALF samples from uninfected animals were closer to each other when compared to corresponding samples in herds 2 and 3 (H2 and H3), as observed in Figure 3.

### Taxonomic analysis

**Figure 4:**
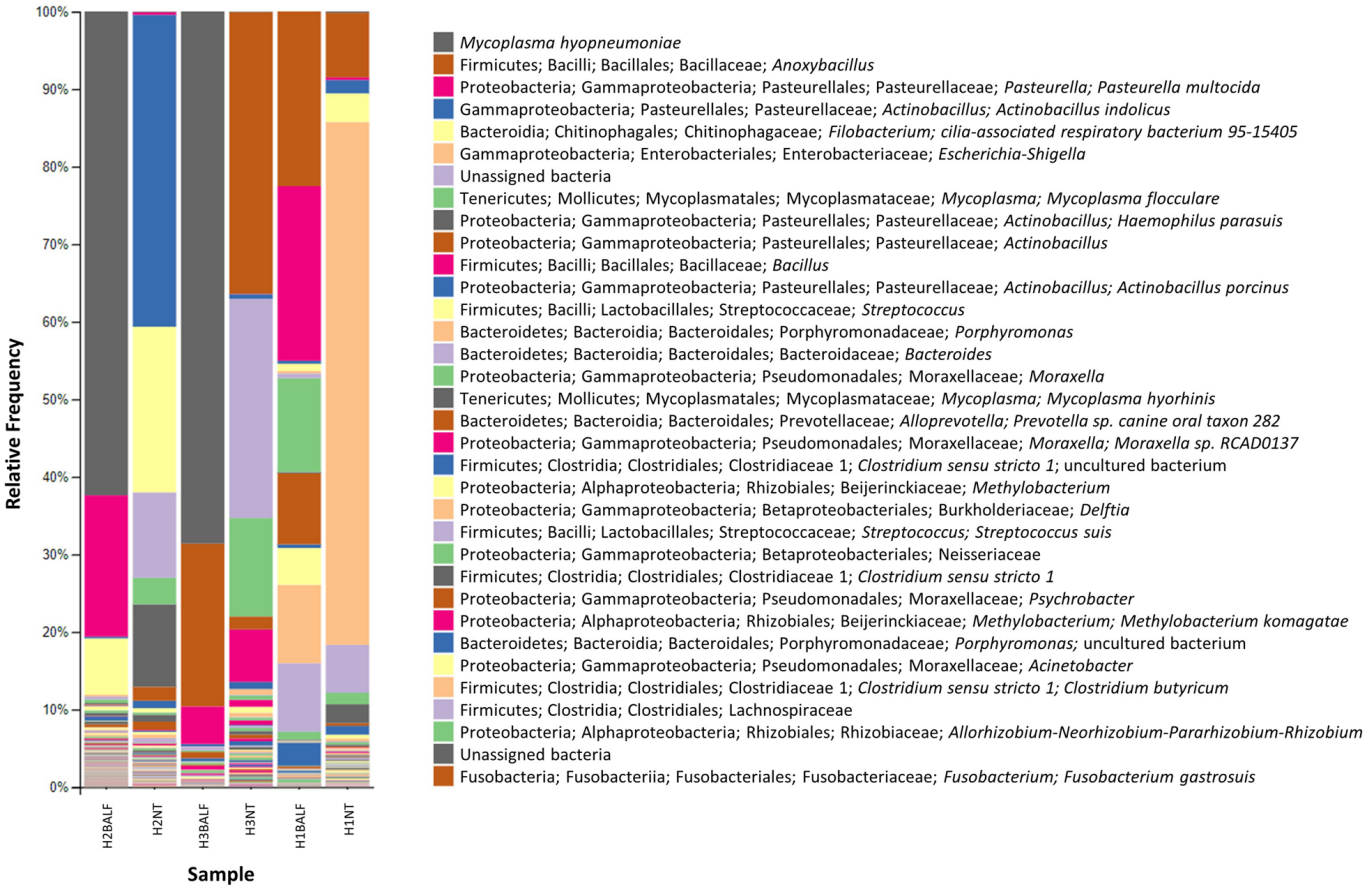
**Taxonomic analysis based on NT and BALF pooled samples of the *M. hyopneumoniae*-infected (H2 and H3) and* M. hyopneumoniae*-free (H1) herds.**

The taxonomic analysis showed a high relative frequency of *M. hyopneumoniae* in samples H2BALF and H3BALF (62.39% and 68.61%). *Mycoplasma hyopneumoniae* was also detected in H1NT, H2NT, and H3NT, with the respective frequency of 0.15%, 0.09%, and 0.08%. Considering that all BALF samples were negative on the qPCR, it is possible that the occurrence of *M. hyopneumoniae* in H1NT is related to contamination during slaughter. In samples H2BALF, H3BALF, and H1BALF, relative frequencies of *P. multocida* were 18.21%, 4.83%, and 22.5%, respectively. *Anoxybacillus* was recorded in BALF and NT samples only in H3 and H1, ranging from 8.36% (H3BALF) to 36.36% (H3NT). Following the order of relative frequency, *Actinobacillus indolicus* appears in all samples, but only in H2NT, a high relative frequency of 40.2% was recorded, and in the others, the values (H3: 0.58% and H1: 1.74%) were not higher than 2%. Cilia-associated respiratory bacterium 95-15405 was identified in samples from H1 and H2, with the highest relative frequencies observed in H2NT and H2BALF, 21.36% and 7.28%, while in samples from H1 the values were not higher than 4% (H1NT: 3.72% and H1BALF: 0.94%). Unexpectedly, a high relative frequency of 67.40% of *Escherichia/Shigella* was identified in the H1NT sample while samples H1BALF, H2BALF, and H3BALF showed relative frequencies close to 0.3%. *Mycoplasma flocculare* was identified in all analyzed samples, and among the BALF samples the highest value was for H1BALF (12.09%) and in lower prevalence in the samples H2BALF (0.5%) and H3BALF (0.18%). On the other hand, NT samples showed a higher prevalence of *M. flocculare* in H2NT (3.41%) and H3NT (12.68%), while in H1NT the percentage was the lowest, 1.48%. Main results are shown in Figure 4 and complete results are available in Additional file 4.

## Discussion

*Mycoplasma hyopneumoniae* is described as the primary agent in respiratory diseases in pigs, present worldwide, and directly related to the occurrence of pulmonary consolidation lesions in pigs [20]. The present comparative study, carried out with nasal turbinate (NT) and bronchoalveolar lavage fluid (BALF) samples of animals infected and not infected by the pathogen, indicated a predominance of *M. hyopneumoniae* and low species diversity in alpha and beta diversity metric analyses, in addition to significant correlations between *M. hyopneumoniae* DNA quantification and macroscopic lung consolidated lesions (MLCL).

The qPCR results showed a high occurrence of *M. hyopneumoniae* in H2 and H3 and high correlation with MLCL, which was also recently reported by Ferreira et al. [5]. In addition, the authors indicated that the greater the extent of MLCL, the greater the concentration of *M. hyopneumoniae* and, interestingly, the lower the concentration of *M. flocculare* in the lungs. Similarly, our results indicated that *M. hyopneumoniae* concentration in BALF is negatively correlated to *M. flocculare* concentration in BALF. Therefore, since these two *Mycoplasma* species share most of the surface proteins [21] and are closely related in phylogenetic analyses, it is possible that, during infection, antibodies against *M. hyopneumoniae* cross-react with *M. flocculare* antigens, which could reduce the number of microorganisms. Considering its lower capacity of evading the immune system, *M. flocculare* would be more susceptible to be eliminated in case of co-infections with *M. hyopneumoniae* [5].

Not surprisingly, MLCL occurrence was higher in the infected herds than in the *M. hyopneumoniae*-free herd. The lesions were more severe in H2 than in H3, although *M. hyopneumoniae* relative frequency was higher in H3. However, *P. multocida* relative frequency was also higher in H2, which could have worsened the lung lesions in H2, as reported previously [22–24]. *P. multocida* is a secondary pathogen that in combined infections with *M. hyopneumoniae* results in more severe pneumonia and clinical disease [1, 20, 23, 25, 26]. Besides, MLCL values in H1 were significantly lower than H2 and H3 even though the relative frequency of *P. multocida* was higher. Although our results suggest that *M. hyopneumoniae*-like lung lesions were worsened when *P. multocida* co-infection occurred, co-infections by other respiratory pathogens such as Influenza and PCV2 cannot be excluded as they were not assessed in this study.

Regarding the animals infected by *M. hyopneumoniae*, a predominance of the species in high relative frequency in BALF samples was shown, as well as lower numerical values in the diversity and uniformity metric, measured by the Pielou and Simpson analyses. Numerically, the H1BALF sample showed better results considering uniformity, according to Pielou and Simpson metrics, although Faith and OTUs values were low, possibly due to the low sequencing throughput (181 176 reads). Accordingly, previous studies have shown that animals infected with *M. hyopneumoniae* have a predominance of the species over other species, reducing bacterial diversity in the lung [9, 27]. It is known that *M. hyopneumoniae* stimulates the immune system, generating the release of various pro-inflammatory cytokines [28–31], induces apoptosis [32], nitric oxide stimulation [33], which promote lung damage in infected individuals. Likewise, lung damage was also observed in the present study, as animals positive for *M. hyopneumoniae* had a higher degree of pulmonary involvement. Therefore, it is possible that the indirect effects on lung injuries could be associated to the reduction in the diversity of local microorganisms, either pathogenic or commensal.

Microbiome studies have demonstrated the beneficial effect of the diversity and uniformity of bacterial species, as well as the protective benefits the presence of certain species may have for the host [34]. In our study, lower diversity values were observed in BALF samples of *M. hyopneumoniae*-infected animals, which may be an indicator of the lung’s health since higher diversity was observed in the non-infected animals (H1BALF). Other studies have shown that bacterial diversity helps to maintain balance by preventing the spread of pathogens and susceptibility to chronic diseases [35, 36]. In healthy and diverse microbiota, pathogenic bacteria are less likely to prevail due to competition for space, nutrients, and host receptors [37].

BALF samples from *M. hyopneumoniae*-infected pigs showed similarities in relation to the high abundance of *M. hyopneumoniae*, and the predominance of *P. multocida* among the main species of bacteria identified. Moreover, it seems that other bacterial genera are present in both infected and *M. hyopneumoniae*-free BALF samples due to the similarity observed in the PCoa metrics by Bray Curtis. *Mycoplasma hyopneumoniae* evasion characteristics and its ability to induce local inflammation are strategies that allow the pathogen to be present at higher relative frequencies [1, 20, 38]. Additionally, it seems to be the predominant species in the lungs of infected animals, which could indicate a way to perpetuate the infection and reduce competition for adhesion sites. However, additional studies are needed to better evaluate this hypothesis.

In contrast to the BALF results, low relative frequencies of *M. hyopneumoniae* were detected in NT samples, as well as better results from the point of view of alpha diversity. This corroborates literature data that state that *M. hyopneumoniae* presents tropism for the lower parts of the respiratory tract of pigs [39, 40]. Surprisingly, *M. hyopneumoniae* was identified, in a very low relative frequency, in the H1NT sample, but not in H1BALF or the individual qPCR results. Even though *M. hyopneumoniae* was identified in NT sample from a negative farm, it is possible that cross contamination occurred during the slaughter process, as reported by Marois et al. [41], where *M. hyopneumoniae* DNA was found in nasal and tracheal swabs of SPF pigs that had no contact with other pigs at slaughter. In that case, the scalding water could have been a source of contamination since *M. hyopneumoniae* DNA was detected even before the onset of slaughter [41]. The authors also reported a higher occurrence of *M. hyopneumoniae* DNA in SPF pigs that were in the rest area for 4 h before slaughter. Therefore, considering the similar conditions in our study, it is possible that infection occurred just before slaughter in the rest pen, when *M. hyopneumoniae*-infected and non-infected pigs were allocated close together, or during the slaughter process.

Qualitative measures of dissimilarity, Jaccard, and Unweighted unifrac, observed in the PCoa diagrams indicated that for most of the samples dissimilarity was observed, regardless of infection status by *M. hyopneumoniae* and sample type, with the exception of H2NT and H3NT samples that were relatively close to each other, indicating similarity between the compositions of microbiota in these samples. Although most of the points are equidistant, it was possible to notice in the PCoa diagram based on the Unweighted unifrac distance, that the distance was greater between BALF and NT samples. The greatest dissimilarity was registered between H1BALF and H2BALF, indicating qualitative differences between bacteria that make up both samples. In addition, the composition and diversity of the microbiota also seem to be closely related to environmental conditions. Differences in the swine rearing environment have been shown to have an influence on the animals’ respiratory and intestinal microbiota [42, 43]. Therefore, when establishing comparisons between animals from different properties, it is necessary to take into account the fact that they may have been colonized by different bacteria, which could result in lack of beta similarity between samples.

Furthermore, the microbial composition of pig’s microbiota is dynamic and is related to several factors as environment, production system, diet, antimicrobial use and others [10]. The impact of antimicrobial use on the microbiota of pigs depends on the chemical structure, route of administration, dose, and duration of treatment [44]. Zeineldin et al. [45] reported that each of the different antimicrobials tested in their study had a distinct impact on the nasal microbiota community structure, and that short-term antimicrobial administration could alter the nasal microbiota diversity and richness. Additionally, Correa-Fiz et al. [10] noted a lower relative abundance of potentially pathogenic bacteria in the nasal microbiota, while beneficial bacteria were more abundant in the farms where no perinatal antimicrobial treatment was used. Nevertheless, considering that the respiratory microbiota normally reaches stability 2–3 weeks after weaning and that the effect of antimicrobial treatment may differ in adult animals with an established nasal microbiota, further studies aiming at exploring the effects of antimicrobial use on the respiratory microbiota of healthy and clinically affected pigs should be conducted [45].

In general, the abundance of microorganisms in the lung microbiota was affected by *M. hyopneumoniae* infection in BALF pools, but not in NT samples. The beta diversity analyses indicated differences between the abundance of microorganisms between the BALF samples when comparing *M. hyopneumoniae*-infected and non-infected herds, which is confirmed by observing the taxonomic analysis. Therefore, it is possible to observe different bacteria with approximate relative frequencies, which makes it more difficult to determine a single predominant species. On the other hand, samples H2BALF and H3BALF, demonstrated the predominance of *M. hyopneumoniae*. Nonetheless, further studies comparing the impact of different *M. hyopneumoniae* strains on the lung microbiota of infected animals are needed, as little is known about the virulence of the strains circulating in Brazil [46, 47].

In our study, sequencing pooled samples was a determining factor in making it possible to have an overview of the respiratory microbiota from the three groups, especially when considering the costs of the analyses. As shown by Ray et al. [48], pooling samples can cut the costs tenfold. In addition, pooling samples before DNA amplification to estimate community level diversity is a viable and valuable measure to consider in population-level studies [48]. However, pooling the individual samples to one sample per farm has the disadvantage that no quantitative comparisons and statistical analyses can be made due to the small number of observations.

Although our results are important and can be useful to help understanding the respiratory microbiota modulation in *M. hyopneumoniae*-infected pigs, some experimental limitations should be addressed and acknowledged, and the results reported here should be interpreted with caution. For instance, although NT samples showed higher diversity, the detection of *M. hyopneumoniae* was lower for these samples. Therefore, considering our results, the use of BALF samples seems more valuable when the aim is to assess possible associations between *M. hyopneumoniae* infection and the respective influence on the microbiota. Additionally, since sampling collection took place in the slaughterhouse, some sources of contamination such as the scalding water and the sawing machine were controlled only by the federal inspection system, and not by the researchers. Thus, possible contamination in the upper respiratory tract samples needs to be taken into account when interpreting the respective results. Even though internal negative controls were included and tested negative by qPCR for *Mycoplasma* [5], these samples were not sequenced and therefore, a possible background contamination in the microbiome analysis cannot be ruled out. However, it is important to mention that all samples were processed in the same way, using the same procedures and using reagents from the same batch.

Overall, it has been observed that the presence or absence of a particular pathogen can affect directly or indirectly, not only promoting the development of lesions but also modulating the diversity, uniformity, and abundance of other bacterial populations in the respiratory tract of pigs. However, since the animals were assessed at slaughter, the results are not necessarily applicable for the situation during fattening period and earlier stages. Therefore, our results partially demonstrate the impact of *M. hyopneumoniae* in the lungs of infected pigs at slaughter, and further studies are needed to investigate the significance of *M. hyopneumoniae* infection in the microbiome of diseased pigs in comparison to healthy animals.

The presence of *M. hyopneumoniae* in BALF of slaughter pigs seems to modulate the microbiota, which could facilitate dysbiosis and proliferation of other pathogens. *Mycoplasma hyopneumoniae* quantification was strongly correlated with macroscopic lung consolidated lesions, meaning that the severity of the lesions increases when there is a higher number of microorganisms.

## Supplementary Information

**Additional file 1. Detailed vaccination protocol for farms H1, H2, and H3.**

**Additional file 2. Parameters for qPCR assays based on the gene fragments of *M. hyopneumoniae* (p102), *M. hyorhinis* (p37), and *M. flocculare* (fruA).** qPCR assays’ information for all plate runs, including efficiency (E), r2, slope, and Y-intercept for the three pathogens tested: *M. hyopneumoniae* (FAM), *M. hyorhinis* (Texas Red), and *M. flocculare* (Cy5).

**Additional file 3. Detailed information on the individual samples tested by multiplex-qPCR.** Individual qPCR results of BALF and NT samples tested against *M. hyopneumoniae*, *M. hyorhinis*, and *M. flocculare*.

**Additional file 4. Detailed information on the taxonomic analysis with relative frequencies of each bacterial genera from pooled nasal tubinate (NT) and bronchoalveolar fluid (BALF) samples.** Taxonomic analyses of pooled BALF and NT samples of *M. hyopneumoniae-*infected and non-infected pigs. The following results consider a taxonomic level 7 (species).

## Data Availability

All data generated or analyzed during this study are included in this article and its Additional files, but additional information can be provided on reasonable request.
