# Supplementary material for: Influence of Mycoplasma hyopneumoniae natural infection on the respiratory microbiome diversity of finishing pigs
Source: Vet Res. 2022 Mar 18;53:20. doi: 10.1186/s13567-022-01038-9 (PMC8932171; doi:10.1186/s13567-022-01038-9)
Supplement: Supplementary file 1 — Additional file 1. Detailed vaccination protocol for farms H1, H2, and H3. [file 13567_2022_1038_MOESM1_ESM.docx]

**Additional file 1**. **Detailed vaccination protocol for farms H1, H2, and H3.**

|  | **Piglets in nursery** | |  | **Sows** |
| --- | --- | --- | --- | --- |
|  | **1^st^ dose (21 days)** | **2^nd^ dose (42 days)** |  | **5 to 10 days post-partum** |
| ***M. hyopneumoniae**** | X | X |  |  |
| ***P. multocida*** | X | X |  |  |
| ***G. parasuis*** | X | X |  |  |
| **Influenza** | X | X |  |  |
| **Porcine Circovirus** | X | X |  |  |
| **Parvovirus** |  |  |  | X |
| ***Leptospira* sp.** |  |  |  | X |
| ***E. rhusiopathiae*** |  |  |  | X |

*Vaccination only in farms H2 and H3.
