# Supplementary material for: Influence of Mycoplasma hyopneumoniae natural infection on the respiratory microbiome diversity of finishing pigs
Source: Vet Res. 2022 Mar 18;53:20. doi: 10.1186/s13567-022-01038-9 (PMC8932171; doi:10.1186/s13567-022-01038-9)
Supplement: Supplementary file 2 — Additional file 2. Parameters for qPCR assays based on the gene fragments of M. hyopneumoniae (p102), M. hyorhinis (p37), and M. flocculare (fruA). qPCR assays’ information for all plate runs, including efficiency (E), r2, slope, and Y-intercept for the three pathogens tested: M. hyopneumoniae (FAM), M. hyorhinis (Texas Red), and M. flocculare (Cy5). [file 13567_2022_1038_MOESM2_ESM.docx]

**Additional file 2**. **Parameters for qPCR assays based on the gene fragments of *M. hyopneumoniae (p102), M. hyorhinis (p37),* and *M. flocculare (fruA).***

|  | **Fluorophore** | **Efficiency (E)** | **r^2^** | **Slope** | **Y-intercept** |
| --- | --- | --- | --- | --- | --- |
| **Plate 1** | FAM | 98.5 | 0.998 | -3.357 | 40 |
|  | Cy5 | 97.1 | 0.995 | -3.393 | 39.7 |
|  | Texas Red | 100.4 | 0.995 | -3.313 | 37.7 |
| **Plate 2** | FAM | 101 | 0.998 | -3.299 | 40.5 |
|  | Cy5 | 99.3 | 0.992 | -3.339 | 38.8 |
|  | Texas Red | 101.4 | 0.999 | -3.29 | 38.9 |
| **Plate 3** | FAM | 97.8 | 0.998 | -3.376 | 40.8 |
|  | Cy5 | 101.5 | 0.996 | -3.287 | 38.7 |
|  | Texas Red | 100.9 | 0.997 | -3.3 | 38.6 |
| **Plate 4** | FAM | 100 | 0.998 | -3.322 | 40.2 |
|  | Cy5 | 97.5 | 0.996 | -3.382 | 39.5 |
|  | Texas Red | 100.9 | 0.996 | -3.301 | 39.2 |
| **Plate 5** | FAM | 99.3 | 0.997 | -3.339 | 38.5 |
|  | Cy5 | 92.3 | 0.999 | -3.522 | 40.5 |
|  | Texas Red | 97.3 | 0.999 | -3.388 | 38.6 |
| **Plate 6** | FAM | 99.2 | 0.997 | -3.341 | 38.7 |
|  | Cy5 | 97.8 | 0.993 | -3.375 | 39.4 |
|  | Texas Red | 96.8 | 0.999 | -3.401 | 38.6 |

r^2^: determination coefficient.
