# Supplementary material for: Influence of Mycoplasma hyopneumoniae natural infection on the respiratory microbiome diversity of finishing pigs
Source: Vet Res. 2022 Mar 18;53:20. doi: 10.1186/s13567-022-01038-9 (PMC8932171; doi:10.1186/s13567-022-01038-9)
Supplement: Supplementary file 3 — Additional file 3. Detailed information on the individual samples tested by multiplex-qPCR. Individual qPCR results of BALF and NT samples tested against M. hyopneumoniae, M. hyorhinis, and M. flocculare. [file 13567_2022_1038_MOESM3_ESM.docx]

**Additional file 3 Detailed information on the individual samples tested by multiplex*-*qPCR.**

|  |  |  | **BALF samples** | | | | | | | |  | **NT samples** | | | | | | | |
| --- | --- | --- | --- | --- | --- | --- | --- | --- | --- | --- | --- | --- | --- | --- | --- | --- | --- | --- | --- |
|  |  |  | *M. hyopneumoniae* | |  | *M. flocculare* | |  | *M. hyorhinis* | |  | *M. hyopneumoniae* | |  | *M. flocculare* | |  | *M. hyorhinis* | |
|  | **Sample ID** |  | **Cq mean** | **SQ mean** |  | **Cq mean** | **SQ mean** |  | **Cq mean** | **SQ mean** |  | **Cq mean** | **SQ mean** |  | **Cq mean** | **SQ mean** |  | **CQ mean** | **Sq mean** |
| **Herd 1 (H1)** | 4.1 |  | 0.00 | 0.00E+00 |  | 20.24 | 4.34E+05 |  | * | * |  | 0.00 | 0.00E+00 |  | 25.10 | 1.79E+04 |  | 24.52 | 1.36E+04 |
|  | 4.2 |  | 0.00 | 0.00E+00 |  | 18.88 | 1.13E+06 |  | 0.00 | 0.00E+00 |  | 0.00 | 0.00E+00 |  | 25.14 | 1.73E+04 |  | 27.95 | 1.34E+03 |
|  | 4.3 |  | 0.00 | 0.00E+00 |  | 22.28 | 1.04E+05 |  | 0.00 | 0.00E+00 |  | 0.00 | 0.00E+00 |  | 25.01 | 1.92E+04 |  | 35.18 | 1.55E+01 |
|  | 4.4 |  | 0.00 | 0.00E+00 |  | 20.90 | 2.73E+05 |  | 31.95 | 1.11E+02 |  | 0.00 | 0.00E+00 |  | 25.96 | 1.01E+04 |  | 25.19 | 8.70E+03 |
|  | 4.5 |  | 0.00 | 0.00E+00 |  | 21.92 | 1.34E+05 |  | 29.03 | 7.83E+02 |  | 0.00 | 0.00E+00 |  | 24.73 | 2.32E+04 |  | 30.63 | 4.28E+02 |
|  | 4.6 |  | 0.00 | 0.00E+00 |  | 26.29 | 6.28E+03 |  | 32.78 | 5.76E+01 |  | 0.00 | 0.00E+00 |  | 28.50 | 1.80E+03 |  | 28.48 | 9.36E+02 |
|  | 4.7 |  | 0.00 | 0.00E+00 |  | 26.48 | 5.51E+03 |  | 0.00 | 0.00E+00 |  | 0.00 | 0.00E+00 |  | 28.00 | 2.47E+03 |  | 0.00 | 0.00E+00 |
|  | 4.8 |  | 0.00 | 0.00E+00 |  | 0.00 | 0.00E+00 |  | 24.60 | 1.72E+04 |  | 0.00 | 0.00E+00 |  | 27.58 | 3.29E+03 |  | 29.39 | 5.20E+02 |
|  | 4.9 |  | 0.00 | 0.00E+00 |  | 23.23 | 5.34E+04 |  | * | * |  | 0.00 | 0.00E+00 |  | 27.41 | 3.69E+03 |  | 28.36 | 1.03E+03 |
|  | 4.10 |  | 0.00 | 0.00E+00 |  | 22.08 | 1.20E+05 |  | 31.03 | 1.94E+02 |  | 0.00 | 0.00E+00 |  | 25.66 | 1.21E+04 |  | 31.62 | 1.22E+02 |
|  | 4.11 |  | 0.00 | 0.00E+00 |  | 23.35 | 4.93E+04 |  | * | * |  | 0.00 | 0.00E+00 |  | 23.69 | 4.78E+04 |  | 33.03 | 4.67E+01 |
|  | 4.12 |  | 0.00 | 0.00E+00 |  | 21.39 | 1.94E+05 |  | 28.19 | 1.40E+03 |  | 0.00 | 0.00E+00 |  | 24.96 | 1.97E+04 |  | 29.89 | 3.59E+02 |
|  | 4.13 |  | 0.00 | 0.00E+00 |  | 26.45 | 5.62E+03 |  | 26.69 | 4.01E+03 |  | 0.00 | 0.00E+00 |  | 26.66 | 6.21E+03 |  | 29.61 | 4.36E+02 |
|  | 4.14 |  | 0.00 | 0.00E+00 |  | 20.14 | 4.68E+05 |  | 31.01 | 2.22E+02 |  | 0.00 | 0.00E+00 |  | 24.14 | 3.45E+04 |  | 27.20 | 2.23E+03 |
|  | 4.15 |  | 0.00 | 0.00E+00 |  | 26.88 | 4.19E+03 |  | 0.00 | 0.00E+00 |  | 0.00 | 0.00E+00 |  | 26.53 | 6.73E+03 |  | 0.00 | 0.00E+00 |
|  | 4.16 |  | 0.00 | 0.00E+00 |  | 23.16 | 6.94E+04 |  | 0.00 | 0.00E+00 |  | 0.00 | 0.00E+00 |  | 26.39 | 7.63E+03 |  | 26.94 | 2.67E+03 |
|  | 4.17 |  | 0.00 | 0.00E+00 |  | 21.25 | 2.54E+05 |  | 0.00 | 0.00E+00 |  | 0.00 | 0.00E+00 |  | 26.82 | 5.68E+03 |  | 33.92 | 2.40E+01 |
|  | 4.18 |  | 0.00 | 0.00E+00 |  | 21.39 | 2.31E+05 |  | 0.00 | 0.00E+00 |  | 0.00 | 0.00E+00 |  | 25.26 | 1.59E+04 |  | 28.01 | 1.30E+03 |
|  | 4.19 |  | 0.00 | 0.00E+00 |  | 23.53 | 5.40E+04 |  | 0.00 | 0.00E+00 |  | 0.00 | 0.00E+00 |  | 25.78 | 1.12E+04 |  | 25.02 | 9.73E+03 |
|  | 4.20 |  | 0.00 | 0.00E+00 |  | 21.24 | 2.56E+05 |  | 28.60 | 1.61E+03 |  | 0.00 | 0.00E+00 |  | 0.00 | 0.00E+00 |  | 23.94 | 2.03E+04 |
|  | 4.21 |  | 0.00 | 0.00E+00 |  | 23.18 | 6.85E+04 |  | 0.00 | 0.00E+00 |  | 0.00 | 0.00E+00 |  | 0.00 | 0.00E+00 |  | 25.89 | 5.41E+03 |
|  | 4.22 |  | 0.00 | 0.00E+00 |  | 17.99 | 2.34E+06 |  | 36.23 | 7.85E+00 |  | 0.00 | 0.00E+00 |  | 0.00 | 0.00E+00 |  | 24.16 | 1.74E+04 |
| **Herd 2 (H2)** | 1.1 |  | 22.08 | 2.47E+05 |  | 25.61 | 9.96E+03 |  | 0.00 | 0.00E+00 |  | 0.00 | 0.00E+00 |  | 0.00 | 0.00E+00 |  | 28.51 | 9.87E+02 |
|  | 1.2 |  | 19.68 | 1.27E+06 |  | 22.15 | 1.04E+05 |  | 27.89 | 9.27E+02 |  | 32.00 | 1.62E+02 |  | 31.07 | 4.82E+02 |  | 0.00 | 0.00E+00 |
|  | 1.3 |  | 0.00 | 0.00E+00 |  | 24.24 | 2.53E+04 |  | 22.80 | 3.19E+04 |  | 28.04 | 1.41E+03 |  | 0.00 | 0.00E+00 |  | 24.69 | 1.30E+04 |
|  | 1.4 |  | 19.95 | 1.06E+06 |  | 28.31 | 1.59E+03 |  | * | * |  | 0.00 | 0.00E+00 |  | 0.00 | 0.00E+00 |  | 30.34 | 2.86E+02 |
|  | 1.5 |  | 18.26 | 3.36E+06 |  | 21.99 | 1.15E+05 |  | 0.00 | 0.00E+00 |  | * | * |  | 22.36 | 1.43E+05 |  | 33.97 | 2.30E+01 |
|  | 1.6 |  | 17.42 | 9.94E+06 |  | 20.36 | 3.34E+05 |  | * | * |  | 0.00 | 0.00E+00 |  | 24.85 | 2.80E+04 |  | 0.00 | 0.00E+00 |
|  | 1.7 |  | 24.10 | 9.40E+04 |  | 28.18 | 1.53E+03 |  | 0.00 | 0.00E+00 |  | 0.00 | 0.00E+00 |  | 30.02 | 9.51E+02 |  | 0.00 | 0.00E+00 |
|  | 1.8 |  | 16.18 | 2.36E+07 |  | 0.00 | 0.00E+00 |  | 0.00 | 0.00E+00 |  | 0.00 | 0.00E+00 |  | 25.59 | 1.76E+04 |  | 30.71 | 2.17E+02 |
|  | 1.9 |  | 19.50 | 2.33E+06 |  | 23.08 | 5.08E+04 |  | 35.61 | 1.01E+01 |  | 35.75 | 1.18E+01 |  | * | * |  | 24.45 | 1.52E+04 |
|  | 1.10 |  | 17.32 | 1.07E+07 |  | 24.24 | 2.38E+04 |  | 27.86 | 2.28E+03 |  | 0.00 | 0.00E+00 |  | 27.26 | 5.76E+03 |  | 33.46 | 6.00E+01 |
|  | 1.11 |  | 19.69 | 2.05E+06 |  | 22.97 | 5.50E+04 |  | 33.01 | 6.28E+01 |  | * | * |  | 27.03 | 4.80E+03 |  | 30.07 | 3.43E+02 |
|  | 1.12 |  | 17.15 | 1.20E+07 |  | 0.00 | 0.00E+00 |  | 0.00 | 0.00E+00 |  | 0.00 | 0.00E+00 |  | 25.06 | 2.43E+04 |  | 31.18 | 1.58E+02 |
|  | 1.13 |  | 15.25 | 4.51E+07 |  | 38.99 | 1.07E+00 |  | 0.00 | 0.00E+00 |  | 26.75 | 3.37E+03 |  | 25.03 | 2.46E+04 |  | * | * |
|  | 1.14 |  | 15.37 | 4.18E+07 |  | 18.19 | 1.49E+06 |  | 24.46 | 2.50E+04 |  | 0.00 | 0.00E+00 |  | 0.00 | 0.00E+00 |  | 22.57 | 5.48E+04 |
|  | 1.15 |  | 17.70 | 8.20E+06 |  | 30.08 | 4.16E+02 |  | 33.58 | 4.21E+01 |  | 0.00 | 0.00E+00 |  | 30.49 | 4.56E+02 |  | 0.00 | 0.00E+00 |
|  | 1.16 |  | 15.67 | 3.37E+07 |  | 12.54 | 7.31E+07 |  | 17.65 | 2.90E+06 |  | 0.00 | 0.00E+00 |  | 24.94 | 2.68E+04 |  | 0.00 | 0.00E+00 |
|  | 1.17 |  | 21.87 | 4.45E+05 |  | 39.82 | 4.97E-01 |  | 32.76 | 7.46E+01 |  | 0.00 | 0.00E+00 |  | 25.75 | 1.60E+04 |  | 33.66 | 2.96E+01 |
|  | 1.18 |  | 17.67 | 8.37E+06 |  | 23.15 | 4.87E+04 |  | 0.00 | 0.00E+00 |  | 27.30 | 2.32E+03 |  | 29.88 | 8.68E+02 |  | 0.00 | 0.00E+00 |
|  | 1.19 |  | 16.00 | 2.69E+07 |  | 29.19 | 1.12E+03 |  | 27.85 | 2.32E+03 |  | 0.00 | 0.00E+00 |  | 0.00 | 0.00E+00 |  | 29.96 | 3.77E+02 |
|  | 1,20 |  | 23.96 | 1.03E+05 |  | 25.08 | 1.29E+04 |  | 32.67 | 7.95E+01 |  | 26.43 | 4.20E+03 |  | 0.00 | 0.00E+00 |  | 33.73 | 2.87E+01 |
|  | 1.21 |  | 21.37 | 6.30E+05 |  | 26.85 | 4.54E+03 |  | 32.01 | 1.26E+02 |  | 0.00 | 0.00E+00 |  | 26.17 | 1.17E+04 |  | 31.51 | 1.21E+02 |
|  | 1.22 |  | 21.82 | 4.61E+05 |  | 28.54 | 1.18E+03 |  | 0.00 | 0.00E+00 |  | 0.00 | 0.00E+00 |  | 0.00 | 0.00E+00 |  | 28.68 | 8.78E+02 |
| **Herd 3 (H3)** | 3.1 |  | 31.21 | 6.59E+02 |  | 29.85 | 4.98E+02 |  | 0.00 | 0.00E+00 |  | 0.00 | 0.00E+00 |  | 25.57 | 1.74E+04 |  | 0.00 | 0.00E+00 |
|  | 3.2 |  | 32.34 | 2.98E+02 |  | 26.10 | 6.39E+03 |  | 28.45 | 1.51E+03 |  | 0.00 | 0.00E+00 |  | 24.37 | 3.80E+04 |  | 0.00 | 0.00E+00 |
|  | 3.3 |  | 21.25 | 6.88E+05 |  | 26.22 | 5.85E+03 |  | * | * |  | 0.00 | 0.00E+00 |  | 29.30 | 1.56E+03 |  | 0.00 | 0.00E+00 |
|  | 3.4 |  | 24.02 | 9.92E+04 |  | 0.00 | 0.00E+00 |  | 0.00 | 0.00E+00 |  | 0.00 | 0.00E+00 |  | 31.05 | 4.89E+02 |  | 34.34 | 1.87E+01 |
|  | 3.5 |  | 27.25 | 1.04E+04 |  | * | * |  | 34.31 | 2.51E+01 |  | 0.00 | 0.00E+00 |  | 30.15 | 8.90E+02 |  | 0.00 | 0.00E+00 |
|  | 3.6 |  | 23.39 | 1.54E+05 |  | 28.08 | 1.63E+03 |  | 0.00 | 0.00E+00 |  | 0.00 | 0.00E+00 |  | 30.69 | 6.30E+02 |  | 0.00 | 0.00E+00 |
|  | 3.7 |  | 26.81 | 1.42E+04 |  | 28.83 | 9.67E+02 |  | 0.00 | 0.00E+00 |  | 0.00 | 0.00E+00 |  | 0.00 | 0.00E+00 |  | 0.00 | 0.00E+00 |
|  | 3.8 |  | 18.60 | 4.39E+06 |  | 0.00 | 0.00E+00 |  | * | * |  | 0.00 | 0.00E+00 |  | 29.88 | 1.06E+03 |  | 0.00 | 0.00E+00 |
|  | 3.9 |  | 20.18 | 1.45E+06 |  | 0.00 | 0.00E+00 |  | 0.00 | 0.00E+00 |  | 0.00 | 0.00E+00 |  | 26.75 | 8.02E+03 |  | 0.00 | 0.00E+00 |
|  | 3.10 |  | 23.79 | 1.16E+05 |  | 26.24 | 5.89E+03 |  | 0.00 | 0.00E+00 |  | 0.00 | 0.00E+00 |  | 28.36 | 1.94E+03 |  | 0.00 | 0.00E+00 |
|  | 3.11 |  | 26.66 | 1.58E+04 |  | 26.87 | 4.18E+03 |  | 0.00 | 0.00E+00 |  | 0.00 | 0.00E+00 |  | 29.45 | 1.40E+03 |  | 0.00 | 0.00E+00 |
|  | 3.12 |  | 24.00 | 9.69E+04 |  | 0.00 | 0.00E+00 |  | 0.00 | 0.00E+00 |  | 0.00 | 0.00E+00 |  | 26.23 | 1.14E+04 |  | 0.00 | 0.00E+00 |
|  | 3.13 |  | 27.07 | 1.19E+04 |  | 0.00 | 0.00E+00 |  | 0.00 | 0.00E+00 |  | 0.00 | 0.00E+00 |  | 0.00 | 0.00E+00 |  | 0.00 | 0.00E+00 |
|  | 3.14 |  | 20.33 | 1.19E+06 |  | 29.84 | 5.34E+02 |  | 0.00 | 0.00E+00 |  | 29.60 | 4.73E+02 |  | 30.78 | 3.91E+02 |  | 0.00 | 0.00E+00 |
|  | 3.15 |  | 19.57 | 1.99E+06 |  | 0.00 | 0.00E+00 |  | 0.00 | 0.00E+00 |  | 27.57 | 1.91E+03 |  | 24.97 | 2.58E+04 |  | 0.00 | 0.00E+00 |
|  | 3.16 |  | 22.78 | 2.23E+05 |  | 0.00 | 0.00E+00 |  | 0.00 | 0.00E+00 |  | 0.00 | 0.00E+00 |  | 28.30 | 3.00E+03 |  | 0.00 | 0.00E+00 |
|  | 3.17 |  | 19.11 | 2.72E+06 |  | 0.00 | 0.00E+00 |  | 0.00 | 0.00E+00 |  | 30.00 | 3.67E+02 |  | 29.43 | 1.40E+03 |  | 0.00 | 0.00E+00 |
|  | 3.18 |  | 22.93 | 2.01E+05 |  | 31.78 | 1.35E+02 |  | 33.11 | 4.67E+01 |  | 0.00 | 0.00E+00 |  | 29.39 | 9.54E+02 |  | * | * |
|  | 3.19 |  | 18.47 | 4.20E+06 |  | 0.00 | 0.00E+00 |  | 35.15 | 1.11E+01 |  | 0.00 | 0.00E+00 |  | 28.23 | 2.11E+03 |  | 0.00 | 0.00E+00 |
|  | 3.20 |  | * | * |  | 24.78 | 1.81E+04 |  | 28.84 | 8.93E+02 |  | 0.00 | 0.00E+00 |  | 27.96 | 2.54E+03 |  | 0.00 | 0.00E+00 |
|  | 3.21 |  | 22.12 | 3.50E+05 |  | 34.86 | 2.45E+01 |  | 0.00 | 0.00E+00 |  | 0.00 | 0.00E+00 |  | 25.81 | 1.09E+04 |  | 0.00 | 0.00E+00 |
|  | 3.22 |  | 16.67 | 1.43E+07 |  | 0.00 | 0.00E+00 |  | 27.99 | 1.61E+03 |  | 35.56 | 9.51E+00 |  | 33.56 | 5.52E+01 |  | 35.43 | 9.83E+00 |

*Cq and SQ ranges could not be defined due to the Monte Carlo effect.

Cq = quantification cycle value; SQ = starting quantity (copies/µL)
