# Supplementary material for: Influence of Mycoplasma hyopneumoniae natural infection on the respiratory microbiome diversity of finishing pigs
Source: Vet Res. 2022 Mar 18;53:20. doi: 10.1186/s13567-022-01038-9 (PMC8932171; doi:10.1186/s13567-022-01038-9)
Supplement: Supplementary file 4 — Additional file 4. Detailed information on the taxonomic analysis with relative frequencies of each bacterial genera from pooled nasal tubinate (NT) and bronchoalveolar fluid (BALF) samples. Taxonomic analyses of pooled BALF and NT samples of M. hyopneumoniae-infected and non-infected pigs. The following results consider a taxonomic level 7 (species). [file 13567_2022_1038_MOESM4_ESM.pdf]

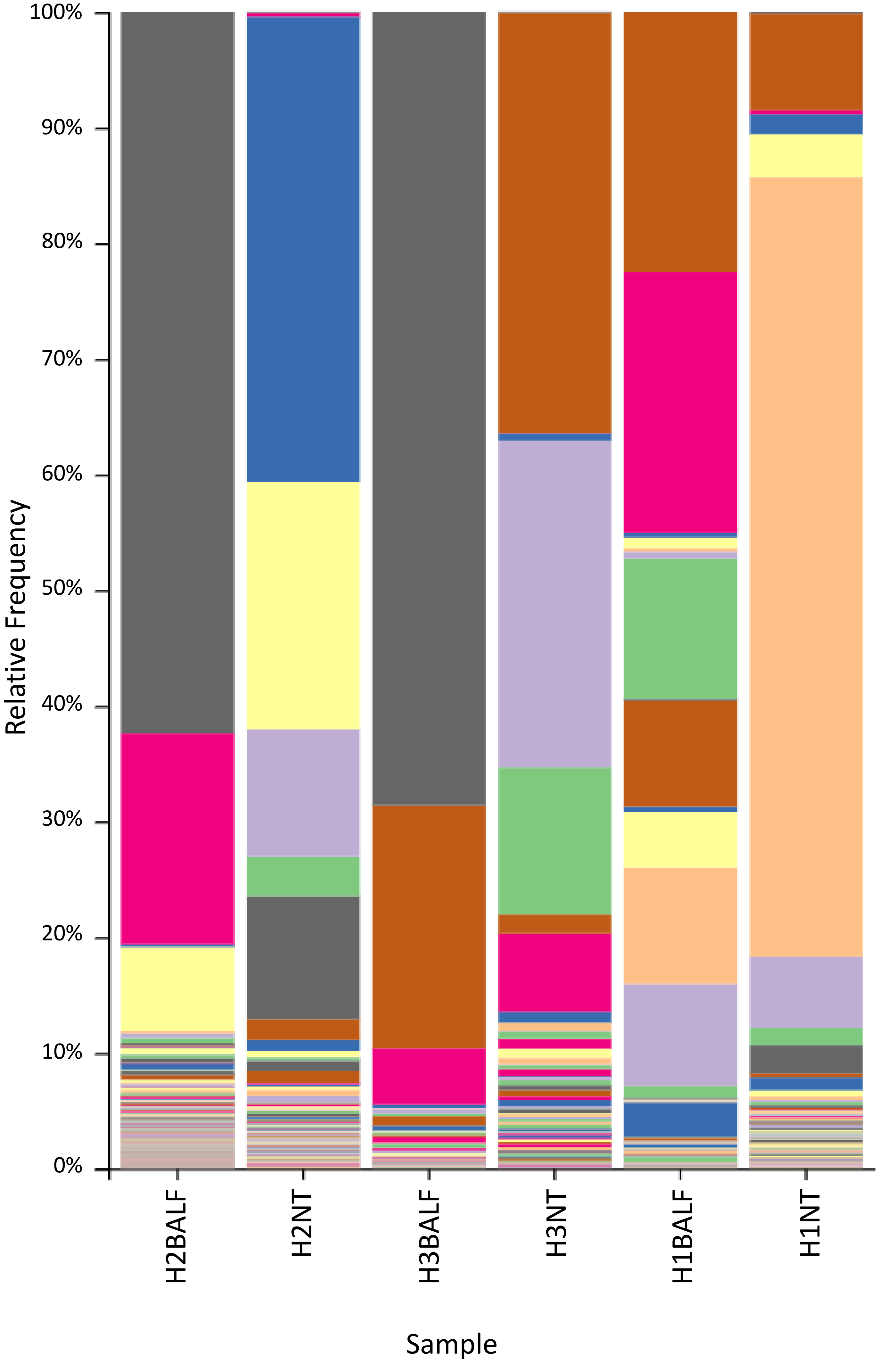

100%
90%
80%
70%
60%
50%
40%
30%
20%
10%
0%
Relative Frequency
H2BALF
H2NT
H3BALF
H3NT
H1BALF
H1NT
Sample

D_0__Bacteria;D_1__Tenericutes;D_2__Mollicutes;D_3__Mycoplasmatales;D_4__Mycoplasmataceae;D_5__Mycoplasma;D_6__Mycoplasma hyopneumoniae 232
D_0__Bacteria;D_1__Firmicutes;D_2__Bacilli;D_3__Bacillales;D_4__Bacillaceae;D_5__Anoxybacillus;__
D_0__Bacteria;D_1__Proteobacteria;D_2__Gammaproteobacteria;D_3__Pasteurellales;D_4__Pasteurellaceae;D_5__Pasteurella;D_6__Pasteurella multocida
D_0__Bacteria;D_1__Proteobacteria;D_2__Gammaproteobacteria;D_3__Pasteurellales;D_4__Pasteurellaceae;D_5__Actinobacillus;D_6__Actinobacillus indolicus
D_0__Bacteria;D_1__Bacteroidetes;D_2__Bacteroidia;D_3__Chitinophagales;D_4__Chitinophagaceae;D_5__Filobacterium;D_6__cilia-associated respiratory bacterium 95-15405
D_0__Bacteria;D_1__Proteobacteria;D_2__Gammaproteobacteria;D_3__Enterobacteriales;D_4__Enterobacteriaceae;D_5__Escherichia-Shigella;__
D_0__Bacteria;__;__;__;__;__;__
D_0__Bacteria;D_1__Tenericutes;D_2__Mollicutes;D_3__Mycoplasmatales;D_4__Mycoplasmataceae;D_5__Mycoplasma;D_6__Mycoplasma flocculare
D_0__Bacteria;D_1__Proteobacteria;D_2__Gammaproteobacteria;D_3__Pasteurellales;D_4__Pasteurellaceae;D_5__Actinobacillus;D_6__[Haemophilus] parasuis
D_0__Bacteria;D_1__Proteobacteria;D_2__Gammaproteobacteria;D_3__Pasteurellales;D_4__Pasteurellaceae;D_5__Actinobacillus;__
D_0__Bacteria;D_1__Firmicutes;D_2__Bacilli;D_3__Bacillales;D_4__Bacillaceae;D_5__Bacillus;__
D_0__Bacteria;D_1__Proteobacteria;D_2__Gammaproteobacteria;D_3__Pasteurellales;D_4__Pasteurellaceae;D_5__Actinobacillus;D_6__Actinobacillus porcinus
D_0__Bacteria;D_1__Firmicutes;D_2__Bacilli;D_3__Lactobacillales;D_4__Streptococcaceae;D_5__Streptococcus;__
D_0__Bacteria;D_1__Bacteroidetes;D_2__Bacteroidia;D_3__Bacteroidales;D_4__Porphyromonadaceae;D_5__Porphyromonas;__
D_0__Bacteria;D_1__Bacteroidetes;D_2__Bacteroidia;D_3__Bacteroidales;D_4__Bacteroidaceae;D_5__Bacteroides;__
D_0__Bacteria;D_1__Proteobacteria;D_2__Gammaproteobacteria;D_3__Pseudomonadales;D_4__Moraxellaceae;D_5__Moraxella;__
D_0__Bacteria;D_1__Tenericutes;D_2__Mollicutes;D_3__Mycoplasmatales;D_4__Mycoplasmataceae;D_5__Mycoplasma;D_6__Mycoplasma hyorhinis
D_0__Bacteria;D_1__Bacteroidetes;D_2__Bacteroidia;D_3__Bacteroidales;D_4__Prevotellaceae;D_5__Alloprevotella;D_6__Prevotella sp. canine oral taxon 282
D_0__Bacteria;D_1__Proteobacteria;D_2__Gammaproteobacteria;D_3__Pseudomonadales;D_4__Moraxellaceae;D_5__Moraxella;D_6__Moraxella sp. RCAD0137
D_0__Bacteria;D_1__Firmicutes;D_2__Clostridia;D_3__Clostridiales;D_4__Clostridiaceae 1;D_5__Clostridium sensu stricto 1;D_6__uncultured bacterium
D_0__Bacteria;D_1__Proteobacteria;D_2__Alphaproteobacteria;D_3__Rhizobiales;D_4__Beijerinckiaceae;D_5__Methylobacterium;__
D_0__Bacteria;D_1__Proteobacteria;D_2__Gammaproteobacteria;D_3__Betaproteobacteriales;D_4__Burkholderiaceae;D_5__Delftia;__
D_0__Bacteria;D_1__Firmicutes;D_2__Bacilli;D_3__Lactobacillales;D_4__Streptococcaceae;D_5__Streptococcus;D_6__Streptococcus suis
D_0__Bacteria;D_1__Proteobacteria;D_2__Gammaproteobacteria;D_3__Betaproteobacteriales;D_4__Neisseriaceae;__;__
D_0__Bacteria;D_1__Firmicutes;D_2__Clostridia;D_3__Clostridiales;D_4__Clostridiaceae 1;D_5__Clostridium sensu stricto 1;__
D_0__Bacteria;D_1__Proteobacteria;D_2__Gammaproteobacteria;D_3__Pseudomonadales;D_4__Moraxellaceae;D_5__Psychrobacter;__
D_0__Bacteria;D_1__Proteobacteria;D_2__Alphaproteobacteria;D_3__Rhizobiales;D_4__Beijerinckiaceae;D_5__Methylobacterium;D_6__Methylobacterium komagatae
D_0__Bacteria;D_1__Bacteroidetes;D_2__Bacteroidia;D_3__Bacteroidales;D_4__Porphyromonadaceae;D_5__Porphyromonas;D_6__uncultured bacterium
D_0__Bacteria;D_1__Proteobacteria;D_2__Gammaproteobacteria;D_3__Pseudomonadales;D_4__Moraxellaceae;D_5__Acinetobacter;__
D_0__Bacteria;D_1__Firmicutes;D_2__Clostridia;D_3__Clostridiales;D_4__Clostridiaceae 1;D_5__Clostridium sensu stricto 1;D_6__Clostridium butyricum
D_0__Bacteria;D_1__Firmicutes;D_2__Clostridia;D_3__Clostridiales;D_4__Lachnospiraceae;__;__
D_0__Bacteria;D_1__Proteobacteria;D_2__Alphaproteobacteria;D_3__Rhizobiales;D_4__Rhizobiaceae;D_5__Allorhizobium-Neorhizobium-Pararhizobium-Rhizobium;__
Unassigned;__;__;__;__;__;__
D_0__Bacteria;D_1__Fusobacteria;D_2__Fusobacteriia;D_3__Fusobacteriales;D_4__Fusobacteriaceae;D_5__Fusobacterium;D_6__Fusobacterium gastrosuis
D_0__Bacteria;D_1__Proteobacteria;D_2__Gammaproteobacteria;D_3__Pseudomonadales;D_4__Moraxellaceae;D_5__Moraxella;D_6__uncultured bacterium
D_0__Bacteria;D_1__Proteobacteria;D_2__Gammaproteobacteria;D_3__Xanthomonadales;D_4__Xanthomonadaceae;D_5__Stenotrophomonas;__
D_0__Bacteria;D_1__Bacteroidetes;D_2__Bacteroidia;D_3__Bacteroidales;D_4__Muribaculaceae;D_5__uncultured Porphyromonadaceae bacterium;D_6__uncultured Porphyromonadaceae bacterium
D_0__Bacteria;D_1__Firmicutes;D_2__Clostridia;D_3__Clostridiales;D_4__Peptostreptococcaceae;D_5__Terrisporobacter;D_6__uncultured bacterium
D_0__Bacteria;D_1__Firmicutes;D_2__Clostridia;D_3__Clostridiales;D_4__Ruminococcaceae;D_5__Fastidiosipila;D_6__Clostridiales bacterium canine oral taxon 216
D_0__Bacteria;D_1__Firmicutes;D_2__Clostridia;D_3__Clostridiales;D_4__Ruminococcaceae;D_5__Ruminococcaceae UCG-005;__
D_0__Bacteria;D_1__Proteobacteria;D_2__Alphaproteobacteria;D_3__Rhizobiales;D_4__Rhizobiaceae;D_5__Ochrobactrum;__
D_0__Bacteria;D_1__Bacteroidetes;D_2__Bacteroidia;D_3__Bacteroidales;D_4__Rikenellaceae;D_5__Rikenellaceae RC9 gut group;D_6__uncultured bacterium
D_0__Bacteria;D_1__Firmicutes;D_2__Clostridia;D_3__Clostridiales;D_4__Lachnospiraceae;D_5__Lachnospiraceae XPB1014 group;D_6__uncultured bacterium
D_0__Bacteria;D_1__Firmicutes;D_2__Bacilli;D_3__Bacillales;D_4__Staphylococcaceae;D_5__Jeotgalicoccus;D_6__Jeotgalicoccus sp. M3T9B12
D_0__Bacteria;D_1__Fusobacteria;D_2__Fusobacteriia;D_3__Fusobacteriales;D_4__Leptotrichiaceae;D_5__Leptotrichia;D_6__Leptotrichia sp. oral clone FP036
D_0__Bacteria;D_1__Bacteroidetes;D_2__Bacteroidia;D_3__Bacteroidales;D_4__Prevotellaceae;D_5__Alloprevotella;__
D_0__Bacteria;D_1__Bacteroidetes;D_2__Bacteroidia;D_3__Bacteroidales;D_4__Muribaculaceae;D_5__uncultured bacterium;D_6__uncultured bacterium
D_0__Bacteria;D_1__Fusobacteria;D_2__Fusobacteriia;D_3__Fusobacteriales;D_4__Fusobacteriaceae;D_5__Fusobacterium;D_6__Fusobacterium necrophorum subsp. necrophorum
D_0__Bacteria;D_1__Firmicutes;D_2__Bacilli;D_3__Lactobacillales;D_4__Aerococcaceae;D_5__Globicatella;D_6__uncultured bacterium
D_0__Bacteria;D_1__Firmicutes;D_2__Clostridia;D_3__Clostridiales;D_4__Ruminococcaceae;D_5__Ruminococcaceae UCG-002;__
D_0__Bacteria;D_1__Firmicutes;D_2__Clostridia;D_3__Clostridiales;D_4__Christensenellaceae;D_5__Christensenellaceae R-7 group;D_6__uncultured organism
D_0__Bacteria;D_1__Firmicutes;D_2__Clostridia;D_3__Clostridiales;D_4__Ruminococcaceae;D_5__Ruminococcaceae NK4A214 group;__
D_0__Bacteria;D_1__Spirochaetes;D_2__Spirochaetia;D_3__Spirochaetales;D_4__Spirochaetaceae;D_5__Treponema 2;__
D_0__Bacteria;D_1__Bacteroidetes;D_2__Bacteroidia;D_3__Bacteroidales;D_4__Bacteroidaceae;D_5__Bacteroides;D_6__uncultured bacterium
D_0__Bacteria;D_1__Firmicutes;D_2__Bacilli;D_3__Lactobacillales;D_4__Lactobacillaceae;D_5__Lactobacillus;__
D_0__Bacteria;D_1__Bacteroidetes;D_2__Bacteroidia;D_3__Flavobacteriales;D_4__Weeksellaceae;D_5__Bergeyella;D_6__Cloacibacterium sp. canine oral taxon 320
D_0__Bacteria;D_1__Firmicutes;D_2__Clostridia;D_3__Clostridiales;D_4__Christensenellaceae;D_5__Christensenellaceae R-7 group;D_6__uncultured spirochete
D_0__Bacteria;D_1__Firmicutes;D_2__Negativicutes;D_3__Selenomonadales;D_4__Acidaminococcaceae;D_5__Phascolarctobacterium;__
D_0__Bacteria;D_1__Proteobacteria;D_2__Gammaproteobacteria;D_3__Pseudomonadales;D_4__Moraxellaceae;D_5__Moraxella;D_6__Moraxella osloensis
D_0__Bacteria;D_1__Firmicutes;D_2__Clostridia;D_3__Clostridiales;D_4__Peptostreptococcaceae;D_5__Peptostreptococcus;__
D_0__Bacteria;D_1__Bacteroidetes;D_2__Bacteroidia;D_3__Bacteroidales;D_4__Prevotellaceae;__;__
D_0__Bacteria;D_1__Bacteroidetes;D_2__Bacteroidia;D_3__Bacteroidales;D_4__Porphyromonadaceae;D_5__Porphyromonas;D_6__Porphyromonas sp. canine oral taxon 401
D_0__Bacteria;D_1__Bacteroidetes;D_2__Bacteroidia;D_3__Bacteroidales;D_4__Porphyromonadaceae;D_5__Porphyromonas;D_6__Porphyromonas circumdentaria
D_0__Bacteria;D_1__Firmicutes;D_2__Clostridia;D_3__Clostridiales;D_4__Ruminococcaceae;D_5__Ruminococcus 1;__
D_0__Bacteria;D_1__Firmicutes;D_2__Bacilli;D_3__Bacillales;D_4__Family XI;D_5__Gemella;__
D_0__Bacteria;D_1__Bacteroidetes;D_2__Bacteroidia;D_3__Flavobacteriales;D_4__Weeksellaceae;D_5__Soonwooa;D_6__Soonwooa buanensis
D_0__Bacteria;D_1__Proteobacteria;D_2__Gammaproteobacteria;D_3__Pasteurellales;D_4__Pasteurellaceae;D_5__Actinobacillus;D_6__Actinobacillus minor
D_0__Bacteria;D_1__Firmicutes;D_2__Negativicutes;D_3__Selenomonadales;D_4__Veillonellaceae;D_5__Veillonella;__
D_0__Bacteria;D_1__Firmicutes;D_2__Erysipelotrichia;D_3__Erysipelotrichales;D_4__Erysipelotrichaceae;D_5__Turicibacter;__
D_0__Bacteria;D_1__Firmicutes;D_2__Clostridia;D_3__Clostridiales;D_4__Ruminococcaceae;D_5__Ruminococcus 1;D_6__Ruminococcus sp. HUN007
D_0__Bacteria;D_1__Firmicutes;D_2__Clostridia;D_3__Clostridiales;D_4__Ruminococcaceae;D_5__Ruminococcaceae NK4A214 group;D_6__uncultured bacterium
D_0__Bacteria;D_1__Bacteroidetes;D_2__Bacteroidia;D_3__Bacteroidales;D_4__Rikenellaceae;D_5__dgA-11 gut group;D_6__uncultured Bacteroidales bacterium
D_0__Bacteria;D_1__Proteobacteria;D_2__Gammaproteobacteria;D_3__Aeromonadales;D_4__Succinivibrionaceae;D_5__Succinivibrio;__
D_0__Bacteria;D_1__Firmicutes;D_2__Clostridia;D_3__Clostridiales;D_4__Family XIII;D_5__Family XIII AD3011 group;D_6__uncultured organism
D_0__Bacteria;D_1__Bacteroidetes;D_2__Bacteroidia;D_3__Bacteroidales;D_4__Porphyromonadaceae;D_5__Porphyromonas;D_6__Porphyromonas sp. feline oral taxon 109
D_0__Bacteria;D_1__Firmicutes;D_2__Clostridia;D_3__Clostridiales;D_4__Christensenellaceae;D_5__Christensenellaceae R-7 group;__
D_0__Bacteria;D_1__Bacteroidetes;D_2__Bacteroidia;D_3__Flavobacteriales;D_4__Weeksellaceae;D_5__Bergeyella;D_6__Bergeyella porcorum
D_0__Bacteria;D_1__Bacteroidetes;D_2__Bacteroidia;D_3__Flavobacteriales;D_4__Weeksellaceae;D_5__Chryseobacterium;__
D_0__Bacteria;D_1__Firmicutes;D_2__Bacilli;D_3__Bacillales;D_4__Planococcaceae;D_5__Kurthia;D_6__uncultured bacterium

D_0__Bacteria;D_1__Firmicutes;D_2__Bacilli;D_3__Bacillales;D_4__Planococcaceae;D_5__Kurthia;D_6__uncultured bacterium

D_0__Bacteria;D_1__Firmicutes;D_2__Bacilli;D_3__Lactobacillales;D_4__Aerococcaceae;D_5__Ignavigranum;D_6__uncultured bacterium

D_0__Bacteria;D_1__Patescibacteria;D_2__Gracilibacteria;D_3__Absconditabacteriales (SR1);D_4__SR1 bacterium oral taxon 875;D_5__SR1 bacterium oral taxon 875;D_6__SR1 bacterium oral taxon 875

D_0__Bacteria;D_1__Proteobacteria;D_2__Gammaproteobacteria;D_3__Betaproteobacteriales;D_4__Neisseriaceae;D_5__Neisseria;D_6__Neisseria dentiae

D_0__Bacteria;D_1__Proteobacteria;D_2__Gammaproteobacteria;D_3__Enterobacteriales;D_4__Enterobacteriaceae;__;__

D_0__Bacteria;D_1__Bacteroidetes;D_2__Bacteroidia;D_3__Bacteroidales;D_4__Prevotellaceae;D_5__Prevotellaceae NK3B31 group;D_6__metagenome

D_0__Bacteria;D_1__Firmicutes;D_2__Bacilli;D_3__Lactobacillales;D_4__Enterococcaceae;D_5__Enterococcus;__

D_0__Bacteria;D_1__Firmicutes;D_2__Clostridia;D_3__Clostridiales;D_4__Ruminococcaceae;D_5__[Eubacterium] coprostanoligenes group;D_6__uncultured bacterium

D_0__Bacteria;D_1__Firmicutes;D_2__Clostridia;D_3__Clostridiales;D_4__Ruminococcaceae;D_5__Ruminococcaceae UCG-005;D_6__uncultured rumen bacterium

D_0__Bacteria;D_1__Firmicutes;D_2__Bacilli;D_3__Bacillales;D_4__Staphylococcaceae;D_5__Nosocomiicoccus;D_6__uncultured bacterium

D_0__Bacteria;D_1__Bacteroidetes;D_2__Bacteroidia;D_3__Bacteroidales;D_4__p-2534-18B5 gut group;D_5__uncultured bacterium;D_6__uncultured bacterium

D_0__Bacteria;D_1__Proteobacteria;D_2__Gammaproteobacteria;D_3__Betaproteobacteriales;D_4__Burkholderiaceae;D_5__Herbaspirillum;D_6__Herbaspirillum huttiense subsp. huttiense

D_0__Bacteria;D_1__Firmicutes;D_2__Clostridia;D_3__Clostridiales;D_4__Ruminococcaceae;D_5__[Eubacterium] coprostanoligenes group;__

D_0__Bacteria;D_1__Bacteroidetes;D_2__Bacteroidia;D_3__Bacteroidales;D_4__Prevotellaceae;D_5__Prevotella 1;D_6__uncultured bacterium

D_0__Bacteria;D_1__Bacteroidetes;D_2__Bacteroidia;D_3__Flavobacteriales;D_4__Weeksellaceae;D_5__Bergeyella;__

D_0__Bacteria;D_1__Proteobacteria;D_2__Gammaproteobacteria;D_3__Enterobacteriales;D_4__Enterobacteriaceae;D_5__Klebsiella;__

D_0__Bacteria;D_1__Firmicutes;D_2__Clostridia;D_3__Clostridiales;D_4__Peptostreptococcaceae;D_5__Romboutsia;__

D_0__Bacteria;D_1__Firmicutes;D_2__Clostridia;D_3__Clostridiales;D_4__Christensenellaceae;D_5__Christensenellaceae R-7 group;D_6__gut metagenome

D_0__Bacteria;D_1__Firmicutes;D_2__Clostridia;D_3__Clostridiales;D_4__Ruminococcaceae;D_5__[Eubacterium] coprostanoligenes group;D_6__uncultured organism

D_0__Bacteria;D_1__Bacteroidetes;D_2__Bacteroidia;D_3__Bacteroidales;D_4__Prevotellaceae;D_5__Prevotellaceae NK3B31 group;D_6__uncultured Prevotella sp.

D_0__Bacteria;D_1__Firmicutes;D_2__Clostridia;D_3__Clostridiales;D_4__Ruminococcaceae;D_5__Ruminococcaceae UCG-010;D_6__uncultured bacterium

D_0__Bacteria;D_1__Proteobacteria;D_2__Gammaproteobacteria;D_3__Pseudomonadales;D_4__Moraxellaceae;D_5__Enhydrobacter;__

D_0__Bacteria;D_1__Firmicutes;D_2__Bacilli;D_3__Bacillales;D_4__Planococcaceae;D_5__Lysinibacillus;__

D_0__Bacteria;D_1__Firmicutes;D_2__Bacilli;D_3__Bacillales;D_4__Family XI;D_5__Gemella;D_6__uncultured bacterium

D_0__Bacteria;D_1__Bacteroidetes;D_2__Bacteroidia;D_3__Bacteroidales;D_4__Prevotellaceae;D_5__uncultured;D_6__uncultured bacterium

D_0__Bacteria;D_1__Proteobacteria;D_2__Gammaproteobacteria;D_3__Pasteurellales;D_4__Pasteurellaceae;D_5__Pasteurella;D_6__uncultured bacterium

D_0__Bacteria;D_1__Proteobacteria;D_2__Gammaproteobacteria;D_3__Betaproteobacteriales;D_4__Burkholderiaceae;D_5__Herbaspirillum;__

D_0__Bacteria;D_1__Bacteroidetes;D_2__Bacteroidia;D_3__Flavobacteriales;D_4__Flavobacteriaceae;D_5__Flavobacterium;__

D_0__Bacteria;D_1__Actinobacteria;D_2__Actinobacteria;D_3__Propionibacteriales;D_4__Propionibacteriaceae;D_5__Cutibacterium;__

D_0__Bacteria;D_1__Firmicutes;D_2__Erysipelotrichia;D_3__Erysipelotrichales;D_4__Erysipelotrichaceae;D_5__Erysipelotrichaceae UCG-004;__

D_0__Bacteria;D_1__Firmicutes;D_2__Clostridia;D_3__Clostridiales;D_4__Christensenellaceae;D_5__Christensenellaceae R-7 group;D_6__uncultured prokaryote

D_0__Bacteria;D_1__Firmicutes;D_2__Negativicutes;D_3__Selenomonadales;D_4__Veillonellaceae;D_5__Veillonella;D_6__bacterium RA2114

D_0__Bacteria;D_1__Proteobacteria;D_2__Gammaproteobacteria;D_3__Pseudomonadales;D_4__Pseudomonadaceae;D_5__Pseudomonas;__

D_0__Bacteria;D_1__Firmicutes;D_2__Clostridia;D_3__Clostridiales;D_4__Ruminococcaceae;D_5__Ruminococcus 1;D_6__Ruminococcus flavefaciens

D_0__Bacteria;D_1__Firmicutes;D_2__Clostridia;D_3__Clostridiales;D_4__Lachnospiraceae;D_5__Catonella;D_6__uncultured bacterium

D_0__Bacteria;D_1__Bacteroidetes;D_2__Bacteroidia;D_3__Bacteroidales;D_4__Prevotellaceae;D_5__Prevotellaceae UCG-001;D_6__uncultured rumen bacterium

D_0__Bacteria;D_1__Firmicutes;D_2__Bacilli;D_3__Lactobacillales;D_4__Streptococcaceae;D_5__Streptococcus;D_6__Streptococcus plurextorum

D_0__Bacteria;D_1__Firmicutes;D_2__Clostridia;D_3__Clostridiales;D_4__Lachnospiraceae;D_5__Coprococcus 3;__

D_0__Bacteria;D_1__Firmicutes;D_2__Clostridia;D_3__Clostridiales;D_4__Family XI;D_5__Anaerococcus;__

D_0__Bacteria;D_1__Bacteroidetes;D_2__Bacteroidia;D_3__Bacteroidales;D_4__Prevotellaceae;D_5__Prevotella;__

D_0__Bacteria;D_1__Firmicutes;D_2__Bacilli;D_3__Lactobacillales;D_4__Carnobacteriaceae;D_5__Atopostipes;D_6__uncultured bacterium

D_0__Bacteria;D_1__Firmicutes;D_2__Clostridia;D_3__Clostridiales;D_4__Family XI;D_5__Anaerococcus;D_6__uncultured bacterium

D_0__Bacteria;D_1__Firmicutes;D_2__Clostridia;D_3__Clostridiales;D_4__Ruminococcaceae;D_5__Ruminococcaceae UCG-010;__

D_0__Bacteria;D_1__Fusobacteria;D_2__Fusobacteriia;D_3__Fusobacteriales;D_4__Fusobacteriaceae;D_5__Fusobacterium;__

D_0__Bacteria;D_1__Firmicutes;D_2__Bacilli;D_3__Lactobacillales;D_4__Carnobacteriaceae;D_5__Lacticigenium;D_6__uncultured bacterium

D_0__Bacteria;D_1__Firmicutes;D_2__Clostridia;D_3__Clostridiales;D_4__Family XI;D_5__Parvimonas;D_6__uncultured bacterium

D_0__Bacteria;D_1__Firmicutes;D_2__Clostridia;D_3__Clostridiales;D_4__Ruminococcaceae;D_5__Ruminococcaceae UCG-002;D_6__uncultured bacterium

D_0__Bacteria;D_1__Firmicutes;D_2__Clostridia;D_3__Clostridiales;D_4__Peptostreptococcaceae;D_5__Proteocatella;D_6__Frigovirgula sp. canine oral taxon 058

D_0__Bacteria;D_1__Bacteroidetes;D_2__Bacteroidia;D_3__Bacteroidales;D_4__Dysgonomonadaceae;D_5__Dysgonomonas;D_6__uncultured bacterium

D_0__Bacteria;D_1__Proteobacteria;D_2__Alphaproteobacteria;D_3__Sphingomonadales;D_4__Sphingomonadaceae;D_5__Sphingomonas;__

D_0__Bacteria;D_1__Patescibacteria;D_2__Gracilibacteria;D_3__JGI 0000069-P22;D_4__uncultured bacterium;D_5__uncultured bacterium;D_6__uncultured bacterium

D_0__Bacteria;D_1__Firmicutes;D_2__Clostridia;D_3__Clostridiales;D_4__Ruminococcaceae;D_5__Ruminococcaceae UCG-005;D_6__uncultured bacterium

D_0__Bacteria;D_1__Bacteroidetes;D_2__Bacteroidia;D_3__Bacteroidales;D_4__Prevotellaceae;D_5__Prevotella 9;__

D_0__Bacteria;D_1__Firmicutes;D_2__Bacilli;D_3__Lactobacillales;D_4__Enterococcaceae;__;__

D_0__Bacteria;D_1__Firmicutes;D_2__Clostridia;D_3__Clostridiales;D_4__Family XI;D_5__Tissierella;D_6__uncultured bacterium

D_0__Bacteria;D_1__Bacteroidetes;D_2__Bacteroidia;D_3__Bacteroidales;D_4__Prevotellaceae;D_5__Prevotella 2;D_6__uncultured bacterium

D_0__Bacteria;D_1__Firmicutes;D_2__Clostridia;D_3__Clostridiales;D_4__Ruminococcaceae;__;__

D_0__Bacteria;D_1__Proteobacteria;D_2__Gammaproteobacteria;D_3__Pasteurellales;D_4__Pasteurellaceae;__;__

D_0__Bacteria;D_1__Actinobacteria;D_2__Actinobacteria;D_3__Bifidobacteriales;D_4__Bifidobacteriaceae;D_5__Bifidobacterium;D_6__Bifidobacterium longum subsp. longum

D_0__Bacteria;D_1__Proteobacteria;D_2__Gammaproteobacteria;D_3__Pseudomonadales;D_4__Moraxellaceae;D_5__Moraxella;D_6__Moraxella boevrei DSM 14165

D_0__Bacteria;D_1__Firmicutes;D_2__Clostridia;D_3__Clostridiales;D_4__Clostridiales vadinBB60 group;D_5__uncultured bacterium;D_6__uncultured bacterium

D_0__Bacteria;D_1__Firmicutes;D_2__Clostridia;D_3__Clostridiales;D_4__Lachnospiraceae;D_5__Lachnospiraceae NK4A136 group;D_6__uncultured prokaryote

D_0__Bacteria;D_1__Tenericutes;D_2__Mollicutes;D_3__Mycoplasmatales;D_4__Mycoplasmataceae;D_5__Mycoplasma;__

D_0__Bacteria;D_1__Firmicutes;D_2__Bacilli;D_3__Lactobacillales;D_4__Lactobacillaceae;D_5__Lactobacillus;D_6__Lactobacillus ruminis

D_0__Bacteria;D_1__Firmicutes;D_2__Clostridia;D_3__Clostridiales;D_4__Lachnospiraceae;D_5__Roseburia;__

D_0__Bacteria;D_1__Actinobacteria;D_2__Actinobacteria;D_3__Micrococcales;D_4__Micrococcaceae;D_5__Rothia;D_6__uncultured organism

D_0__Bacteria;D_1__Firmicutes;D_2__Clostridia;D_3__Clostridiales;D_4__Lachnospiraceae;D_5__Coprococcus 1;__

D_0__Bacteria;D_1__Proteobacteria;D_2__Gammaproteobacteria;D_3__Betaproteobacteriales;D_4__Burkholderiaceae;D_5__Tepidimonas;__

D_0__Bacteria;D_1__Bacteroidetes;D_2__Bacteroidia;D_3__Bacteroidales;D_4__Prevotellaceae;D_5__Prevotellaceae NK3B31 group;D_6__uncultured bacterium

D_0__Bacteria;D_1__Bacteroidetes;D_2__Bacteroidia;D_3__Bacteroidales;D_4__Tannerellaceae;D_5__Parabacteroides;D_6__Porphyromonadaceae bacterium DJF_B175

D_0__Bacteria;D_1__Bacteroidetes;D_2__Bacteroidia;D_3__Bacteroidales;D_4__Prevotellaceae;D_5__Alloprevotella;D_6__uncultured bacterium

D_0__Bacteria;D_1__Firmicutes;D_2__Negativicutes;D_3__Selenomonadales;D_4__Veillonellaceae;D_5__Megasphaera;D_6__Megasphaera elsdenii

D_0__Bacteria;D_1__Firmicutes;D_2__Clostridia;D_3__Clostridiales;D_4__Ruminococcaceae;D_5__Ruminococcaceae UCG-002;D_6__uncultured rumen bacterium

D_0__Bacteria;D_1__Firmicutes;D_2__Clostridia;D_3__Clostridiales;D_4__Lachnospiraceae;D_5__Acetitomaculum;D_6__uncultured bacterium

D_0__Bacteria;D_1__Firmicutes;D_2__Clostridia;D_3__Clostridiales;D_4__Ruminococcaceae;D_5__Oscillospira;D_6__uncultured bacterium

D_0__Bacteria;D_1__Firmicutes;D_2__Clostridia;D_3__Clostridiales;D_4__Christensenellaceae;D_5__Christensenellaceae R-7 group;D_6__uncultured bacterium

D_0__Bacteria;D_1__Bacteroidetes;D_2__Bacteroidia;D_3__Bacteroidales;D_4__Prevotellaceae;D_5__Prevotellaceae NK3B31 group;__

D_0__Bacteria;D_1__Bacteroidetes;D_2__Bacteroidia;D_3__Flavobacteriales;D_4__Weeksellaceae;D_5__Chryseobacterium;D_6__Chryseobacterium taklimakanense

D_0__Bacteria;D_1__Proteobacteria;D_2__Gammaproteobacteria;D_3__Betaproteobacteriales;D_4__Burkholderiaceae;D_5__Comamonas;__

D_0__Bacteria;D_1__Firmicutes;D_2__Clostridia;D_3__Clostridiales;D_4__Clostridiaceae 1;D_5__Clostridium sensu stricto 12;D_6__Clostridium acidisoli
D_0__Bacteria;D_1__Firmicutes;D_2__Clostridia;D_3__Clostridiales;D_4__Lachnospiraceae;D_5__[Eubacterium] ruminantium group;__
D_0__Bacteria;D_1__Firmicutes;D_2__Clostridia;D_3__Clostridiales;D_4__Peptostreptococcaceae;D_5__Filifactor;D_6__uncultured bacterium
D_0__Bacteria;D_1__Firmicutes;D_2__Clostridia;D_3__Clostridiales;D_4__Lachnospiraceae;D_5__Lachnospiraceae NK4A136 group;__
D_0__Bacteria;D_1__Epsilonbacteraeota;D_2__Campylobacteria;D_3__Campylobacterales;D_4__Campylobacteraceae;D_5__Campylobacter;D_6__Campylobacter mucosalis
D_0__Bacteria;D_1__Bacteroidetes;D_2__Bacteroidia;D_3__Bacteroidales;D_4__Porphyromonadaceae;D_5__Porphyromonas;D_6__Porphyromonas sp. canine oral taxon 361
D_0__Bacteria;D_1__Firmicutes;D_2__Negativicutes;D_3__Selenomonadales;D_4__Veillonellaceae;__;__
D_0__Bacteria;D_1__Firmicutes;D_2__Clostridia;D_3__Clostridiales;D_4__Ruminococcaceae;D_5__Ruminococcaceae UCG-004;D_6__uncultured bacterium
D_0__Bacteria;D_1__Tenericutes;D_2__Mollicutes;D_3__Anaeroplasmatales;D_4__Anaeroplasmataceae;D_5__Anaeroplasma;D_6__uncultured bacterium
D_0__Bacteria;D_1__Proteobacteria;D_2__Gammaproteobacteria;D_3__Enterobacteriales;D_4__Enterobacteriaceae;D_5__Serratia;__
D_0__Bacteria;D_1__Firmicutes;D_2__Clostridia;D_3__Clostridiales;D_4__Ruminococcaceae;D_5__Candidatus Soleaferrea;D_6__uncultured bacterium
D_0__Bacteria;D_1__Fusobacteria;D_2__Fusobacteriia;D_3__Fusobacteriales;D_4__Leptotrichiaceae;D_5__Leptotrichia;__
D_0__Bacteria;D_1__Firmicutes;D_2__Erysipelotrichia;D_3__Erysipelotrichales;D_4__Erysipelotrichaceae;D_5__uncultured;D_6__uncultured bacterium
D_0__Bacteria;D_1__Firmicutes;D_2__Clostridia;D_3__Clostridiales;D_4__Lachnospiraceae;D_5__uncultured;__
D_0__Bacteria;D_1__Firmicutes;D_2__Erysipelotrichia;D_3__Erysipelotrichales;D_4__Erysipelotrichaceae;D_5__uncultured;__
D_0__Bacteria;D_1__Spirochaetes;D_2__Spirochaetia;D_3__Spirochaetales;D_4__Spirochaetaceae;D_5__Treponema 2;D_6__uncultured bacterium
D_0__Bacteria;D_1__Firmicutes;D_2__Bacilli;D_3__Lactobacillales;D_4__Streptococcaceae;D_5__Streptococcus;D_6__Streptococcus salivarius subsp. thermophilus
D_0__Bacteria;D_1__Firmicutes;D_2__Erysipelotrichia;D_3__Erysipelotrichales;D_4__Erysipelotrichaceae;D_5__Dielma;D_6__uncultured bacterium
D_0__Bacteria;D_1__Firmicutes;D_2__Clostridia;D_3__Clostridiales;D_4__Ruminococcaceae;D_5__Oscillibacter;__
D_0__Bacteria;D_1__Firmicutes;D_2__Bacilli;D_3__Bacillales;D_4__Planococcaceae;D_5__Lysinibacillus;D_6__Bacillus ndiopicus
D_0__Bacteria;D_1__Firmicutes;D_2__Bacilli;D_3__Lactobacillales;D_4__Carnobacteriaceae;D_5__Jeotgalibaca;__
D_0__Bacteria;D_1__Firmicutes;D_2__Clostridia;D_3__Clostridiales;D_4__Ruminococcaceae;D_5__Ruminococcaceae UCG-005;D_6__uncultured organism
D_0__Bacteria;D_1__Bacteroidetes;D_2__Bacteroidia;D_3__Bacteroidales;D_4__Rikenellaceae;D_5__Rikenellaceae RC9 gut group;D_6__uncultured beta proteobacterium
D_0__Bacteria;D_1__Firmicutes;D_2__Clostridia;D_3__Clostridiales;D_4__Lachnospiraceae;D_5__Lachnospiraceae AC2044 group;D_6__uncultured bacterium
D_0__Bacteria;D_1__Firmicutes;D_2__Clostridia;D_3__Clostridiales;D_4__Clostridiales vadinBB60 group;D_5__uncultured organism;D_6__uncultured organism
D_0__Bacteria;D_1__Proteobacteria;D_2__Gammaproteobacteria;D_3__Betaproteobacteriales;D_4__Burkholderiaceae;D_5__Ralstonia;__
D_0__Bacteria;D_1__Bacteroidetes;D_2__Bacteroidia;D_3__Bacteroidales;D_4__Paludibacteraceae;D_5__uncultured;D_6__uncultured bacterium
D_0__Bacteria;D_1__Proteobacteria;D_2__Gammaproteobacteria;D_3__Betaproteobacteriales;D_4__Neisseriaceae;D_5__Conchiformibius;D_6__Neisseria canis
D_0__Bacteria;D_1__Proteobacteria;D_2__Gammaproteobacteria;D_3__Enterobacteriales;D_4__Enterobacteriaceae;D_5__Citrobacter;__
D_0__Bacteria;D_1__Firmicutes;D_2__Clostridia;D_3__Clostridiales;D_4__Ruminococcaceae;D_5__[Eubacterium] coprostanoligenes group;D_6__gut metagenome
D_0__Bacteria;D_1__Firmicutes;D_2__Clostridia;D_3__Clostridiales;D_4__Ruminococcaceae;D_5__Oscillibacter;D_6__uncultured bacterium
D_0__Bacteria;D_1__Firmicutes;D_2__Negativicutes;D_3__Selenomonadales;D_4__Veillonellaceae;D_5__uncultured;__
D_0__Bacteria;D_1__Firmicutes;D_2__Clostridia;D_3__Clostridiales;D_4__Lachnospiraceae;D_5__Cellulosilyticum;D_6__uncultured bacterium
D_0__Bacteria;D_1__Bacteroidetes;D_2__Bacteroidia;D_3__Bacteroidales;D_4__Porphyromonadaceae;D_5__Porphyromonas;D_6__Porphyromonas creviorricanis
D_0__Bacteria;D_1__Firmicutes;D_2__Clostridia;D_3__Clostridiales;D_4__Lachnospiraceae;D_5__Dorea;__
D_0__Bacteria;D_1__Deinococcus-Thermus;D_2__Deinococci;D_3__Deinococcales;D_4__Deinococcaceae;D_5__Deinococcus;__
D_0__Bacteria;D_1__Proteobacteria;D_2__Gammaproteobacteria;D_3__Betaproteobacteriales;D_4__Burkholderiaceae;D_5__Burkholderia-Caballeronia-Paraburkholderia;__
D_0__Bacteria;D_1__Firmicutes;D_2__Clostridia;D_3__Clostridiales;D_4__Lachnospiraceae;D_5__Blautia;__
D_0__Bacteria;D_1__Firmicutes;D_2__Clostridia;D_3__Clostridiales;D_4__Ruminococcaceae;D_5__Ruminococcaceae UCG-014;__
D_0__Bacteria;D_1__Epsilonbacteraeota;D_2__Campylobacteria;D_3__Campylobacterales;D_4__Arcobacteraceae;D_5__Arcobacter;__
D_0__Bacteria;D_1__Bacteroidetes;D_2__Bacteroidia;D_3__Bacteroidales;D_4__Prevotellaceae;D_5__Prevotellaceae UCG-003;D_6__uncultured bacterium
D_0__Bacteria;D_1__Firmicutes;D_2__Clostridia;D_3__Clostridiales;D_4__Ruminococcaceae;D_5__Ruminococcaceae UCG-014;D_6__unidentified rumen bacterium 12-124
D_0__Bacteria;D_1__Firmicutes;D_2__Clostridia;D_3__Clostridiales;D_4__Ruminococcaceae;D_5__Ruminococcaceae UCG-014;D_6__uncultured bacterium
D_0__Bacteria;D_1__Proteobacteria;D_2__Alphaproteobacteria;D_3__Rhizobiales;D_4__Xanthobacteraceae;D_5__Bradyrhizobium;__
D_0__Bacteria;D_1__Firmicutes;D_2__Clostridia;D_3__Clostridiales;D_4__Lachnospiraceae;D_5__Oribacterium;D_6__uncultured bacterium
D_0__Bacteria;D_1__Proteobacteria;__;__;__;__;__
D_0__Bacteria;D_1__Firmicutes;D_2__Clostridia;D_3__Clostridiales;D_4__Ruminococcaceae;D_5__uncultured;D_6__gut metagenome
D_0__Bacteria;D_1__Bacteroidetes;D_2__Bacteroidia;D_3__Flavobacteriales;D_4__Weeksellaceae;D_5__uncultured;D_6__Chishuiella sp. YIM 102668
D_0__Bacteria;D_1__Patescibacteria;D_2__Saccharimonadia;D_3__Saccharimonadales;D_4__TM7 phylum sp. oral clone FR058;D_5__TM7 phylum sp. oral clone FR058;D_6__TM7 phylum sp. oral clone FR058
D_0__Bacteria;D_1__Firmicutes;D_2__Clostridia;D_3__Clostridiales;D_4__Ruminococcaceae;D_5__Ruminococcaceae NK4A214 group;D_6__uncultured rumen bacterium
D_0__Bacteria;D_1__Firmicutes;D_2__Clostridia;D_3__Clostridiales;D_4__Lachnospiraceae;D_5__Marvinbryantia;__
D_0__Bacteria;D_1__Bacteroidetes;D_2__Bacteroidia;D_3__Bacteroidales;D_4__Rikenellaceae;D_5__Rikenellaceae RC9 gut group;D_6__wallaby gut metagenome
D_0__Bacteria;D_1__Firmicutes;D_2__Clostridia;D_3__Clostridiales;D_4__Ruminococcaceae;D_5__Ethanoligenens;D_6__uncultured bacterium
D_0__Bacteria;D_1__Firmicutes;D_2__Clostridia;D_3__Clostridiales;D_4__Ruminococcaceae;D_5__Ruminococcaceae UCG-013;D_6__uncultured bacterium
D_0__Bacteria;D_1__Bacteroidetes;D_2__Bacteroidia;D_3__Bacteroidales;D_4__Rikenellaceae;D_5__Rikenellaceae RC9 gut group;D_6__metagenome
D_0__Bacteria;D_1__Actinobacteria;D_2__Actinobacteria;D_3__Corynebacteriales;D_4__Corynebacteriaceae;D_5__Corynebacterium 1;D_6__Corynebacterium urealyticum
D_0__Bacteria;D_1__Proteobacteria;D_2__Alphaproteobacteria;D_3__Rhizobiales;D_4__Rhizobiaceae;D_5__Mesorhizobium;__
D_0__Bacteria;D_1__Firmicutes;D_2__Bacilli;D_3__Bacillales;D_4__Paenibacillaceae;D_5__Paenibacillus;__
D_0__Bacteria;D_1__Firmicutes;D_2__Clostridia;D_3__Clostridiales;D_4__Peptostreptococcaceae;D_5__Intestinibacter;D_6__uncultured bacterium
D_0__Bacteria;D_1__Firmicutes;D_2__Clostridia;D_3__Clostridiales;D_4__Family XII;D_5__Guggenheimella;D_6__uncultured bacterium
D_0__Bacteria;D_1__Firmicutes;D_2__Erysipelotrichia;D_3__Erysipelotrichales;D_4__Erysipelotrichaceae;D_5__Solobacterium;D_6__uncultured Bulleidia sp.
D_0__Bacteria;D_1__Deinococcus-Thermus;D_2__Deinococci;D_3__Thermales;D_4__Thermaceae;D_5__Thermus;__
D_0__Bacteria;D_1__Planctomycetes;D_2__Planctomycetacia;D_3__Pirellulales;D_4__Pirellulaceae;D_5__p-1088-a5 gut group;D_6__uncultured bacterium
D_0__Bacteria;D_1__Firmicutes;D_2__Clostridia;D_3__Clostridiales;D_4__Lachnospiraceae;D_5__XBB1006;__
D_0__Bacteria;D_1__Firmicutes;D_2__Clostridia;D_3__Clostridiales;D_4__Family XIII;__;__
D_0__Bacteria;D_1__Firmicutes;D_2__Clostridia;D_3__Clostridiales;D_4__Ruminococcaceae;D_5__Ruminococcaceae UCG-010;D_6__gut metagenome
D_0__Bacteria;D_1__Firmicutes;D_2__Bacilli;D_3__Lactobacillales;D_4__Lactobacillaceae;D_5__Lactobacillus;D_6__Lactobacillus mucosae
D_0__Bacteria;D_1__Firmicutes;D_2__Clostridia;D_3__Clostridiales;D_4__Lachnospiraceae;D_5__[Eubacterium] hallii group;D_6__uncultured bacterium
D_0__Bacteria;D_1__Firmicutes;D_2__Bacilli;D_3__Lactobacillales;D_4__Carnobacteriaceae;D_5__Carnobacterium;__
D_0__Bacteria;D_1__Epsilonbacteraeota;D_2__Campylobacteria;D_3__Campylobacterales;D_4__Campylobacteraceae;D_5__Campylobacter;D_6__Campylobacter rectus
D_0__Bacteria;D_1__Cyanobacteria;D_2__Oxyphotobacteria;D_3__Chloroplast;__;__;__
D_0__Bacteria;D_1__Firmicutes;D_2__Bacilli;D_3__Bacillales;D_4__Staphylococcaceae;D_5__Staphylococcus;__
D_0__Bacteria;D_1__Bacteroidetes;D_2__Bacteroidia;D_3__Bacteroidales;D_4__Muribaculaceae;D_5__CAG-873;D_6__uncultured bacterium
D_0__Bacteria;D_1__Spirochaetes;D_2__Spirochaetia;D_3__Spirochaetales;D_4__Spirochaetaceae;D_5__Treponema 2;D_6__Treponema berlinense
D_0__Bacteria;D_1__Firmicutes;D_2__Clostridia;D_3__Clostridiales;D_4__Lachnospiraceae;D_5__Roseburia;D_6__metagenome
D_0__Bacteria;D_1__Spirochaetes;D_2__Spirochaetia;D_3__Spirochaetales;D_4__Spirochaetaceae;D_5__Treponema 2;D_6__Treponema porcinum
D_0__Bacteria;D_1__Proteobacteria;D_2__Gammaproteobacteria;D_3__Aeromonadales;D_4__Succinivibrionaceae;D_5__Succinivibrio;D_6__uncultured Succinivibrio sp.
D_0__Bacteria;D_1__Firmicutes;D_2__Clostridia;D_3__Clostridiales;D_4__Ruminococcaceae;D_5__Ruminococcus 2;__

D_0__Bacteria;D_1__Firmicutes;D_2__Clostridia;D_3__Clostridiales;D_4__Clostridiaceae 1;D_5__Proteiniclasticum;__

D_0__Bacteria;D_1__Proteobacteria;D_2__Gammaproteobacteria;D_3__Aeromonadales;D_4__Succinivibrionaceae;D_5__Succinivibrio;D_6__uncultured bacterium

D_0__Bacteria;D_1__Actinobacteria;D_2__Actinobacteria;D_3__Corynebacteriales;D_4__Corynebacteriaceae;D_5__Lawsonella;D_6__uncultured bacterium

D_0__Bacteria;D_1__Bacteroidetes;D_2__Bacteroidia;D_3__Bacteroidales;D_4__Tannerellaceae;D_5__Tannerella;D_6__Tannerella forsythia

D_0__Bacteria;D_1__Bacteroidetes;D_2__Bacteroidia;D_3__Bacteroidales;D_4__Prevotellaceae;D_5__Prevotellaceae UCG-001;D_6__uncultured bacterium

D_0__Bacteria;D_1__Firmicutes;D_2__Clostridia;D_3__Clostridiales;D_4__Ruminococcaceae;D_5__Ruminococcaceae UCG-002;D_6__uncultured organism

D_0__Bacteria;D_1__Firmicutes;D_2__Clostridia;D_3__Clostridiales;D_4__Ruminococcaceae;D_5__Ruminococcaceae NK4A214 group;D_6__gut metagenome

D_0__Bacteria;D_1__Firmicutes;D_2__Clostridia;D_3__Clostridiales;D_4__Lachnospiraceae;D_5__Moryella;D_6__human gut metagenome

D_0__Bacteria;D_1__Proteobacteria;D_2__Gammaproteobacteria;D_3__Pseudomonadales;D_4__Moraxellaceae;__;__

D_0__Bacteria;D_1__Firmicutes;D_2__Clostridia;D_3__Clostridiales;D_4__Ruminococcaceae;D_5__Ruminococcaceae NK4A214 group;D_6__metagenome

D_0__Bacteria;D_1__Proteobacteria;D_2__Gammaproteobacteria;D_3__Pseudomonadales;D_4__Moraxellaceae;D_5__Acinetobacter;D_6__metagenome

D_0__Bacteria;D_1__Firmicutes;D_2__Clostridia;D_3__Clostridiales;D_4__Clostridiaceae 1;D_5__Caloramator;__

D_0__Bacteria;D_1__Firmicutes;D_2__Bacilli;D_3__Lactobacillales;D_4__Carnobacteriaceae;D_5__Jeotgalibaca;D_6__uncultured bacterium

D_0__Bacteria;D_1__Bacteroidetes;D_2__Bacteroidia;D_3__Bacteroidales;D_4__Dysgonomonadaceae;D_5__Dysgonomonas;__

D_0__Bacteria;D_1__Bacteroidetes;D_2__Bacteroidia;D_3__Bacteroidales;__;__;__

D_0__Bacteria;D_1__Firmicutes;D_2__Negativicutes;D_3__Selenomonadales;D_4__Veillonellaceae;D_5__uncultured;D_6__uncultured rumen bacterium

D_0__Bacteria;D_1__Firmicutes;D_2__Bacilli;D_3__Lactobacillales;D_4__Carnobacteriaceae;D_5__Alloiococcus;__

D_0__Bacteria;D_1__Bacteroidetes;D_2__Bacteroidia;D_3__Bacteroidales;D_4__F082;D_5__uncultured bacterium;D_6__uncultured bacterium

D_0__Bacteria;D_1__Firmicutes;D_2__Clostridia;D_3__Clostridiales;D_4__Ruminococcaceae;D_5__Ruminococcaceae UCG-008;D_6__uncultured bacterium

D_0__Bacteria;D_1__Actinobacteria;D_2__Actinobacteria;D_3__Micrococcales;D_4__Micrococcaceae;D_5__Micrococcus;__

D_0__Bacteria;D_1__Firmicutes;D_2__Clostridia;D_3__Clostridiales;D_4__Clostridiaceae 1;D_5__Clostridium sensu stricto 1;D_6__uncultured Clostridium sp.

D_0__Bacteria;D_1__Bacteroidetes;D_2__Bacteroidia;D_3__Bacteroidales;D_4__Prevotellaceae;D_5__Prevotella 1;__

D_0__Bacteria;D_1__Tenericutes;D_2__Mollicutes;D_3__Mollicutes RF39;__;__;__

D_0__Bacteria;D_1__Firmicutes;D_2__Clostridia;D_3__Clostridiales;D_4__Family XIII;D_5__Family XIII AD3011 group;D_6__uncultured bacterium

D_0__Bacteria;D_1__Firmicutes;D_2__Clostridia;D_3__Clostridiales;D_4__Ruminococcaceae;D_5__[Eubacterium] coprostanoligenes group;D_6__uncultured Clostridium sp.

D_0__Bacteria;D_1__Firmicutes;D_2__Erysipelotrichia;D_3__Erysipelotrichales;D_4__Erysipelotrichaceae;D_5__Erysipelotrichaceae UCG-004;D_6__uncultured bacterium

D_0__Bacteria;D_1__Firmicutes;D_2__Bacilli;D_3__Lactobacillales;D_4__Lactobacillaceae;D_5__Lactobacillus;D_6__Lactobacillus nagelii DSM 13675

D_0__Bacteria;D_1__Actinobacteria;D_2__Actinobacteria;__;__;__;__

D_0__Bacteria;D_1__Firmicutes;D_2__Clostridia;D_3__Clostridiales;D_4__Lachnospiraceae;D_5__Lachnospiraceae NK4A136 group;D_6__gut metagenome

D_0__Bacteria;D_1__Spirochaetes;D_2__Spirochaetia;D_3__Spirochaetales;D_4__Spirochaetaceae;D_5__Treponema 2;D_6__Treponema succinifaciens DSM 2489

D_0__Bacteria;D_1__Proteobacteria;D_2__Alphaproteobacteria;D_3__Acetobacterales;D_4__Acetobacteraceae;D_5__Rubritepida;D_6__uncultured bacterium

D_0__Bacteria;D_1__Actinobacteria;D_2__Actinobacteria;D_3__Micrococcales;D_4__Intrasporangiaceae;__;__

D_0__Bacteria;D_1__Proteobacteria;D_2__Alphaproteobacteria;D_3__Rhodobacterales;D_4__Rhodobacteraceae;D_5__Paracoccus;__

D_0__Bacteria;D_1__Firmicutes;D_2__Clostridia;D_3__Clostridiales;D_4__Ruminococcaceae;D_5__[Eubacterium] coprostanoligenes group;D_6__Clostridiales bacterium 42_27

D_0__Bacteria;D_1__Proteobacteria;D_2__Deltaproteobacteria;D_3__Bradymonadales;D_4__uncultured rumen bacterium;D_5__uncultured rumen bacterium;D_6__uncultured rumen bacterium

D_0__Bacteria;D_1__Bacteroidetes;D_2__Bacteroidia;D_3__Bacteroidales;D_4__Bacteroidales RF16 group;D_5__uncultured rumen bacterium;D_6__uncultured rumen bacterium

D_0__Bacteria;D_1__Bacteroidetes;D_2__Bacteroidia;D_3__Bacteroidales;D_4__Prevotellaceae;D_5__Prevotella 7;D_6__Prevotella sp. RS2

D_0__Bacteria;D_1__Firmicutes;D_2__Clostridia;D_3__Clostridiales;D_4__Ruminococcaceae;D_5__Subdoligranulum;__

D_0__Bacteria;D_1__Firmicutes;D_2__Clostridia;D_3__Clostridiales;D_4__Ruminococcaceae;D_5__Ruminococcaceae UCG-014;D_6__gut metagenome

D_0__Bacteria;D_1__Bacteroidetes;D_2__Ignavibacteria;D_3__Ignavibacteriales;D_4__Melioribacteraceae;D_5__IheB3-7;D_6__uncultured bacterium

D_0__Bacteria;D_1__Bacteroidetes;D_2__Bacteroidia;D_3__Bacteroidales;D_4__Bacteroidaceae;D_5__Bacteroides;D_6__Bacteroides fragilis

D_0__Bacteria;D_1__Deinococcus-Thermus;D_2__Deinococci;D_3__Deinococcales;D_4__Deinococcaceae;D_5__Deinococcus;D_6__Deinococcus piscis

D_0__Bacteria;D_1__Patescibacteria;D_2__Parcubacteria;__;__;__;__

D_0__Bacteria;D_1__Bacteroidetes;D_2__Bacteroidia;D_3__Flavobacteriales;D_4__Flavobacteriaceae;D_5__Capnocytophaga;__

D_0__Bacteria;D_1__Proteobacteria;D_2__Gammaproteobacteria;D_3__Betaproteobacteriales;D_4__Neisseriaceae;D_5__Neisseria;__

D_0__Bacteria;D_1__Bacteroidetes;D_2__Bacteroidia;D_3__Bacteroidales;D_4__Dysgonomonadaceae;D_5__Proteiniphilum;D_6__uncultured bacterium

D_0__Bacteria;D_1__Proteobacteria;D_2__Deltaproteobacteria;D_3__Desulfovibrionales;D_4__Desulfovibrionaceae;D_5__Desulfovibrio;D_6__gut metagenome

D_0__Bacteria;D_1__Firmicutes;D_2__Clostridia;D_3__Clostridiales;D_4__Family XI;D_5__Parvimonas;__

D_0__Bacteria;D_1__Cyanobacteria;D_2__Melainabacteria;D_3__Gastranaerophilales;D_4__uncultured bacterium;D_5__uncultured bacterium;D_6__uncultured bacterium

D_0__Bacteria;D_1__Bacteroidetes;D_2__Bacteroidia;D_3__Bacteroidales;D_4__Tannerellaceae;D_5__Parabacteroides;D_6__uncultured bacterium

D_0__Bacteria;D_1__Firmicutes;D_2__Clostridia;D_3__Clostridiales;D_4__Lachnospiraceae;D_5__Lachnoclostridium 12;__

D_0__Bacteria;D_1__Proteobacteria;D_2__Gammaproteobacteria;D_3__Cardiobacteriales;D_4__Cardiobacteriaceae;D_5__uncultured;D_6__Cardiobacterium sp. canine oral taxon 238

D_0__Bacteria;D_1__Firmicutes;D_2__Clostridia;D_3__Clostridiales;D_4__Ruminococcaceae;D_5__Ruminococcaceae UCG-009;D_6__uncultured bacterium

D_0__Bacteria;D_1__Bacteroidetes;D_2__Bacteroidia;D_3__Bacteroidales;D_4__Prevotellaceae;D_5__Prevotellaceae UCG-004;D_6__uncultured bacterium

D_0__Archaea;D_1__Euryarchaeota;D_2__Methanobacteria;D_3__Methanobacteriales;D_4__Methanobacteriaceae;D_5__Methanobrevibacter;D_6__uncultured Methanobrevibacter sp.

D_0__Bacteria;D_1__Proteobacteria;D_2__Gammaproteobacteria;D_3__Betaproteobacteriales;D_4__Burkholderiaceae;D_5__Pelistega;__

D_0__Bacteria;D_1__Proteobacteria;D_2__Gammaproteobacteria;D_3__Betaproteobacteriales;D_4__Burkholderiaceae;D_5__Comamonas;D_6__Comamonas testosteroni

D_0__Bacteria;D_1__Firmicutes;D_2__Clostridia;D_3__Clostridiales;D_4__Family XI;D_5__Peptoniphilus;D_6__uncultured bacterium

D_0__Bacteria;D_1__Proteobacteria;D_2__Gammaproteobacteria;D_3__Betaproteobacteriales;D_4__Chromobacteriaceae;D_5__Gulbenkiania;D_6__Gulbenkiania indica

D_0__Bacteria;D_1__Firmicutes;D_2__Erysipelotrichia;D_3__Erysipelotrichales;D_4__Erysipelotrichaceae;D_5__Catenisphaera;D_6__uncultured bacterium

D_0__Bacteria;D_1__Firmicutes;D_2__Clostridia;D_3__Clostridiales;D_4__Ruminococcaceae;D_5__Ruminococcaceae UCG-013;__

D_0__Bacteria;D_1__Firmicutes;D_2__Clostridia;D_3__Clostridiales;D_4__Ruminococcaceae;D_5__Ruminiclostridium 6;__

D_0__Bacteria;D_1__Firmicutes;D_2__Clostridia;D_3__Clostridiales;D_4__Family XI;D_5__Tissierella;__

D_0__Bacteria;D_1__Firmicutes;D_2__Clostridia;D_3__Clostridiales;D_4__Clostridiaceae 1;D_5__Proteiniclasticum;D_6__uncultured bacterium

D_0__Bacteria;D_1__Firmicutes;D_2__Bacilli;D_3__Bacillales;D_4__Planococcaceae;D_5__Kurthia;D_6__Kurthia gibsonii

D_0__Bacteria;D_1__Synergistetes;D_2__Synergistia;D_3__Synergistales;D_4__Synergistaceae;D_5__Fretibacterium;D_6__Synergistales bacterium canine oral taxon 180

D_0__Bacteria;D_1__Gemmatimonadetes;D_2__Gemmatimonadetes;D_3__Gemmatimonadales;D_4__Gemmatimonadaceae;D_5__uncultured;__

D_0__Bacteria;D_1__Firmicutes;D_2__Clostridia;D_3__Clostridiales;D_4__Lachnospiraceae;D_5__Lachnospira;__

D_0__Bacteria;D_1__Firmicutes;D_2__Clostridia;D_3__Clostridiales;D_4__Lachnospiraceae;D_5__Lachnoclostridium;__

D_0__Bacteria;D_1__Firmicutes;D_2__Bacilli;D_3__Lactobacillales;D_4__Aerococcaceae;D_5__Aerococcus;__

D_0__Bacteria;D_1__Bacteroidetes;D_2__Bacteroidia;D_3__Bacteroidales;D_4__Rikenellaceae;D_5__Rikenellaceae RC9 gut group;D_6__uncultured Bacteroidales bacterium

D_0__Bacteria;D_1__Bacteroidetes;D_2__Bacteroidia;D_3__Bacteroidales;D_4__Prevotellaceae;D_5__Prevotella 9;D_6__uncultured bacterium

D_0__Bacteria;D_1__Firmicutes;D_2__Clostridia;D_3__Clostridiales;D_4__Ruminococcaceae;D_5__Negativibacillus;D_6__uncultured bacterium

D_0__Bacteria;D_1__Firmicutes;D_2__Clostridia;D_3__Clostridiales;D_4__Family XIII;D_5__Family XIII AD3011 group;D_6__uncultured rumen bacterium

D_0__Bacteria;D_1__Bacteroidetes;D_2__Bacteroidia;D_3__Bacteroidales;D_4__Prevotellaceae;D_5__uncultured;D_6__metagenome

D_0__Bacteria;D_1__Proteobacteria;D_2__Gammaproteobacteria;D_3__Betaproteobacteriales;D_4__Burkholderiaceae;D_5__Bordetella;D_6__Bordetella pertussis

D_0__Bacteria;D_1__Firmicutes;D_2__Bacilli;D_3__Lactobacillales;D_4__Aerococcaceae;D_5__Facklamia;D_6__Facklamia ignava CCUG 37419

D_0__Bacteria;D_1__Actinobacteria;D_2__Actinobacteria;D_3__Bifidobacteriales;D_4__Bifidobacteriaceae;D_5__Bifidobacterium;__

D 0 Bacteria;D 1 Proteobacteria;D 2 Gammaproteobacteria;D 3 Xanthomonadales;D 4 Xanthomonadaceae;D 5 Stenotrophomonas;D 6 Stenotrophomonas koreensis

D_0__Bacteria;D_1__Proteobacteria;D_2__Gammaproteobacteria;D_3__Xanthomonadales;D_4__Xanthomonadaceae;D_5__Stenotrophomonas;D_6__Stenotrophomonas koreensis

D_0__Bacteria;D_1__Firmicutes;D_2__Erysipelotrichia;D_3__Erysipelotrichales;D_4__Erysipelotrichaceae;D_5__Erysipelotrichaceae UCG-004;D_6__gut metagenome

D_0__Bacteria;D_1__Bacteroidetes;D_2__Bacteroidia;D_3__Bacteroidales;D_4__Prevotellaceae;D_5__Prevotella 9;D_6__Prevotellaceae bacterium WR041

D_0__Bacteria;D_1__Bacteroidetes;D_2__Bacteroidia;D_3__Chitinophagales;D_4__Chitinophagaceae;__;__

D_0__Bacteria;D_1__Firmicutes;D_2__Clostridia;D_3__Clostridiales;D_4__Clostridiaceae 1;D_5__Clostridium sensu stricto 6;D_6__Clostridium bornimense

D_0__Bacteria;D_1__Proteobacteria;D_2__Gammaproteobacteria;D_3__Pasteurellales;D_4__Pasteurellaceae;D_5__Actinobacillus;D_6__[Actinobacillus] rossii

D_0__Bacteria;D_1__Firmicutes;D_2__Clostridia;D_3__Clostridiales;D_4__Lachnospiraceae;D_5__Butyrivibrio;D_6__uncultured bacterium

D_0__Bacteria;D_1__Firmicutes;D_2__Bacilli;D_3__Bacillales;D_4__Planococcaceae;D_5__Solibacillus;__

D_0__Bacteria;D_1__Tenericutes;D_2__Mollicutes;D_3__Mollicutes RF39;D_4__uncultured bacterium;D_5__uncultured bacterium;D_6__uncultured bacterium

D_0__Bacteria;D_1__Bacteroidetes;D_2__Bacteroidia;D_3__Bacteroidales;D_4__Prevotellaceae;D_5__Prevotella 7;__

D_0__Bacteria;D_1__Firmicutes;D_2__Clostridia;D_3__Clostridiales;D_4__Ruminococcaceae;D_5__Ruminococcaceae UCG-005;D_6__uncultured Ruminococcaceae bacterium

D_0__Bacteria;D_1__Firmicutes;D_2__Clostridia;D_3__Clostridiales;D_4__Ruminococcaceae;D_5__Ruminiclostridium 6;D_6__uncultured bacterium

D_0__Bacteria;D_1__Firmicutes;D_2__Clostridia;D_3__Clostridiales;D_4__JTB215;D_5__bacterium str. 77003;D_6__bacterium str. 77003

D_0__Bacteria;D_1__Firmicutes;D_2__Bacilli;D_3__Bacillales;D_4__Planococcaceae;__;__

D_0__Bacteria;D_1__Bacteroidetes;D_2__Bacteroidia;D_3__Bacteroidales;D_4__Bacteroidales RF16 group;D_5__uncultured bacterium;D_6__uncultured bacterium

D_0__Bacteria;D_1__Actinobacteria;D_2__Actinobacteria;D_3__Micrococcales;D_4__Micrococcaceae;D_5__Rothia;__

D_0__Bacteria;D_1__Tenericutes;D_2__Mollicutes;D_3__Mycoplasmatales;D_4__Mycoplasmataceae;D_5__Mycoplasma;D_6__Mycoplasma parvum str. Indiana

D_0__Bacteria;D_1__Firmicutes;D_2__Negativicutes;D_3__Selenomonadales;D_4__Veillonellaceae;D_5__Anaerovibrio;D_6__uncultured bacterium

D_0__Bacteria;D_1__Proteobacteria;D_2__Gammaproteobacteria;D_3__Betaproteobacteriales;D_4__Burkholderiaceae;D_5__Aquabacterium;__

D_0__Bacteria;D_1__Firmicutes;D_2__Erysipelotrichia;D_3__Erysipelotrichales;D_4__Erysipelotrichaceae;D_5__Holdemanella;__

D_0__Bacteria;D_1__Spirochaetes;D_2__Spirochaetia;D_3__Spirochaetales;D_4__Spirochaetaceae;D_5__Sphaerochaeta;D_6__uncultured bacterium

D_0__Bacteria;D_1__Firmicutes;D_2__Clostridia;D_3__Clostridiales;D_4__Family XI;D_5__W5053;D_6__uncultured bacterium

D_0__Bacteria;D_1__Firmicutes;D_2__Clostridia;D_3__Clostridiales;D_4__Ruminococcaceae;D_5__uncultured;D_6__uncultured bacterium

D_0__Bacteria;D_1__Firmicutes;D_2__Bacilli;D_3__Lactobacillales;D_4__Carnobacteriaceae;D_5__Allofustis;D_6__uncultured bacterium

D_0__Bacteria;D_1__Bacteroidetes;D_2__Bacteroidia;D_3__Bacteroidales;D_4__uncultured;D_5__uncultured bacterium;D_6__uncultured bacterium

D_0__Bacteria;D_1__Kiritimatiellaeota;D_2__Kiritimatiellae;D_3__WCHB1-41;D_4__uncultured bacterium;D_5__uncultured bacterium;D_6__uncultured bacterium

D_0__Bacteria;D_1__Firmicutes;D_2__Negativicutes;D_3__Selenomonadales;D_4__Veillonellaceae;D_5__Anaerovibrio;__

D_0__Bacteria;D_1__Firmicutes;D_2__Clostridia;D_3__Clostridiales;D_4__Ruminococcaceae;D_5__uncultured;D_6__uncultured Clostridium sp.

D_0__Bacteria;D_1__Firmicutes;D_2__Clostridia;D_3__Clostridiales;D_4__Ruminococcaceae;D_5__Ruminiclostridium 9;__

D_0__Bacteria;D_1__Firmicutes;D_2__Clostridia;D_3__Clostridiales;D_4__Lachnospiraceae;D_5__[Eubacterium] ruminantium group;D_6__Clostridiales bacterium DJF_VP35

D_0__Bacteria;D_1__Firmicutes;D_2__Clostridia;D_3__Clostridiales;D_4__Defluviitaleaceae;D_5__Defluviitaleaceae UCG-011;__

D_0__Bacteria;D_1__Proteobacteria;D_2__Alphaproteobacteria;D_3__Rhizobiales;D_4__Hyphomicrobiaceae;D_5__Pedomicrobium;__

D_0__Bacteria;D_1__Firmicutes;D_2__Bacilli;D_3__Bacillales;D_4__Family XII;D_5__Exiguobacterium;__

D_0__Bacteria;D_1__Actinobacteria;D_2__Coriobacteriia;D_3__Coriobacteriales;D_4__Atopobiaceae;D_5__Olsenella;D_6__metagenome

D_0__Bacteria;D_1__Bacteroidetes;D_2__Bacteroidia;D_3__Bacteroidales;D_4__Prevotellaceae;D_5__Prevotella 7;D_6__uncultured rumen bacterium

D_0__Bacteria;D_1__Spirochaetes;D_2__Spirochaetia;D_3__Spirochaetales;D_4__Spirochaetaceae;D_5__uncultured;D_6__Spirochaetaceae bacterium SURF-1

D_0__Bacteria;D_1__Bacteroidetes;D_2__Bacteroidia;D_3__Bacteroidales;D_4__p-251-o5;D_5__uncultured bacterium;D_6__uncultured bacterium

D_0__Bacteria;D_1__Patescibacteria;D_2__Parcubacteria;D_3__Candidatus Kaiserbacteria;D_4__uncultured bacterium;D_5__uncultured bacterium;D_6__uncultured bacterium

D_0__Bacteria;D_1__Firmicutes;D_2__Erysipelotrichia;D_3__Erysipelotrichales;D_4__Erysipelotrichaceae;D_5__[Anaerorhabdus] furcosa group;D_6__uncultured bacterium

D_0__Bacteria;D_1__Firmicutes;D_2__Clostridia;D_3__Clostridiales;D_4__Clostridiales vadinBB60 group;D_5__metagenome;D_6__metagenome

D_0__Bacteria;D_1__Firmicutes;D_2__Bacilli;D_3__Lactobacillales;D_4__Carnobacteriaceae;D_5__uncultured;D_6__uncultured bacterium

D_0__Bacteria;D_1__Fibrobacteres;D_2__Fibrobacteria;D_3__Fibrobacterales;D_4__Fibrobacteraceae;D_5__Fibrobacter;D_6__Fibrobacter sp.

D_0__Bacteria;D_1__Actinobacteria;D_2__Actinobacteria;D_3__Corynebacteriales;D_4__Corynebacteriaceae;D_5__Corynebacterium 1;__

D_0__Bacteria;D_1__Firmicutes;D_2__Clostridia;D_3__Clostridiales;D_4__Lachnospiraceae;D_5__Lachnospiraceae NK4B4 group;D_6__uncultured bacterium

D_0__Bacteria;D_1__Firmicutes;D_2__Clostridia;D_3__Clostridiales;D_4__Lachnospiraceae;D_5__Cellulosilyticum;D_6__uncultured Clostridia bacterium

D_0__Bacteria;D_1__Bacteroidetes;D_2__Bacteroidia;D_3__Flavobacteriales;D_4__Flavobacteriaceae;D_5__Flavobacterium;D_6__uncultured bacterium

D_0__Bacteria;D_1__Bacteroidetes;D_2__Bacteroidia;D_3__Chitinophagales;D_4__Chitinophagaceae;D_5__Sediminibacterium;D_6__uncultured bacterium

D_0__Bacteria;D_1__Firmicutes;D_2__Clostridia;D_3__Clostridiales;D_4__Ruminococcaceae;D_5__Oscillospira;__

D_0__Bacteria;D_1__Firmicutes;D_2__Clostridia;D_3__Clostridiales;D_4__Peptostreptococcaceae;D_5__Proteocatella;D_6__uncultured bacterium

D_0__Bacteria;D_1__Bacteroidetes;D_2__Bacteroidia;D_3__Bacteroidales;D_4__Dysgonomonadaceae;D_5__Proteiniphilum;__

D_0__Bacteria;D_1__Actinobacteria;D_2__Actinobacteria;D_3__Micrococcales;D_4__Micrococcaceae;D_5__Rothia;D_6__unidentified

D_0__Bacteria;D_1__Fusobacteria;D_2__Fusobacteriia;D_3__Fusobacteriales;D_4__Leptotrichiaceae;D_5__Leptotrichia;D_6__uncultured Leptotrichia sp.

D_0__Bacteria;D_1__Firmicutes;D_2__Clostridia;D_3__Clostridiales;D_4__Ruminococcaceae;D_5__uncultured;__

D_0__Bacteria;D_1__Bacteroidetes;D_2__Bacteroidia;D_3__Bacteroidales;D_4__Tannerellaceae;D_5__Macellibacteroides;__

D_0__Bacteria;D_1__Firmicutes;D_2__Bacilli;D_3__Lactobacillales;D_4__Streptococcaceae;D_5__Streptococcus;D_6__Streptococcus hyovaginalis

D_0__Bacteria;D_1__Tenericutes;D_2__Mollicutes;D_3__Izimaplasmatales;D_4__uncultured bacterium;D_5__uncultured bacterium;D_6__uncultured bacterium

D_0__Bacteria;D_1__Proteobacteria;D_2__Alphaproteobacteria;D_3__Rhizobiales;D_4__Xanthobacteraceae;__;__

D_0__Bacteria;D_1__Proteobacteria;D_2__Gammaproteobacteria;D_3__Betaproteobacteriales;D_4__Burkholderiaceae;__;__

D_0__Bacteria;D_1__Firmicutes;D_2__Clostridia;D_3__Clostridiales;D_4__Family XIII;D_5__uncultured;D_6__uncultured bacterium

D_0__Bacteria;D_1__Bacteroidetes;D_2__Bacteroidia;D_3__Bacteroidales;D_4__Prevotellaceae;D_5__Prevotella 7;D_6__uncultured bacterium

D_0__Bacteria;D_1__Bacteroidetes;D_2__Bacteroidia;D_3__Bacteroidales;D_4__Prevotellaceae;D_5__Alloprevotella;D_6__uncultured Prevotellaceae bacterium

D_0__Bacteria;D_1__Firmicutes;D_2__Clostridia;D_3__Clostridiales;D_4__Ruminococcaceae;D_5__Ruminococcus 1;D_6__gut metagenome

D_0__Bacteria;D_1__Firmicutes;D_2__Clostridia;D_3__Clostridiales;D_4__Ruminococcaceae;D_5__Ruminiclostridium 5;D_6__uncultured bacterium

D_0__Bacteria;D_1__Firmicutes;D_2__Bacilli;D_3__Lactobacillales;D_4__Enterococcaceae;D_5__Enterococcus;D_6__Enterococcus sp. MES2

D_0__Bacteria;D_1__Firmicutes;D_2__Bacilli;D_3__Lactobacillales;D_4__Aerococcaceae;D_5__Aerococcus;D_6__Aerococcus suis

D_0__Bacteria;D_1__Bacteroidetes;D_2__Bacteroidia;D_3__Sphingobacteriales;D_4__env.OPS 17;D_5__uncultured Bacteroidetes bacterium;D_6__uncultured Bacteroidetes bacterium

D_0__Bacteria;D_1__Bacteroidetes;D_2__Bacteroidia;D_3__Bacteroidales;D_4__Prevotellaceae;D_5__Prevotella;D_6__Prevotella disiens JCM 6334 = ATCC 29426

D_0__Bacteria;D_1__Bacteroidetes;D_2__Bacteroidia;D_3__Bacteroidales;D_4__Bacteroidaceae;D_5__Bacteroides;D_6__Bacteroides plebeius

D_0__Bacteria;D_1__Spirochaetes;D_2__Spirochaetia;D_3__Spirochaetales;D_4__Spirochaetaceae;D_5__Treponema 2;D_6__Treponema medium

D_0__Bacteria;D_1__Firmicutes;D_2__Negativicutes;D_3__Selenomonadales;D_4__Veillonellaceae;D_5__uncultured;D_6__uncultured rumen bacterium 3C28d-3

D_0__Bacteria;D_1__Bacteroidetes;D_2__Bacteroidia;D_3__Flavobacteriales;D_4__Weeksellaceae;D_5__Empedobacter;D_6__uncultured bacterium

D_0__Bacteria;D_1__Bacteroidetes;D_2__Bacteroidia;D_3__Flavobacteriales;D_4__Flavobacteriaceae;D_5__Capnocytophaga;D_6__uncultured bacterium

D_0__Bacteria;D_1__Bacteroidetes;D_2__Bacteroidia;D_3__Bacteroidales;D_4__Prevotellaceae;D_5__Prevotella 2;D_6__human gut metagenome

D_0__Bacteria;D_1__Actinobacteria;D_2__Actinobacteria;D_3__Micrococcales;D_4__Intrasporangiaceae;D_5__Ornithinimicrobium;D_6__uncultured bacterium

D_0__Bacteria;D_1__Epsilonbacteraeota;D_2__Campylobacteria;D_3__Campylobacterales;D_4__Campylobacteraceae;D_5__Campylobacter;__

D_0__Bacteria;D_1__Bacteroidetes;D_2__Bacteroidia;D_3__Bacteroidales;D_4__Prevotellaceae;D_5__Prevotellaceae YAB2003 group;__

D_0__Bacteria;D_1__Bacteroidetes;D_2__Bacteroidia;D_3__Bacteroidales;D_4__Marinifilaceae;D_5__Butyricimonas;D_6__uncultured bacterium

D_0__Bacteria;D_1__Proteobacteria;D_2__Alphaproteobacteria;D_3__Reyranellales;D_4__Reyranellaceae;D_5__Reyranella;__

D_0__Bacteria;D_1__Chloroflexi;D_2__Anaerolineae;__;__;__;__

D_0__Bacteria;D_1__Actinobacteria;D_2__Actinobacteria;D_3__Micrococcales;D_4__Micrococcaceae;D_5__Kocuria;__
D_0__Bacteria;D_1__Firmicutes;D_2__Clostridia;D_3__Clostridiales;D_4__Peptostreptococcaceae;D_5__uncultured;__
D_0__Bacteria;D_1__Firmicutes;D_2__Clostridia;D_3__Clostridiales;D_4__Lachnospiraceae;D_5__Johnsonella;__
D_0__Bacteria;D_1__Actinobacteria;D_2__Actinobacteria;D_3__Micrococcales;D_4__Brevibacteriaceae;D_5__Brevibacterium;__
D_0__Bacteria;D_1__Actinobacteria;D_2__Actinobacteria;D_3__Actinomycetales;D_4__Actinomycetaceae;D_5__Actinomyces;D_6__Actinomyces hyovaginalis
D_0__Bacteria;D_1__Proteobacteria;D_2__Alphaproteobacteria;D_3__Acetobacterales;D_4__Acetobacteraceae;D_5__Acetobacter;__
D_0__Bacteria;D_1__Patescibacteria;D_2__Parcubacteria;D_3__Candidatus Adlerbacteria;D_4__uncultured bacterium;D_5__uncultured bacterium;D_6__uncultured bacterium
D_0__Bacteria;D_1__Firmicutes;D_2__Clostridia;D_3__Clostridiales;D_4__Lachnospiraceae;D_5__Cellulosilyticum;D_6__bacterium PS7
D_0__Bacteria;D_1__Firmicutes;D_2__Clostridia;D_3__Clostridiales;D_4__Family XIII;D_5__[Eubacterium] nodatum group;D_6__[Eubacterium] sulci
D_0__Bacteria;D_1__Proteobacteria;D_2__Gammaproteobacteria;D_3__Betaproteobacteriales;D_4__Burkholderiaceae;D_5__Sutterella;__
D_0__Bacteria;D_1__Proteobacteria;D_2__Gammaproteobacteria;D_3__Alteromonadales;D_4__Alteromonadaceae;D_5__Rheinheimera;__
D_0__Bacteria;D_1__Firmicutes;D_2__Clostridia;D_3__Clostridiales;D_4__Ruminococcaceae;D_5__Fastidiosipila;D_6__uncultured bacterium
D_0__Bacteria;D_1__Firmicutes;D_2__Clostridia;D_3__Clostridiales;D_4__Lachnospiraceae;D_5__uncultured;D_6__Frisingicoccus caecimuris
D_0__Bacteria;D_1__Firmicutes;D_2__Clostridia;D_3__Clostridiales;D_4__Clostridiaceae 1;D_5__Clostridium sensu stricto 1;D_6__uncultured rumen bacterium
D_0__Bacteria;D_1__Epsilonbacteraeota;D_2__Campylobacteria;D_3__Campylobacterales;D_4__Campylobacteraceae;D_5__Campylobacter;D_6__Campylobacter hyointestinalis subsp. lawsonii
D_0__Bacteria;D_1__Actinobacteria;D_2__Actinobacteria;D_3__Actinomycetales;D_4__Actinomycetaceae;D_5__Trueperella;D_6__uncultured bacterium
D_0__Bacteria;D_1__Proteobacteria;D_2__Gammaproteobacteria;D_3__Pseudomonadales;D_4__Pseudomonadaceae;D_5__Pseudomonas;D_6__Pseudomonas guangdongensis
D_0__Bacteria;D_1__Lentisphaerae;D_2__Oligosphaeria;D_3__Oligosphaerales;D_4__Oligosphaeraceae;D_5__Z20;D_6__uncultured bacterium
D_0__Bacteria;D_1__Firmicutes;D_2__Clostridia;D_3__Clostridiales;D_4__Peptostreptococcaceae;D_5__Proteocatella;D_6__Frigovirgula sp. canine oral taxon 032
D_0__Bacteria;D_1__Firmicutes;D_2__Bacilli;D_3__Bacillales;D_4__Planococcaceae;D_5__Chryseomicrobium;__
D_0__Bacteria;D_1__Actinobacteria;D_2__Coriobacteriia;D_3__Coriobacteriales;D_4__uncultured;D_5__uncultured bacterium;D_6__uncultured bacterium
D_0__Bacteria;D_1__Actinobacteria;D_2__Actinobacteria;D_3__Micrococcales;D_4__Micrococcaceae;__;__
D_0__Bacteria;D_1__Proteobacteria;D_2__Gammaproteobacteria;D_3__Betaproteobacteriales;D_4__Hydrogenophilaceae;D_5__Tepidiphilus;D_6__uncultured bacterium
D_0__Bacteria;D_1__Proteobacteria;D_2__Gammaproteobacteria;D_3__Betaproteobacteriales;D_4__Burkholderiaceae;D_5__Acidovorax;__
D_0__Bacteria;D_1__Firmicutes;D_2__Clostridia;D_3__Clostridiales;D_4__Clostridiaceae 1;D_5__Clostridium sensu stricto 1;D_6__human gut metagenome
D_0__Bacteria;D_1__Proteobacteria;D_2__Gammaproteobacteria;D_3__Pseudomonadales;D_4__Moraxellaceae;D_5__Acinetobacter;D_6__Acinetobacter lwoffii
D_0__Bacteria;D_1__Firmicutes;D_2__Clostridia;D_3__Clostridiales;D_4__Family XI;D_5__Peptoniphilus;D_6__uncultured organism
D_0__Bacteria;D_1__Actinobacteria;D_2__Actinobacteria;D_3__Corynebacteriales;D_4__Corynebacteriaceae;D_5__Corynebacterium 1;D_6__Corynebacterium sp. 1938BRRJ
D_0__Bacteria;D_1__Tenericutes;D_2__Mollicutes;D_3__Mycoplasmatales;D_4__Mycoplasmataceae;D_5__Mycoplasma;D_6__Mycoplasma coccoides
D_0__Bacteria;D_1__Firmicutes;D_2__Clostridia;D_3__Clostridiales;D_4__Peptostreptococcaceae;D_5__Proteocatella;__
D_0__Bacteria;D_1__Firmicutes;D_2__Clostridia;D_3__Clostridiales;D_4__Peptostreptococcaceae;D_5__Peptoanaerobacter;D_6__Peptoanaerobacter stomatis
D_0__Bacteria;D_1__Firmicutes;D_2__Bacilli;D_3__Lactobacillales;D_4__Lactobacillaceae;D_5__Lactobacillus;D_6__Lactobacillus reuteri
D_0__Bacteria;D_1__Bacteroidetes;D_2__Bacteroidia;D_3__Flavobacteriales;D_4__Flavobacteriaceae;D_5__Capnocytophaga;D_6__Capnocytophaga sp. canine oral taxon 329
D_0__Bacteria;D_1__Proteobacteria;D_2__Gammaproteobacteria;D_3__Pseudomonadales;D_4__Moraxellaceae;D_5__Moraxella;D_6__Moraxella sp. canine oral taxon 442
D_0__Bacteria;D_1__Firmicutes;D_2__Clostridia;D_3__Clostridiales;D_4__Ruminococcaceae;D_5__Intestinimonas;D_6__uncultured bacterium
D_0__Bacteria;D_1__Firmicutes;D_2__Clostridia;D_3__Clostridiales;D_4__Ruminococcaceae;D_5__Faecalibacterium;__
D_0__Bacteria;D_1__Firmicutes;D_2__Clostridia;D_3__Clostridiales;D_4__Lachnospiraceae;D_5__Roseburia;D_6__uncultured bacterium
D_0__Bacteria;D_1__Firmicutes;D_2__Clostridia;D_3__Clostridiales;D_4__Lachnospiraceae;D_5__Anaerosporobacter;D_6__uncultured Lachnospiraceae bacterium
D_0__Bacteria;D_1__Firmicutes;D_2__Clostridia;D_3__Clostridiales;D_4__Family XIII;D_5__Family XIII UCG-001;D_6__uncultured bacterium
D_0__Bacteria;D_1__Bacteroidetes;D_2__Bacteroidia;D_3__Sphingobacteriales;D_4__Sphingobacteriaceae;D_5__Sphingobacterium;__
D_0__Bacteria;D_1__Bacteroidetes;D_2__Bacteroidia;D_3__Flavobacteriales;D_4__Weeksellaceae;D_5__Moheibacter;D_6__Moheibacter stercoris
D_0__Bacteria;D_1__Bacteroidetes;D_2__Bacteroidia;D_3__Bacteroidales;D_4__Tannerellaceae;D_5__Parabacteroides;__
D_0__Bacteria;D_1__Bacteroidetes;D_2__Bacteroidia;D_3__Bacteroidales;D_4__Muribaculaceae;D_5__metagenome;D_6__metagenome
D_0__Bacteria;D_1__Proteobacteria;D_2__Gammaproteobacteria;D_3__Pseudomonadales;D_4__Moraxellaceae;D_5__Acinetobacter;D_6__Acinetobacter sp. WCHA30
D_0__Bacteria;D_1__Proteobacteria;D_2__Gammaproteobacteria;D_3__Betaproteobacteriales;D_4__Burkholderiaceae;D_5__Curvibacter;D_6__uncultured beta proteobacterium
D_0__Bacteria;D_1__Firmicutes;D_2__Clostridia;D_3__Clostridiales;D_4__Lachnospiraceae;D_5__[Eubacterium] hallii group;__
D_0__Bacteria;D_1__Firmicutes;D_2__Clostridia;D_3__Clostridiales;D_4__Lachnospiraceae;D_5__Roseburia;D_6__uncultured Lachnospiraceae bacterium
D_0__Bacteria;D_1__Firmicutes;D_2__Bacilli;D_3__Lactobacillales;D_4__Carnobacteriaceae;D_5__Desemzia;__
D_0__Bacteria;D_1__Bacteroidetes;D_2__Bacteroidia;D_3__Flavobacteriales;D_4__Flavobacteriaceae;D_5__Flavobacterium;D_6__Flavobacterium ummariense
D_0__Bacteria;D_1__Proteobacteria;D_2__Alphaproteobacteria;D_3__Rhodospirillales;D_4__uncultured;__;__
D_0__Bacteria;D_1__Proteobacteria;D_2__Alphaproteobacteria;D_3__Rhodospirillales;D_4__uncultured;D_5__gut metagenome;D_6__gut metagenome
D_0__Bacteria;D_1__Firmicutes;D_2__Clostridia;D_3__Clostridiales;D_4__Ruminococcaceae;D_5__[Eubacterium] coprostanoligenes group;D_6__uncultured Ruminococcaceae bacterium
D_0__Bacteria;D_1__Firmicutes;D_2__Clostridia;D_3__Clostridiales;D_4__Ruminococcaceae;D_5__Ruminiclostridium 9;D_6__uncultured organism
D_0__Bacteria;D_1__Epsilonbacteraeota;D_2__Campylobacteria;D_3__Campylobacterales;D_4__Campylobacteraceae;D_5__Campylobacter;D_6__Campylobacter sp.
D_0__Bacteria;D_1__Bacteroidetes;D_2__Bacteroidia;D_3__Flavobacteriales;D_4__Weeksellaceae;D_5__Chryseobacterium;D_6__Chryseobacterium sp. A1-ST2
D_0__Bacteria;D_1__Actinobacteria;D_2__Actinobacteria;D_3__Corynebacteriales;D_4__Corynebacteriaceae;D_5__Corynebacterium;D_6__Corynebacterium pollutisoli
D_0__Bacteria;D_1__Verrucomicrobia;D_2__Verrucomicrobiae;D_3__Methylacidiphilales;D_4__Methylacidiphilaceae;D_5__uncultured;__
D_0__Bacteria;D_1__Proteobacteria;D_2__Gammaproteobacteria;D_3__Pasteurellales;D_4__Pasteurellaceae;D_5__Pasteurella;__
D_0__Bacteria;D_1__Proteobacteria;D_2__Gammaproteobacteria;D_3__Pasteurellales;D_4__Pasteurellaceae;D_5__Mannheimia;D_6__Mannheimia varigena USDA-ARS-USMARC-1296
D_0__Bacteria;D_1__Proteobacteria;D_2__Deltaproteobacteria;D_3__Myxococcales;D_4__mle1-27;D_5__uncultured Myxococcales bacterium;D_6__uncultured Myxococcales bacterium
D_0__Bacteria;D_1__Firmicutes;D_2__Clostridia;D_3__Clostridiales;D_4__Ruminococcaceae;D_5__uncultured;D_6__uncultured rumen bacterium
D_0__Bacteria;D_1__Firmicutes;D_2__Clostridia;D_3__Clostridiales;D_4__Lachnospiraceae;D_5__Coprococcus 2;__
D_0__Bacteria;D_1__Firmicutes;D_2__Clostridia;D_3__Clostridiales;D_4__Family XIII;D_5__Mogibacterium;D_6__uncultured bacterium
D_0__Bacteria;D_1__Bacteroidetes;D_2__Bacteroidia;D_3__Flavobacteriales;D_4__Weeksellaceae;D_5__Empedobacter;__
D_0__Bacteria;D_1__Actinobacteria;D_2__Actinobacteria;D_3__Propionibacteriales;D_4__Propionibacteriaceae;D_5__Tessaracoccus;D_6__uncultured bacterium
D_0__Bacteria;D_1__Proteobacteria;D_2__Gammaproteobacteria;D_3__Pseudomonadales;D_4__Moraxellaceae;D_5__Alkanindiges;__
D_0__Bacteria;D_1__Firmicutes;D_2__Clostridia;D_3__Clostridiales;D_4__Ruminococcaceae;D_5__GCA-900066225;D_6__uncultured bacterium
D_0__Bacteria;D_1__Firmicutes;D_2__Clostridia;D_3__Clostridiales;D_4__Peptococcaceae;D_5__Peptococcus;D_6__uncultured bacterium
D_0__Bacteria;D_1__Firmicutes;D_2__Clostridia;D_3__Clostridiales;D_4__Lachnospiraceae;D_5__Roseburia;D_6__Roseburia sp. 499
D_0__Bacteria;D_1__Firmicutes;D_2__Clostridia;D_3__Clostridiales;D_4__Family XI;D_5__Sedimentibacter;D_6__Sedimentibacter sp. E177
D_0__Bacteria;D_1__Bacteroidetes;D_2__Bacteroidia;D_3__Sphingobacteriales;D_4__ST-12K33;D_5__uncultured bacterium;D_6__uncultured bacterium
D_0__Bacteria;D_1__Bacteroidetes;D_2__Bacteroidia;D_3__Flavobacteriales;D_4__Weeksellaceae;D_5__Empedobacter;D_6__Empedobacter brevis
D_0__Bacteria;D_1__Bacteroidetes;D_2__Bacteroidia;D_3__Chitinophagales;D_4__Chitinophagaceae;D_5__Ferruginibacter;__
D_0__Bacteria;D_1__Tenericutes;D_2__Mollicutes;D_3__Anaeroplasmatales;D_4__Anaeroplasmataceae;D_5__Anaeroplasma;__
D_0__Bacteria;D_1__Proteobacteria;D_2__Gammaproteobacteria;D_3__Betaproteobacteriales;D_4__Burkholderiaceae;D_5__Achromobacter;__
D_0__Bacteria;D_1__Firmicutes;D_2__Negativicutes;D_3__Selenomonadales;D_4__Veillonellaceae;D_5__Dialister;D_6__gut metagenome
D_0__Bacteria;D_1__Firmicutes;D_2__Negativicutes;D_3__Selenomonadales;D_4__Veillonellaceae;D_5__Anaerospora;D_6__uncultured bacterium
D_0__Bacteria;D_1__Fibrobacteres;D_2__Fibrobacteria;D_3__Fibrobacterales;D_4__Fibrobacteraceae;D_5__Fibrobacter;D_6__Fibrobacter sp. UWOS
D_0__Bacteria;D_1__Epsilonbacteraeota;D_2__Campylobacteria;D_3__Campylobacterales;D_4__Helicobacteraceae;D_5__Helicobacter;

D_0__Bacteria;D_1__Epsilonbacteraeota;D_2__Campylobacteria;D_3__Campylobacterales;D_4__Helicobacteraceae;D_5__Helicobacter;__
D_0__Bacteria;D_1__Cyanobacteria;D_2__Oxyphotobacteria;D_3__Chloroplast;D_4__Phaseolus acutifolius (tepary bean);D_5__Phaseolus acutifolius (tepary bean);D_6__Phaseolus acutifolius (tepary bean)
D_0__Bacteria;D_1__Bacteroidetes;D_2__Ignavibacteria;D_3__Kryptoniales;D_4__BSV26;D_5__uncultured green sulfur bacterium GR-296.II.73;D_6__uncultured green sulfur bacterium GR-296.II.73
D_0__Bacteria;D_1__Bacteroidetes;D_2__Bacteroidia;D_3__Flavobacteriales;D_4__Weeksellaceae;D_5__Chryseobacterium;D_6__Chryseobacterium chengduensis
D_0__Bacteria;D_1__Bacteroidetes;D_2__Bacteroidia;D_3__Bacteroidales;D_4__Rikenellaceae;D_5__Rikenellaceae RC9 gut group;D_6__gut metagenome
D_0__Bacteria;D_1__Bacteroidetes;D_2__Bacteroidia;D_3__Bacteroidales;D_4__Dysgonomonadaceae;__;__
D_0__Bacteria;D_1__Actinobacteria;D_2__Actinobacteria;D_3__Micrococcales;D_4__Dermatophilaceae;D_5__Dermatophilus;__
D_0__Bacteria;D_1__Lentisphaerae;D_2__Lentisphaeria;D_3__Victivallales;D_4__vadinBE97;D_5__uncultured bacterium;D_6__uncultured bacterium
D_0__Bacteria;D_1__Firmicutes;D_2__Negativicutes;D_3__Selenomonadales;D_4__Veillonellaceae;D_5__Quinella;D_6__uncultured bacterium
D_0__Bacteria;D_1__Firmicutes;D_2__Clostridia;D_3__Clostridiales;D_4__Ruminococcaceae;D_5__[Eubacterium] coprostanoligenes group;D_6__uncultured rumen bacterium
D_0__Bacteria;D_1__Firmicutes;D_2__Clostridia;D_3__Clostridiales;D_4__Ruminococcaceae;D_5__Caproiciproducens;D_6__uncultured bacterium
D_0__Bacteria;D_1__Firmicutes;D_2__Clostridia;D_3__Clostridiales;D_4__Family XIII;D_5__Family XIII AD3011 group;__
D_0__Bacteria;D_1__Firmicutes;D_2__Bacilli;D_3__Lactobacillales;D_4__Aerococcaceae;__;__
D_0__Bacteria;D_1__Bacteroidetes;D_2__Bacteroidia;D_3__Bacteroidales;D_4__Prevotellaceae;D_5__Prevotellaceae UCG-004;__
D_0__Bacteria;D_1__Bacteroidetes;D_2__Bacteroidia;D_3__Bacteroidales;D_4__Paludibacteraceae;D_5__F0058;__
D_0__Bacteria;D_1__Actinobacteria;D_2__Coriobacteriia;D_3__OPB41;D_4__uncultured actinobacterium;D_5__uncultured actinobacterium;D_6__uncultured actinobacterium
D_0__Bacteria;D_1__Actinobacteria;D_2__Actinobacteria;D_3__Micrococcales;D_4__Bogoriellaceae;D_5__Bogoriella;D_6__uncultured bacterium
D_0__Bacteria;D_1__Actinobacteria;D_2__Actinobacteria;D_3__Kineosporiales;D_4__Kineosporiaceae;D_5__Quadrisphaera;D_6__Quadrisphaera sp.
D_0__Bacteria;D_1__Proteobacteria;D_2__Gammaproteobacteria;D_3__Xanthomonadales;D_4__Xanthomonadaceae;D_5__Luteimonas;D_6__bacterium ASC802
D_0__Bacteria;D_1__Patescibacteria;D_2__Saccharimonadia;D_3__Saccharimonadales;D_4__uncultured compost bacterium;D_5__uncultured compost bacterium;D_6__uncultured compost bacterium
D_0__Bacteria;D_1__Firmicutes;D_2__Negativicutes;D_3__Selenomonadales;D_4__Veillonellaceae;D_5__uncultured;D_6__uncultured rumen bacterium 4C28d-2
D_0__Bacteria;D_1__Firmicutes;D_2__Clostridia;D_3__Clostridiales;D_4__Family XI;__;__
D_0__Bacteria;D_1__Verrucomicrobia;D_2__Verrucomicrobiae;D_3__Verrucomicrobiales;D_4__Akkermansiaceae;D_5__Akkermansia;D_6__uncultured bacterium
D_0__Bacteria;D_1__Proteobacteria;D_2__Deltaproteobacteria;D_3__Desulfovibrionales;D_4__Desulfovibrionaceae;D_5__Desulfovibrio;__
D_0__Bacteria;D_1__Firmicutes;D_2__Clostridia;D_3__Clostridiales;D_4__Peptococcaceae;D_5__uncultured;__
D_0__Bacteria;D_1__Firmicutes;D_2__Clostridia;D_3__Clostridiales;D_4__Lachnospiraceae;D_5__[Eubacterium] xylanophilum group;__
D_0__Bacteria;D_1__Firmicutes;D_2__Clostridia;D_3__Clostridiales;D_4__Family XI;D_5__Ezakiella;__
D_0__Bacteria;D_1__Firmicutes;D_2__Clostridia;D_3__Clostridiales;D_4__Clostridiales vadinBB60 group;__;__
D_0__Bacteria;D_1__Firmicutes;D_2__Bacilli;D_3__Lactobacillales;D_4__Streptococcaceae;D_5__Streptococcus;D_6__Streptococcus sanguinis
D_0__Bacteria;D_1__Epsilonbacteraeota;D_2__Campylobacteria;D_3__Campylobacterales;D_4__Campylobacteraceae;D_5__Campylobacter;D_6__uncultured Campylobacter sp.
D_0__Bacteria;D_1__Chloroflexi;D_2__Anaerolineae;D_3__Anaerolineales;D_4__Anaerolineaceae;D_5__uncultured;D_6__uncultured Longilinea sp.
D_0__Bacteria;D_1__Bacteroidetes;D_2__Bacteroidia;D_3__Bacteroidales;D_4__Prevotellaceae;D_5__Alloprevotella;D_6__uncultured Bacteroidales bacterium
D_0__Bacteria;D_1__Bacteroidetes;D_2__Bacteroidia;D_3__Bacteroidales;D_4__Dysgonomonadaceae;D_5__uncultured;D_6__Petrimonas sp. feline oral taxon 112
D_0__Bacteria;D_1__Actinobacteria;D_2__Coriobacteriia;D_3__Coriobacteriales;D_4__Atopobiaceae;D_5__Atopobium;__
D_0__Bacteria;D_1__Proteobacteria;D_2__Gammaproteobacteria;__;__;__;__
D_0__Bacteria;D_1__Proteobacteria;D_2__Gammaproteobacteria;D_3__Betaproteobacteriales;D_4__TRA3-20;__;__
D_0__Bacteria;D_1__Proteobacteria;D_2__Deltaproteobacteria;D_3__Desulfovibrionales;D_4__Desulfovibrionaceae;D_5__Bilophila;D_6__uncultured bacterium
D_0__Bacteria;D_1__Firmicutes;D_2__Clostridia;D_3__Clostridiales;D_4__Syntrophomonadaceae;D_5__Syntrophomonas;D_6__Syntrophomonas sp. enrichment culture clone D2CL_Bac_16S_Clone14
D_0__Bacteria;D_1__Firmicutes;D_2__Clostridia;D_3__Clostridiales;D_4__Lachnospiraceae;D_5__[Eubacterium] xylanophilum group;D_6__uncultured bacterium
D_0__Bacteria;D_1__Bacteroidetes;D_2__Bacteroidia;D_3__Flavobacteriales;D_4__Weeksellaceae;D_5__Weeksella;D_6__Weeksella sp. FF8
D_0__Bacteria;D_1__Proteobacteria;D_2__Gammaproteobacteria;D_3__Aeromonadales;D_4__Aeromonadaceae;D_5__Aeromonas;__
D_0__Bacteria;D_1__Proteobacteria;D_2__Alphaproteobacteria;D_3__Rickettsiales;D_4__uncultured;D_5__uncultured alpha proteobacterium;D_6__uncultured alpha proteobacterium
D_0__Bacteria;D_1__Proteobacteria;D_2__Alphaproteobacteria;D_3__Rhodospirillales;D_4__Magnetospirillaceae;D_5__Magnetospirillum;__
D_0__Bacteria;D_1__Firmicutes;D_2__Negativicutes;D_3__Selenomonadales;D_4__Veillonellaceae;D_5__Selenomonas;D_6__uncultured rumen bacterium
D_0__Bacteria;D_1__Firmicutes;D_2__Clostridia;D_3__Clostridiales;D_4__Peptococcaceae;D_5__uncultured;D_6__uncultured organism
D_0__Bacteria;D_1__Firmicutes;D_2__Clostridia;D_3__Clostridiales;D_4__Lachnospiraceae;D_5__[Eubacterium] ruminantium group;D_6__bacterium YE64
D_0__Bacteria;D_1__Firmicutes;D_2__Clostridia;D_3__Clostridiales;D_4__Lachnospiraceae;D_5__[Eubacterium] eligens group;__
D_0__Bacteria;D_1__Firmicutes;D_2__Bacilli;D_3__Lactobacillales;D_4__Aerococcaceae;D_5__Facklamia;D_6__uncultured bacterium
D_0__Bacteria;D_1__Bacteroidetes;D_2__Bacteroidia;D_3__Flavobacteriales;D_4__Weeksellaceae;D_5__uncultured Wautersiella sp.;D_6__uncultured Wautersiella sp.
D_0__Bacteria;D_1__Bacteroidetes;D_2__Bacteroidia;D_3__Bacteroidales;D_4__Muribaculaceae;__;__
D_0__Bacteria;D_1__Actinobacteria;D_2__Coriobacteriia;D_3__Coriobacteriales;D_4__Eggerthellaceae;D_5__uncultured;D_6__uncultured bacterium
D_0__Bacteria;D_1__Actinobacteria;D_2__Actinobacteria;D_3__Corynebacteriales;D_4__Corynebacteriaceae;D_5__Corynebacterium 1;D_6__Corynebacterium xerosis
D_0__Bacteria;D_1__Patescibacteria;D_2__ABY1;D_3__Candidatus Magasanikbacteria;D_4__uncultured bacterium;D_5__uncultured bacterium;D_6__uncultured bacterium
D_0__Bacteria;D_1__Lentisphaerae;D_2__Oligosphaeria;D_3__Oligosphaerales;D_4__Oligosphaeraceae;D_5__horsej-a03;D_6__uncultured bacterium
D_0__Bacteria;D_1__Firmicutes;D_2__Clostridia;D_3__Clostridiales;D_4__Ruminococcaceae;D_5__Butyricicoccus;__
D_0__Bacteria;D_1__Firmicutes;D_2__Clostridia;D_3__Clostridiales;D_4__Lachnospiraceae;D_5__Lachnospiraceae UCG-003;D_6__uncultured bacterium
D_0__Bacteria;D_1__Fibrobacteres;D_2__Fibrobacteria;D_3__Fibrobacterales;D_4__Fibrobacteraceae;D_5__Fibrobacter;D_6__Fibrobacter sp. UWB15
D_0__Bacteria;D_1__Bacteroidetes;D_2__Bacteroidia;D_3__Bacteroidales;D_4__Rikenellaceae;D_5__Acetobacteroides;D_6__uncultured bacterium
D_0__Bacteria;D_1__Bacteroidetes;D_2__Bacteroidia;D_3__Bacteroidales;D_4__M2PB4-65 termite group;D_5__uncultured prokaryote;D_6__uncultured prokaryote
D_0__Bacteria;D_1__Tenericutes;D_2__Mollicutes;D_3__Mollicutes RF39;D_4__unidentified rumen bacterium RF9;D_5__unidentified rumen bacterium RF9;D_6__unidentified rumen bacterium RF9
D_0__Bacteria;D_1__Tenericutes;D_2__Mollicutes;D_3__Izimaplasmatales;D_4__gut metagenome;D_5__gut metagenome;D_6__gut metagenome
D_0__Bacteria;D_1__Spirochaetes;D_2__Spirochaetia;D_3__Spirochaetales;D_4__Spirochaetaceae;D_5__Spirochaeta 2;D_6__uncultured bacterium
D_0__Bacteria;D_1__Proteobacteria;D_2__Gammaproteobacteria;D_3__Betaproteobacteriales;D_4__Rhodocyclaceae;D_5__Propionivibrio;D_6__Propionivibrio sp. canine oral taxon 223
D_0__Bacteria;D_1__Proteobacteria;D_2__Gammaproteobacteria;D_3__Betaproteobacteriales;D_4__Burkholderiaceae;D_5__Sutterella;D_6__gut metagenome
D_0__Bacteria;D_1__Patescibacteria;D_2__Saccharimonadia;D_3__Saccharimonadales;D_4__Saccharimonadaceae;D_5__Candidatus Saccharimonas;D_6__uncultured bacterium
D_0__Bacteria;D_1__Firmicutes;D_2__Negativicutes;D_3__Selenomonadales;D_4__Veillonellaceae;D_5__Schwartzia;D_6__uncultured Schwartzia sp.
D_0__Bacteria;D_1__Firmicutes;D_2__Erysipelotrichia;D_3__Erysipelotrichales;D_4__Erysipelotrichaceae;D_5__uncultured;D_6__uncultured Solobacterium sp.
D_0__Bacteria;D_1__Firmicutes;D_2__Clostridia;D_3__Clostridiales;D_4__Ruminococcaceae;D_5__Ruminococcaceae UCG-013;D_6__uncultured Ruminococcaceae bacterium
D_0__Bacteria;D_1__Firmicutes;D_2__Clostridia;D_3__Clostridiales;D_4__Ruminococcaceae;D_5__Ruminococcaceae UCG-013;D_6__uncultured Clostridiales bacterium
D_0__Bacteria;D_1__Firmicutes;D_2__Bacilli;D_3__Lactobacillales;D_4__Streptococcaceae;D_5__Streptococcus;D_6__Streptococcus dysgalactiae subsp. equisimilis GGS_124
D_0__Bacteria;D_1__Firmicutes;D_2__Bacilli;D_3__Bacillales;D_4__Planococcaceae;D_5__Solibacillus;D_6__uncultured bacterium
D_0__Bacteria;D_1__Firmicutes;D_2__BRH-c20a;D_3__uncultured Peptococcaceae bacterium;D_4__uncultured Peptococcaceae bacterium;D_5__uncultured Peptococcaceae bacterium;D_6__uncultured Peptococcaceae bacterium
D_0__Bacteria;D_1__Deinococcus-Thermus;D_2__Deinococci;D_3__Deinococcales;D_4__Deinococcaceae;D_5__Deinococcus;D_6__Deinococcus antarcticus
D_0__Bacteria;D_1__Bacteroidetes;D_2__Bacteroidia;D_3__Bacteroidales;D_4__Prevotellaceae;D_5__Prevotella 7;D_6__Prevotellaceae bacterium DJF_CR25
D_0__Bacteria;D_1__Actinobacteria;D_2__Coriobacteriia;D_3__Coriobacteriales;D_4__Atopobiaceae;D_5__uncultured;D_6__uncultured bacterium
D_0__Bacteria;D_1__Proteobacteria;D_2__Gammaproteobacteria;D_3__Enterobacteriales;D_4__Enterobacteriaceae;D_5__Enterobacter;__
D_0__Bacteria;D_1__Patescibacteria;D_2__Microgenomatia;D_3__Candidatus Pacebacteria;D_4__uncultured bacterium;D_5__uncultured bacterium;D_6__uncultured bacterium
D_0__Bacteria;D_1__Kiritimatiellaeota;D_2__Kiritimatiellae;D_3__WCHB1-41;D_4__uncultured rumen bacterium;D_5__uncultured rumen bacterium;D_6__uncultured rumen bacterium
D_0__Bacteria;D_1__Firmicutes;D_2__Erysipelotrichia;D_3__Erysipelotrichales;D_4__Erysipelotrichaceae;D_5__uncultured;D_6__uncultured rumen bacterium

D_0__Bacteria;D_1__Firmicutes;D_2__Erysipelotrichia;D_3__Erysipelotrichales;D_4__Erysipelotrichaceae;D_5__Erysipelothrix;D_6__uncultured bacterium
D_0__Bacteria;D_1__Firmicutes;D_2__Clostridia;D_3__Clostridiales;D_4__Ruminococcaceae;D_5__Ruminiclostridium 9;D_6__uncultured Clostridia bacterium
D_0__Bacteria;D_1__Firmicutes;D_2__Clostridia;D_3__Clostridiales;D_4__Ruminococcaceae;D_5__Ercella;__
D_0__Bacteria;D_1__Firmicutes;D_2__Clostridia;D_3__Clostridiales;D_4__Peptococcaceae;D_5__uncultured;D_6__uncultured bacterium
D_0__Bacteria;D_1__Firmicutes;D_2__Bacilli;D_3__Bacillales;D_4__Planococcaceae;D_5__Kurthia;__
D_0__Bacteria;D_1__Bacteroidetes;D_2__Bacteroidia;D_3__Bacteroidales;D_4__Rikenellaceae;D_5__dgA-11 gut group;D_6__uncultured bacterium
D_0__Bacteria;D_1__Proteobacteria;D_2__Gammaproteobacteria;D_3__Betaproteobacteriales;D_4__Burkholderiaceae;D_5__Curvibacter;__
D_0__Bacteria;D_1__Proteobacteria;D_2__Alphaproteobacteria;D_3__Dongiales;D_4__Dongiaceae;D_5__Dongia;__
D_0__Bacteria;D_1__Fusobacteria;D_2__Fusobacteriia;D_3__Fusobacteriales;D_4__Fusobacteriaceae;D_5__Fusobacterium;D_6__gut metagenome
D_0__Bacteria;D_1__Firmicutes;D_2__Negativicutes;D_3__Selenomonadales;D_4__Veillonellaceae;D_5__Veillonella;D_6__Veillonella magna
D_0__Bacteria;D_1__Firmicutes;D_2__Negativicutes;D_3__Selenomonadales;D_4__Veillonellaceae;D_5__Selenomonas 1;D_6__uncultured Veillonellaceae bacterium
D_0__Bacteria;D_1__Firmicutes;D_2__Clostridia;D_3__Clostridiales;D_4__Ruminococcaceae;D_5__Ruminococcaceae UCG-014;D_6__unidentified rumen bacterium JW32
D_0__Bacteria;D_1__Firmicutes;D_2__Clostridia;D_3__Clostridiales;D_4__Lachnospiraceae;D_5__Shuttleworthia;D_6__uncultured Lachnospiraceae bacterium
D_0__Bacteria;D_1__Firmicutes;D_2__Clostridia;D_3__Clostridiales;D_4__Lachnospiraceae;D_5__Lachnospiraceae NK3A20 group;__
D_0__Bacteria;D_1__Firmicutes;D_2__Clostridia;D_3__Clostridiales;D_4__Lachnospiraceae;D_5__Coprococcus 2;D_6__uncultured bacterium
D_0__Bacteria;D_1__Firmicutes;D_2__Clostridia;D_3__Clostridiales;D_4__Family XI;D_5__W5053;D_6__uncultured Firmicutes bacterium
D_0__Bacteria;D_1__Deinococcus-Thermus;D_2__Deinococci;D_3__Deinococcales;D_4__Deinococcaceae;D_5__Deinococcus;D_6__unidentified
D_0__Bacteria;D_1__Chloroflexi;D_2__Anaerolineae;D_3__Anaerolineales;D_4__Anaerolineaceae;D_5__uncultured;D_6__bacterium enrichment culture clone BA53
D_0__Bacteria;D_1__Bacteroidetes;D_2__Bacteroidia;D_3__Flavobacteriales;D_4__Flavobacteriaceae;D_5__Flavobacterium;D_6__Flavobacterium suncheonense
D_0__Bacteria;D_1__Bacteroidetes;D_2__Bacteroidia;D_3__Chitinophagales;D_4__Saprospiraceae;D_5__uncultured;D_6__bacterium enrichment culture clone SRAO_34
D_0__Bacteria;D_1__Actinobacteria;D_2__Actinobacteria;D_3__Actinomycetales;D_4__Actinomycetaceae;__;__
D_0__Bacteria;D_1__Actinobacteria;D_2__Actinobacteria;D_3__Actinomycetales;D_4__Actinomycetaceae;D_5__Actinobaculum;D_6__Actinobaculum suis
D_0__Archaea;D_1__Nanoarchaeaeota;D_2__Nanohaloarchaeia;D_3__Deep Sea Euryarchaeotic Group(DSEG);D_4__uncultured archaeon;D_5__uncultured archaeon;D_6__uncultured archaeon
D_0__Bacteria;D_1__Proteobacteria;D_2__Alphaproteobacteria;D_3__Rhodospirillales;D_4__uncultured;D_5__uncultured Rhodospirillaceae bacterium;D_6__uncultured Rhodospirillaceae bacterium
D_0__Bacteria;D_1__Proteobacteria;D_2__Alphaproteobacteria;D_3__Caulobacterales;D_4__Caulobacteraceae;D_5__Brevundimonas;__
D_0__Bacteria;D_1__Firmicutes;D_2__Clostridia;D_3__Clostridiales;D_4__Ruminococcaceae;D_5__Fournierella;D_6__uncultured bacterium
D_0__Bacteria;D_1__Cyanobacteria;D_2__Melainabacteria;D_3__Obscuribacterales;D_4__uncultured bacterium;D_5__uncultured bacterium;D_6__uncultured bacterium
D_0__Bacteria;D_1__Actinobacteria;D_2__Actinobacteria;D_3__Propionibacteriales;D_4__Propionibacteriaceae;D_5__Tessaracoccus;__
D_0__Bacteria;D_1__Acidobacteria;D_2__Acidobacteriia;D_3__Acidobacteriales;D_4__uncultured;__;__
D_0__Bacteria;D_1__Proteobacteria;D_2__Gammaproteobacteria;D_3__Betaproteobacteriales;D_4__Aquaspirillaceae;D_5__Aquaspirillum;D_6__Aquaspirillum sp. 411
D_0__Bacteria;D_1__Firmicutes;D_2__Erysipelotrichia;D_3__Erysipelotrichales;D_4__Erysipelotrichaceae;D_5__Catenisphaera;D_6__uncultured rumen bacterium
D_0__Bacteria;D_1__Firmicutes;D_2__Clostridia;D_3__Clostridiales;D_4__Ruminococcaceae;D_5__Fastidiosipila;D_6__Clostridiales bacterium CAT 12a
D_0__Bacteria;D_1__Firmicutes;D_2__Clostridia;D_3__Clostridiales;D_4__Ruminococcaceae;D_5__Candidatus Soleaferrea;__
D_0__Bacteria;D_1__Firmicutes;D_2__Clostridia;D_3__Clostridiales;D_4__Peptococcaceae;D_5__uncultured;D_6__uncultured rumen bacterium
D_0__Bacteria;D_1__Firmicutes;D_2__Clostridia;D_3__Clostridiales;D_4__Family XIII;D_5__[Eubacterium] brachy group;__
D_0__Bacteria;D_1__Firmicutes;D_2__Clostridia;D_3__Clostridiales;D_4__Clostridiaceae 1;D_5__Clostridium sensu stricto 11;D_6__uncultured bacterium
D_0__Bacteria;D_1__Firmicutes;D_2__Bacilli;D_3__Lactobacillales;D_4__Enterococcaceae;D_5__Vagococcus;__
D_0__Bacteria;D_1__Firmicutes;D_2__Bacilli;D_3__Lactobacillales;D_4__Aerococcaceae;D_5__Facklamia;__
D_0__Bacteria;D_1__Cyanobacteria;D_2__Oxyphotobacteria;D_3__Nostocales;D_4__Chroococcidiopsaceae;D_5__uncultured;D_6__uncultured bacterium
D_0__Bacteria;D_1__Chlamydiae;D_2__Chlamydiae;D_3__Chlamydiales;D_4__Chlamydiaceae;D_5__Chlamydia;D_6__Chlamydia suis
D_0__Bacteria;D_1__Bacteroidetes;D_2__Bacteroidia;D_3__Sphingobacteriales;D_4__AKYH767;D_5__uncultured bacterium;D_6__uncultured bacterium
D_0__Bacteria;D_1__Tenericutes;D_2__Mollicutes;D_3__Mollicutes RF39;D_4__metagenome;D_5__metagenome;D_6__metagenome
D_0__Bacteria;D_1__Proteobacteria;D_2__Gammaproteobacteria;D_3__Pseudomonadales;D_4__Moraxellaceae;D_5__Acinetobacter;D_6__Acinetobacter celticus
D_0__Bacteria;D_1__Proteobacteria;D_2__Gammaproteobacteria;D_3__Betaproteobacteriales;D_4__Chitinibacteraceae;D_5__Formivibrio;D_6__metagenome
D_0__Bacteria;D_1__Proteobacteria;D_2__Gammaproteobacteria;D_3__Betaproteobacteriales;D_4__Burkholderiaceae;D_5__Oxalobacter;D_6__Oxalobacter formigenes
D_0__Bacteria;D_1__Proteobacteria;D_2__Alphaproteobacteria;D_3__Rhizobiales;D_4__Xanthobacteraceae;D_5__uncultured;__
D_0__Bacteria;D_1__Firmicutes;D_2__Clostridia;D_3__Clostridiales;D_4__Ruminococcaceae;D_5__Ruminococcus 2;D_6__uncultured Ruminococcaceae bacterium
D_0__Bacteria;D_1__Firmicutes;D_2__Clostridia;D_3__Clostridiales;D_4__Ruminococcaceae;D_5__Fastidiosipila;__
D_0__Bacteria;D_1__Firmicutes;D_2__Clostridia;D_3__Clostridiales;D_4__Ruminococcaceae;D_5__Fastidiosipila;D_6__uncultured Clostridium sp.
D_0__Bacteria;D_1__Firmicutes;D_2__Clostridia;D_3__Clostridiales;D_4__Lachnospiraceae;D_5__uncultured;D_6__uncultured prokaryote
D_0__Bacteria;D_1__Bacteroidetes;D_2__Bacteroidia;D_3__Flavobacteriales;D_4__Flavobacteriaceae;D_5__Flavobacterium;D_6__Flavobacterium sp. B202
D_0__Bacteria;D_1__Bacteroidetes;D_2__Bacteroidia;D_3__Bacteroidales;D_4__Prevotellaceae;D_5__Prevotella 7;D_6__uncultured Prevotellaceae bacterium
D_0__Bacteria;D_1__Actinobacteria;D_2__Actinobacteria;D_3__Micrococcales;D_4__Dermabacteraceae;D_5__Brachybacterium;__
D_0__Bacteria;D_1__Proteobacteria;D_2__Alphaproteobacteria;D_3__Rhizobiales;D_4__Hyphomicrobiaceae;D_5__Hyphomicrobium;D_6__uncultured Hyphomicrobiaceae bacterium
D_0__Bacteria;D_1__Proteobacteria;D_2__Alphaproteobacteria;D_3__Acetobacterales;D_4__Acetobacteraceae;D_5__Belnapia;__
D_0__Bacteria;D_1__Firmicutes;D_2__Negativicutes;D_3__Selenomonadales;D_4__Veillonellaceae;D_5__Pectinatus;__
D_0__Bacteria;D_1__Firmicutes;D_2__Negativicutes;D_3__Selenomonadales;D_4__Veillonellaceae;D_5__Megasphaera;__
D_0__Bacteria;D_1__Firmicutes;D_2__Clostridia;D_3__Clostridiales;D_4__Ruminococcaceae;D_5__uncultured;D_6__Ruminococcaceae bacterium Marseille-P3738
D_0__Bacteria;D_1__Firmicutes;D_2__Clostridia;D_3__Clostridiales;D_4__Ruminococcaceae;D_5__Ruminococcaceae UCG-010;D_6__uncultured rumen bacterium
D_0__Bacteria;D_1__Firmicutes;D_2__Clostridia;D_3__Clostridiales;D_4__Ruminococcaceae;D_5__Ruminococcaceae UCG-005;D_6__metagenome
D_0__Bacteria;D_1__Firmicutes;D_2__Clostridia;D_3__Clostridiales;D_4__Ruminococcaceae;D_5__Angelakisella;D_6__Angelakisella massiliensis
D_0__Bacteria;D_1__Firmicutes;D_2__Clostridia;D_3__Clostridiales;D_4__Ruminococcaceae;D_5__Acetanaerobacterium;D_6__uncultured bacterium
D_0__Bacteria;D_1__Firmicutes;D_2__Clostridia;D_3__Clostridiales;D_4__Lachnospiraceae;D_5__Lachnospiraceae UCG-009;__
D_0__Bacteria;D_1__Firmicutes;D_2__Bacilli;D_3__Lactobacillales;D_4__Lactobacillaceae;D_5__Lactobacillus;D_6__Lactobacillus amylolyticus
D_0__Bacteria;D_1__Firmicutes;D_2__Bacilli;D_3__Lactobacillales;D_4__Carnobacteriaceae;D_5__Granulicatella;__
D_0__Bacteria;D_1__Deinococcus-Thermus;D_2__Deinococci;D_3__Deinococcales;D_4__Deinococcaceae;D_5__Deinococcus;D_6__uncultured bacterium
D_0__Bacteria;D_1__Bacteroidetes;D_2__Bacteroidia;D_3__Flavobacteriales;D_4__Weeksellaceae;D_5__Chryseobacterium;D_6__uncultured bacterium
D_0__Bacteria;D_1__Bacteroidetes;D_2__Bacteroidia;D_3__Bacteroidales;D_4__Dysgonomonadaceae;D_5__uncultured;D_6__uncultured bacterium
D_0__Bacteria;D_1__Actinobacteria;D_2__Coriobacteriia;D_3__Coriobacteriales;D_4__Coriobacteriaceae;D_5__Collinsella;__
D_0__Bacteria;D_1__Actinobacteria;D_2__Actinobacteria;D_3__Micrococcales;D_4__Micrococcaceae;D_5__Enteractinococcus;__
D_0__Archaea;D_1__Euryarchaeota;D_2__Thermoplasmata;D_3__uncultured;D_4__uncultured archaeon;D_5__uncultured archaeon;D_6__uncultured archaeon
D_0__Bacteria;D_1__Proteobacteria;D_2__Gammaproteobacteria;D_3__Pseudomonadales;D_4__Pseudomonadaceae;D_5__Pseudomonas;D_6__Pseudomonas formosensis
D_0__Bacteria;D_1__Proteobacteria;D_2__Deltaproteobacteria;D_3__Desulfovibrionales;D_4__Desulfovibrionaceae;D_5__Mailhella;D_6__Mailhella massiliensis
D_0__Bacteria;D_1__Patescibacteria;D_2__Gracilibacteria;D_3__Gracilibacteria bacterium oral taxon 873;D_4__Gracilibacteria bacterium oral taxon 873;D_5__Gracilibacteria bacterium oral taxon 873;D_6__Gracilibacteria bacterium oral
D_0__Bacteria;D_1__Firmicutes;D_2__Negativicutes;D_3__Selenomonadales;D_4__Veillonellaceae;D_5__Dialister;__
D_0__Bacteria;D_1__Firmicutes;D_2__Clostridia;D_3__Clostridiales;D_4__Syntrophomonadaceae;__;__
D_0__Bacteria;D_1__Firmicutes;D_2__Clostridia;D_3__Clostridiales;D_4__Ruminococcaceae;D_5__Ruminococcaceae UCG-010;D_6__uncultured Firmicutes bacterium
D_0__Bacteria;D_1__Coprothermobacteraeota;D_2__Coprothermobacteria;D_3__Coprothermobacterales;D_4__Coprothermobacteraceae;D_5__Coprothermobacter;__

D_0__Bacteria;D_1__Bacteroidetes;D_2__Bacteroidia;D_3__Flavobacteriales;D_4__Flavobacteriaceae;D_5__Myroides;D_6__Myroides odoratus
D_0__Bacteria;D_1__Actinobacteria;D_2__Coriobacteriia;D_3__Coriobacteriales;D_4__Atopobiaceae;D_5__Olsenella;__
D_0__Bacteria;D_1__Proteobacteria;D_2__Gammaproteobacteria;D_3__Xanthomonadales;D_4__Xanthomonadaceae;D_5__Luteimonas;__
D_0__Bacteria;D_1__Proteobacteria;D_2__Gammaproteobacteria;D_3__Oceanospirillales;D_4__Halomonadaceae;D_5__Halomonas;__
D_0__Bacteria;D_1__Proteobacteria;D_2__Gammaproteobacteria;D_3__Aeromonadales;D_4__Succinivibrionaceae;D_5__Succinivibrionaceae UCG-001;__
D_0__Bacteria;D_1__Firmicutes;D_2__Erysipelotrichia;D_3__Erysipelotrichales;D_4__Erysipelotrichaceae;D_5__Erysipelothrix;__
D_0__Bacteria;D_1__Firmicutes;D_2__Clostridia;D_3__M55-D21;D_4__Clostridia bacterium enrichment culture clone WSC-8;D_5__Clostridia bacterium enrichment culture clone WSC-8;D_6__Clostridia bacterium enrichment culture clon
D_0__Bacteria;D_1__Firmicutes;D_2__Clostridia;D_3__Clostridiales;D_4__Ruminococcaceae;D_5__Ruminococcaceae V9D2013 group;D_6__uncultured rumen bacterium
D_0__Bacteria;D_1__Firmicutes;D_2__Clostridia;D_3__Clostridiales;D_4__Ruminococcaceae;D_5__Ruminococcaceae UCG-003;__
D_0__Bacteria;D_1__Firmicutes;D_2__Clostridia;D_3__Clostridiales;D_4__Lachnospiraceae;D_5__[Eubacterium] ruminantium group;D_6__uncultured bacterium
D_0__Bacteria;D_1__Firmicutes;D_2__Clostridia;D_3__Clostridiales;D_4__Lachnospiraceae;D_5__Lachnospiraceae ND3007 group;D_6__metagenome
D_0__Bacteria;D_1__Firmicutes;D_2__Clostridia;D_3__Clostridiales;D_4__Lachnospiraceae;D_5__Lachnoanaerobaculum;__
D_0__Bacteria;D_1__Firmicutes;D_2__Bacilli;D_3__Lactobacillales;D_4__Aerococcaceae;D_5__Globicatella;D_6__Globicatella sp. canine oral taxon 218
D_0__Bacteria;D_1__Cyanobacteria;D_2__Melainabacteria;D_3__Gastranaerophilales;__;__;__
D_0__Bacteria;D_1__Chlamydiae;D_2__Chlamydiae;D_3__Chlamydiales;D_4__Simkaniaceae;D_5__uncultured;__
D_0__Bacteria;D_1__Actinobacteria;D_2__Actinobacteria;D_3__Micrococcales;D_4__Dermatophilaceae;D_5__Dermatophilus;D_6__Dermatophilus sp. AD3
D_0__Bacteria;D_1__Verrucomicrobia;D_2__Verrucomicrobiae;D_3__Opitutales;D_4__Puniceicoccaceae;D_5__uncultured;D_6__uncultured bacterium
D_0__Bacteria;D_1__Proteobacteria;D_2__Gammaproteobacteria;D_3__Pasteurellales;D_4__Pasteurellaceae;D_5__Actinobacillus;D_6__Bisgaard Taxon 10
D_0__Bacteria;D_1__Proteobacteria;D_2__Alphaproteobacteria;D_3__uncultured;D_4__uncultured bacterium;D_5__uncultured bacterium;D_6__uncultured bacterium
D_0__Bacteria;D_1__Patescibacteria;D_2__Microgenomatia;D_3__Candidatus Woesebacteria;__;__;__
D_0__Bacteria;D_1__Firmicutes;D_2__Clostridia;D_3__Clostridiales;D_4__Lachnospiraceae;D_5__Roseburia;D_6__gut metagenome
D_0__Bacteria;D_1__Firmicutes;D_2__Clostridia;D_3__Clostridiales;D_4__Family XI;D_5__Peptoniphilus;__
D_0__Bacteria;D_1__Epsilonbacteraeota;D_2__Campylobacteria;D_3__Campylobacterales;D_4__Helicobacteraceae;D_5__Helicobacter;D_6__Helicobacter bilis
D_0__Bacteria;D_1__Bacteroidetes;D_2__Bacteroidia;D_3__Flavobacteriales;D_4__Flavobacteriaceae;D_5__Myroides;__
D_0__Bacteria;D_1__Bacteroidetes;D_2__Bacteroidia;D_3__Bacteroidales;D_4__Prevotellaceae;D_5__Prevotella 7;D_6__uncultured Prevotella sp.
D_0__Bacteria;D_1__Proteobacteria;D_2__Gammaproteobacteria;D_3__Enterobacteriales;D_4__Enterobacteriaceae;D_5__Plesiomonas;__
D_0__Bacteria;D_1__Proteobacteria;D_2__Deltaproteobacteria;D_3__Oligoflexales;D_4__0319-6G20;D_5__uncultured bacterium;D_6__uncultured bacterium
D_0__Bacteria;D_1__Firmicutes;D_2__Erysipelotrichia;D_3__Erysipelotrichales;D_4__Erysipelotrichaceae;D_5__Candidatus Stoquefichus;__
D_0__Bacteria;D_1__Firmicutes;D_2__Clostridia;D_3__Clostridiales;D_4__Lachnospiraceae;D_5__Oribacterium;D_6__uncultured rumen bacterium 4C28d-13
D_0__Bacteria;D_1__Firmicutes;D_2__Clostridia;D_3__Clostridiales;D_4__Family XI;D_5__Murdochiella;__
D_0__Bacteria;D_1__Firmicutes;D_2__Clostridia;D_3__Clostridiales;D_4__Christensenellaceae;D_5__Christensenellaceae R-7 group;D_6__uncultured Clostridia bacterium
D_0__Bacteria;D_1__Actinobacteria;D_2__Actinobacteria;D_3__Propionibacteriales;D_4__Propionibacteriaceae;D_5__Propioniciclava;__
D_0__Bacteria;D_1__Actinobacteria;D_2__Actinobacteria;D_3__Micrococcales;D_4__Microbacteriaceae;D_5__Microbacterium;__
D_0__Bacteria;D_1__Actinobacteria;D_2__Actinobacteria;D_3__Corynebacteriales;D_4__Corynebacteriaceae;__;__
D_0__Bacteria;D_1__Tenericutes;D_2__Mollicutes;D_3__Mollicutes RF39;D_4__unidentified rumen bacterium RF39;D_5__unidentified rumen bacterium RF39;D_6__unidentified rumen bacterium RF39
D_0__Bacteria;D_1__Proteobacteria;D_2__Gammaproteobacteria;D_3__WD260;D_4__uncultured bacterium;D_5__uncultured bacterium;D_6__uncultured bacterium
D_0__Bacteria;D_1__Proteobacteria;D_2__Gammaproteobacteria;D_3__Cardiobacteriales;D_4__Cardiobacteriaceae;D_5__Suttonella;D_6__Suttonella indologenes
D_0__Bacteria;D_1__Proteobacteria;D_2__Gammaproteobacteria;D_3__Betaproteobacteriales;D_4__Rhodocyclaceae;D_5__Denitratisoma;__
D_0__Bacteria;D_1__Proteobacteria;D_2__Gammaproteobacteria;D_3__Betaproteobacteriales;D_4__Neisseriaceae;D_5__Alysiella;D_6__uncultured bacterium
D_0__Bacteria;D_1__Firmicutes;D_2__Negativicutes;D_3__Selenomonadales;D_4__Veillonellaceae;D_5__Schwartzia;D_6__uncultured rumen bacterium
D_0__Bacteria;D_1__Firmicutes;D_2__Negativicutes;D_3__Selenomonadales;D_4__Veillonellaceae;D_5__Megasphaera;D_6__uncultured bacterium
D_0__Bacteria;D_1__Firmicutes;D_2__Clostridia;D_3__Clostridiales;D_4__Lachnospiraceae;D_5__[Ruminococcus] torques group;__
D_0__Bacteria;D_1__Firmicutes;D_2__Clostridia;D_3__Clostridiales;D_4__Lachnospiraceae;D_5__Lachnospiraceae UCG-010;D_6__uncultured bacterium
D_0__Bacteria;D_1__Firmicutes;D_2__Clostridia;D_3__Clostridiales;D_4__Lachnospiraceae;D_5__CAG-56;D_6__uncultured bacterium
D_0__Bacteria;D_1__Firmicutes;D_2__Clostridia;D_3__Clostridiales;D_4__Clostridiaceae 1;D_5__Clostridium sensu stricto 13;__
D_0__Bacteria;D_1__Actinobacteria;D_2__Actinobacteria;D_3__Bifidobacteriales;D_4__Bifidobacteriaceae;D_5__Bifidobacterium;D_6__Bifidobacterium minimum
D_0__Bacteria;D_1__Actinobacteria;D_2__Acidimicrobiia;D_3__Microtrichales;D_4__uncultured;D_5__wastewater metagenome;D_6__wastewater metagenome
D_0__Bacteria;D_1__Thermotogae;D_2__Thermotogae;D_3__Petrotogales;D_4__Petrotogaceae;D_5__Defluviitoga;D_6__uncultured bacterium
D_0__Bacteria;D_1__Spirochaetes;D_2__Spirochaetia;D_3__Spirochaetales;D_4__Spirochaetaceae;D_5__Sphaerochaeta;D_6__uncultured prokaryote
D_0__Bacteria;D_1__Proteobacteria;D_2__Gammaproteobacteria;D_3__Betaproteobacteriales;D_4__Burkholderiaceae;D_5__Variovorax;__
D_0__Bacteria;D_1__Firmicutes;D_2__Clostridia;D_3__Clostridiales;D_4__Ruminococcaceae;D_5__Ruminococcaceae UCG-013;D_6__uncultured Firmicutes bacterium
D_0__Bacteria;D_1__Firmicutes;D_2__Clostridia;D_3__Clostridiales;D_4__Ruminococcaceae;D_5__Ruminococcaceae UCG-010;D_6__uncultured organism
D_0__Bacteria;D_1__Firmicutes;D_2__Clostridia;D_3__Clostridiales;D_4__Ruminococcaceae;D_5__Ruminococcaceae UCG-010;D_6__uncultured eubacterium WCHB1-54
D_0__Bacteria;D_1__Firmicutes;D_2__Clostridia;D_3__Clostridiales;D_4__Christensenellaceae;D_5__Christensenellaceae R-7 group;D_6__bacterium YE57
D_0__Bacteria;D_1__Bacteroidetes;D_2__Bacteroidia;D_3__Flavobacteriales;D_4__Weeksellaceae;D_5__Cloacibacterium;__
D_0__Bacteria;D_1__Bacteroidetes;D_2__Bacteroidia;D_3__Bacteroidales;D_4__Prevotellaceae;D_5__Prevotellaceae UCG-003;__
D_0__Bacteria;D_1__Actinobacteria;D_2__Coriobacteriia;D_3__Coriobacteriales;D_4__Eggerthellaceae;D_5__Enterorhabdus;D_6__uncultured bacterium
D_0__Bacteria;D_1__Actinobacteria;D_2__Actinobacteria;D_3__Corynebacteriales;D_4__Nocardiaceae;D_5__Rhodococcus;__
D_0__Bacteria;D_1__Proteobacteria;D_2__Gammaproteobacteria;D_3__Pseudomonadales;D_4__Pseudomonadaceae;D_5__Thiopseudomonas;D_6__Pseudomonas sp. M-08
D_0__Bacteria;D_1__Proteobacteria;D_2__Gammaproteobacteria;D_3__Betaproteobacteriales;D_4__Rhodocyclaceae;D_5__Azoarcus;D_6__Azoarcus tolulyticus
D_0__Bacteria;D_1__Proteobacteria;D_2__Gammaproteobacteria;D_3__Betaproteobacteriales;D_4__Burkholderiaceae;D_5__Comamonas;D_6__uncultured bacterium
D_0__Bacteria;D_1__Proteobacteria;D_2__Deltaproteobacteria;D_3__Syntrophobacterales;D_4__Syntrophobacteraceae;D_5__Syntrophobacter;__
D_0__Bacteria;D_1__Firmicutes;D_2__Clostridia;D_3__Clostridiales;D_4__Ruminococcaceae;D_5__Ruminococcaceae UCG-010;D_6__bacterium enrichment culture clone BBMC-9
D_0__Bacteria;D_1__Chloroflexi;D_2__Anaerolineae;D_3__Anaerolineales;D_4__Anaerolineaceae;D_5__uncultured;D_6__uncultured eubacterium WCHB1-31
D_0__Bacteria;D_1__Bacteroidetes;D_2__Bacteroidia;D_3__Bacteroidales;D_4__Bacteroidetes vadinHA17;D_5__uncultured bacterium;D_6__uncultured bacterium
D_0__Bacteria;D_1__Actinobacteria;D_2__Actinobacteria;D_3__Micrococcales;D_4__Dermacoccaceae;D_5__Yimella;D_6__Dermacoccus sp. BSi20643
D_0__Bacteria;D_1__Synergistetes;D_2__Synergistia;D_3__Synergistales;D_4__Synergistaceae;D_5__Acetomicrobium;D_6__uncultured bacterium
D_0__Bacteria;D_1__Proteobacteria;D_2__Deltaproteobacteria;D_3__MBNT15;D_4__uncultured Myxococcales bacterium;D_5__uncultured Myxococcales bacterium;D_6__uncultured Myxococcales bacterium
D_0__Bacteria;D_1__Planctomycetes;D_2__Planctomycetacia;D_3__Pirellulales;D_4__Pirellulaceae;D_5__uncultured;D_6__uncultured bacterium
D_0__Bacteria;D_1__Patescibacteria;D_2__Saccharimonadia;D_3__Saccharimonadales;__;__;__
D_0__Bacteria;D_1__Firmicutes;D_2__Clostridia;D_3__Clostridiales;D_4__Ruminococcaceae;D_5__Ruminococcaceae UCG-010;D_6__metagenome
D_0__Bacteria;D_1__Actinobacteria;D_2__Actinobacteria;D_3__Micrococcales;D_4__Sanguibacteraceae;D_5__Sanguibacter;__
D_0__Bacteria;D_1__Proteobacteria;D_2__Gammaproteobacteria;D_3__Methylococcales;D_4__Methylococcaceae;D_5__uncultured;D_6__uncultured bacterium
D_0__Bacteria;D_1__Firmicutes;D_2__Clostridia;D_3__Clostridiales;D_4__Family XI;D_5__Helcococcus;D_6__uncultured bacterium
D_0__Bacteria;D_1__Firmicutes;D_2__Bacilli;D_3__Lactobacillales;D_4__Streptococcaceae;D_5__Lactococcus;__
D_0__Bacteria;D_1__Bacteroidetes;D_2__Bacteroidia;D_3__Bacteroidales;D_4__Porphyromonadaceae;D_5__Porphyromonas;D_6__Porphyromonas sp. UQD 424
D_0__Bacteria;D_1__Actinobacteria;D_2__Actinobacteria;D_3__Propionibacteriales;D_4__Nocardioidaceae;D_5__Nocardioides;__
